# Kinetics and Mechanism of Enantioselective Cu-Catalyzed Alcohol Silylation

**DOI:** 10.1021/jacs.5c14937

**Published:** 2025-11-20

**Authors:** Pedro H. Helou de Oliveira, Jan Seliger, Shoutong Rao, Guy C. Lloyd-Jones, Guoqiang Wang, Martin Oestreich

**Affiliations:** † Institut für Chemie, Technische Universität Berlin, 10623 Berlin, Germany; ‡ University of Edinburgh, School of Chemistry, Joseph Black Building, David Brewster Road, Edinburgh, EH9 3FJ, U.K.; § State Key Laboratory of Coordination Chemistry, School of Chemistry and Chemical Engineering, 12581 Nanjing University, Nanjing 210023, P. R. China

## Abstract

The enantioselective Cu-catalyzed dehydrogenative Si–O coupling of secondary benzylic alcohols with (*n*Bu)_3_SiH was investigated using a combination of in situ ^1^H/^19^F NMR spectroscopic reaction monitoring, isotopic labeling, kinetic modeling, and computational studies. Macrokinetic behavior is governed by substrate-inhibited L*CuOR·ROH resting states: rates rise with conversion when [(*n*Bu)_3_SiH] > [ROH] and fall when [(*n*Bu)_3_SiH] < [ROH]. Alcohols bearing electron-withdrawing substituents are stronger inhibitors and show overall lower macrokinetic reactivity, but react faster than alcohols with electron-donating substituents in intermolecular competitions, indicating that inhibition is more substituent-sensitive than the product-committing step. Divergence between intrinsic enantioselectivity and observed macrokinetic rates of enantiomers in isolation results from enantiomer-dependent inhibition, and a product-committing σ-bond-metathesis step is consistent with measured Eyring activation parameters and a Si–H/Si–D KIE ≤ 1.3. Eyring and Hammett analyses, as well as DFT calculations, support an H-bonding inhibition mode for the L*CuOR·ROH resting state. Stoichiometric styrene as an additive suppresses H_2_ generation and mitigates catalyst deactivation, increasing process safety and efficiency. Dynamic kinetic resolution, enabled by addition of a ruthenium racemization cocatalyst, results in reaction rates comparable to those of the faster enantiomer while improving overall efficiency.

## Graphical abstract

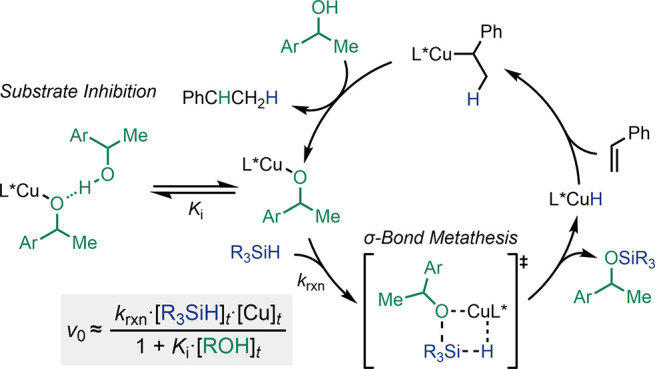

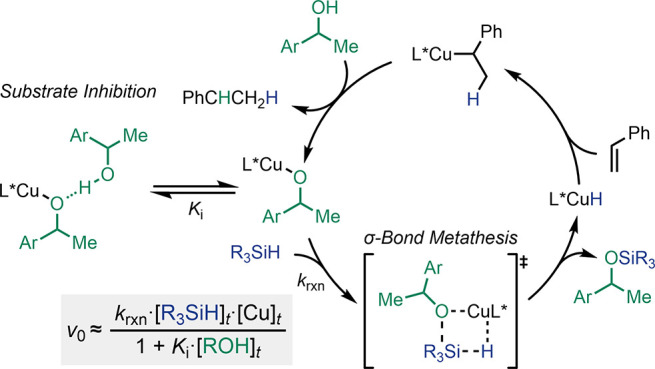

## Introduction

Copper hydride-catalyzed transformations play an important role in modern synthetic organic chemistry.^1^ In 1984, Brunner and Miehling reported on an asymmetric hydrosilylation of acetophenone using diphenylsilane, catalytic CuO*t*Bu, and chiral phosphine ligands such as (−)-diop.^2,3^ Pioneering contributions by Stryker and co-workers in 1988, established hexameric [(Ph_3_P)­CuH]_6_ as a stoichiometric reagent for the conjugate reduction of α,β-unsaturated carbonyl compounds.^4^ A decade later, Lipshutz and co-workers identified hydrosilanes as mild terminal reducing agents for the in situ regeneration of the reactive copper hydride species from a postulated copper enolate intermediate.^2,5^ Based on these discoveries, a substantial body of work has since been developed, including asymmetric reactions which utilize modular precatalyst systems generated from copper salts and chiral phosphine ligands.^6^ Early research efforts in this area focused on enantioselective hydrosilylation of ketones and conjugate reduction of α,β-unsaturated carbonyl compounds (Scheme 1A and B).^7,8^ The latter set the stage for both enantio- and diastereoselective reductive coupling reactions of activated alkenes and carbon electrophiles^9^ as well as (formal) hydrofunctionalizations^10^ of activated and unactivated alkenes (Scheme 1C).^11–13^

**Scheme 1:**
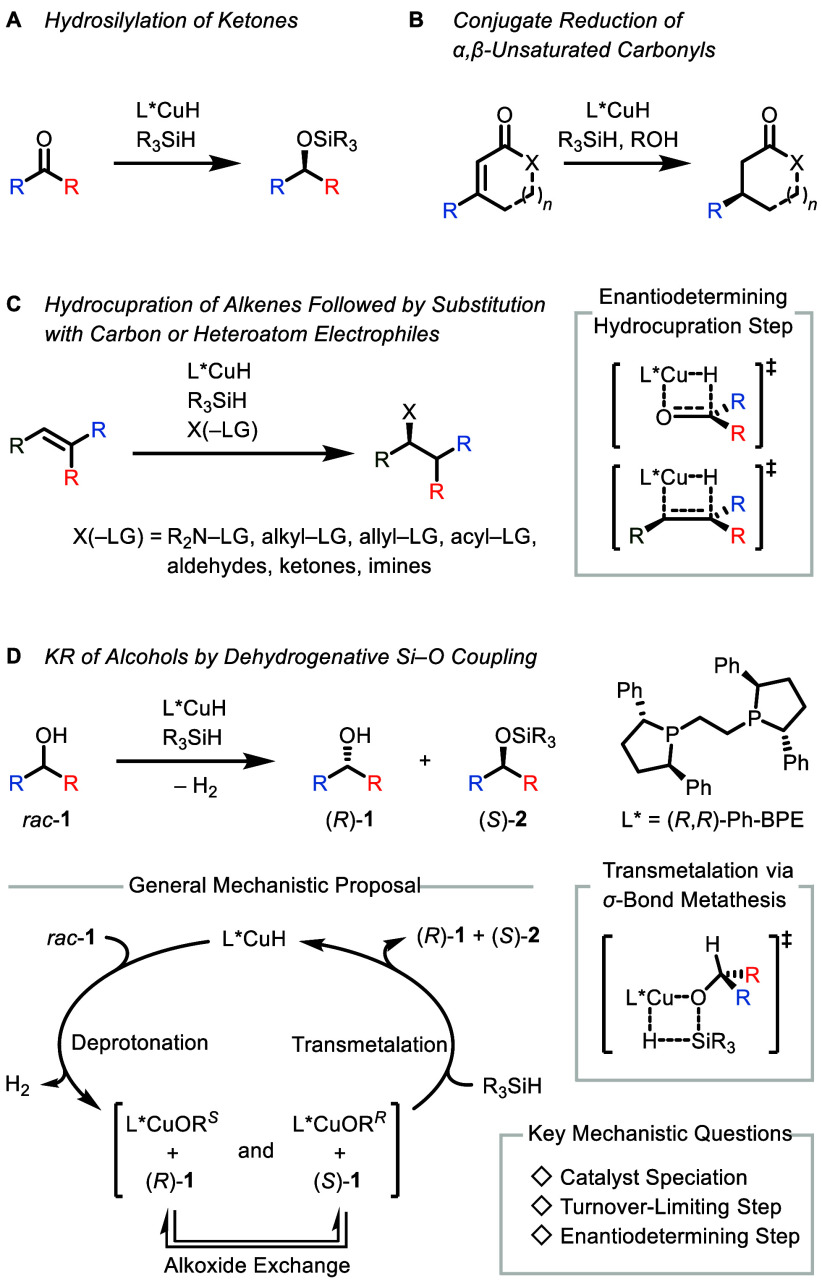
Classes of Asymmetric Copper Hydride-Catalyzed Transformations and Selected Key Mechanistic Features

Our laboratory has a long-standing interest in the application of Cu–H catalysis in the kinetic resolution (KR) of alcohols by stereoselective dehydrogenative Si–O coupling with hydrosilanes (Scheme 1D).^14^ In 2017, we disclosed that a precatalyst system consisting of commercially available CuCl, NaO*t*Bu, and (*R*,*R*)-Ph-BPE, effects the enantioselective silylation of secondary racemic benzylic and allylic alcohols using (*n*Bu)_3_SiH.^14g^ We have also developed a protocol for nonenzymatic dynamic kinetic resolution (DKR) of secondary benzylic alcohols, in which the Cu–H-catalyzed process proceeds in tandem with a rapid in situ racemization of the alcohol effected by a bifunctional ruthenium pincer complex.^14j^

A generic mechanism for these transformations, based on literature precedent,^8a–8c,15–17^ individual experimental findings,^14^ and quantum-chemical calculations on a truncated system,^14d^ is shown in Scheme 1. Accordingly, both enantiomers of the alcohol **1** are deprotonated by the chiral copper hydride complex L*CuH, liberating dihydrogen and giving rise to diastereomeric copper-alkoxide complexes L*CuOR^*S*^ and L*CuOR^*R*^. These undergo rapid exchange with both enantiomers of the bulk alcohol **1**, resulting in a nondegenerate dynamic equilibrium system of the form [L*CuOR^*S*^ + (*R*)-**1** ⇋ L*CuOR^*R*^ + (*S*)-**1**]. The diastereomeric copper alkoxides undergo transmetalation with the hydrosilane to liberate silyl ethers (*S*)-**2** and (*R*)-**2** and regenerate the copper hydride L*CuH. The transmetalation is postulated to involve σ-bond metathesis, with retention of configuration at carbon and silicon, in what is hypothesized to be the enantiodetermining step.^6^ ^d,^ ^6^ ^m^ The latter conclusion is supported by the influence of hydrosilane substituents on the enantioselectivity.^14^ While the existing evidence supports the overarching features of the process (Scheme 1), a detailed understanding of the mechanism has not been achieved. In this context, we have pursued experimental and computational approaches to elucidate the elementary steps that effect turnover, the catalyst speciation and resting state, and the enantiodetermining transition state. We report herein a holistic kinetic and mechanistic analysis of the KR and DKR dehydrogenative Si–O coupling (Scheme 1D).

## Results and Discussion

### 1 Initial Kinetic Investigations

We began by studying the reaction of (*S*)-1-(4-fluorophenyl)­ethanol, (*S*)-**1a**, and tri-*n*-butylhydrosilane, (*n*Bu)_3_SiH, in C_6_D_6_ (Figure 1). Reactions were monitored by in situ ^1^H and ^19^F­{^1^H} NMR spectroscopy, with 4-fluoroanisole as dual internal standard.^18^ To ensure homogeneous conditions and consistent catalyst concentrations across experiments, reactions were initiated with solutions of preformed L*CuO*t*Bu (L* = (*R*,*R*)-Ph-BPE, see Supporting Information Section 2.6). Styrene was employed as a sacrificial alkene, i.e., to undergo hydrocupration by L*CuH, forming L*CuCH­(Me)­Ph and thus suppress the formation of H_2_, without affecting initial reaction rates (see Supporting Information Section 3.1). Control experiments revealed that neither a change of solvent from toluene to C_6_D_6_ nor the introduction of styrene had a significant influence on the enantioselectivity.

**Figure 1:**
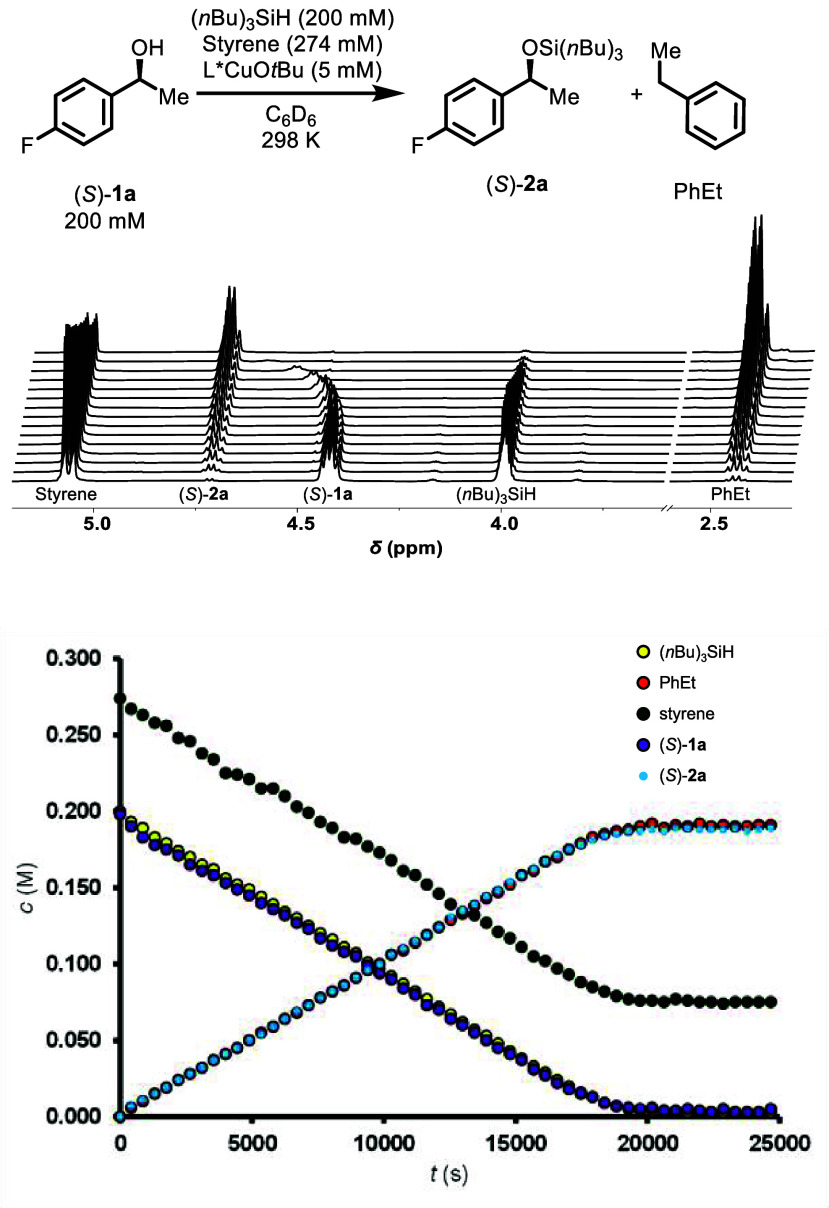
Selected spectra (2.3–2.6 ppm and 3.6–5.2 ppm) and temporal concentration profile from in situ ^1^H NMR reaction monitoring data of the L*CuO*t*Bu-catalyzed dehydrogenative coupling of (*S*)-1-(4-fluorophenyl)­ethanol ((*S*)-**1a**, 200 mM, δ_H_ = 4.40 ppm prior to initiation), and (*n*Bu)_3_SiH (200 mM, δ_H_ = 4.00 ppm) in the presence of styrene (274 mM, δ_H_ = 5.07 ppm) in C_6_D_6_ at 298 K. The products silyl ether ((*S*)-**2a**, δ_H_ = 4.72 ppm) and ethylbenzene (δ_H_ = 2.45 ppm) are shown, see Supporting Information Section 3.1 for details.

Material balances were consistent throughout reactions, and no significant intermediates or side products were detected during reaction monitoring, other than small quantities of H_2_, which were generated when [(*S*)-**1a**]_0_ > [styrene]_0_ (see Supporting Information Figure S8, Entry 9). Thus, equimolar alcohol, hydrosilane, and styrene are consumed, and converted to equimolar silyl ether (*S*)-**2a** and ethylbenzene (Figure 1). During the reaction monitoring, the ^1^H NMR signals of (*S*)-**1a** progressively shift downfield, as [(*S*)-**1a**]_*t*_ decreases (Figure 1) and the ^19^F­{^1^H} NMR signal shifts upfield. Both effects are evident in the spectra immediately after initiation of the reaction with L*CuO*t*Bu (0.005 M, 2.5 mol %) and are indicative of rapid exchange of (*S*)-**1a** with one or more copper species, resulting in time-averaged, concentration-weighted chemical shifts. In contrast, the ^1^H and ^19^F­{^1^H} NMR signals of the other reactants and products were invariant throughout (see Supporting Information Section 3.1).

Systematic variations in [(*n*Bu)_3_SiH]_0_ resulted in directly proportional changes in reaction rates. Conversely, variations in [(*S*)-**1a**]_0_ resulted in inversely proportional changes in the reaction rate, indicative that the alcohol substrate is an inhibitor. The styrene concentration has no significant affect on the reaction rate if it is present in excess, consistent with rapid styrene hydrocupration by a copper hydride species (see Supporting Information Section 3.1).^19^ Reaction rates scaled linearly with [Cu]_0_, as did product *ee* with ligand *ee* (no nonlinear effect, see Supporting Information Section 5.1 for details). A single, fixed-nuclearity Cu catalyst is the simplest explanation for the concurrence of both observations, although mixed aggregation states of Cu which coincidentally yield first-order kinetics and a linear *ee* response cannot be excluded.^20^

The above features result in the temporal concentration profile for the evolving silyl ether, (*S*)-**2a**, being dependent on the initial mole ratio of (*S*)-**1a**/(*n*Bu)_3_SiH (Figure 2). When the ratio is close to unity (i.e., [(*S*)-**1a**]_0_/[(*n*Bu)_3_SiH]_0_ ≈ 1) approximately pseudozero-order evolutions are observed (Figure 1 and Figure 2, III) until [(*S*)-**1a**]_*t*_ and [(*n*Bu)_3_SiH]_*t*_ are substantially depleted and begin to approach the catalyst concentration, [Cu]_*t*_, which is identical to its initial value, [Cu]_0_, if no deactivation process is present (see for discussion). In contrast, there is significant curvature in the product evolution profile when either (*n*Bu)_3_SiH or (*S*)-**1a** is in excess over the other. Thus, when [(*S*)-**1a**]_0_/[(*n*Bu)_3_SiH]_0_ > 1, the rate progressively decreases (Figure 2, IV–VI). Conversely, when [(*S*)-**1a**]_0_/[(*n*Bu)_3_SiH]_0_ < 1, the rate progressively increases (Figure 2, I and II). Overall, the kinetics for the reaction of (*S*)-**1a** are consistent with the steady-state rate approximation shown in eq 1 (see Figure 2 for reaction scheme), where *k*_rxn_ is an empirical turnover coefficient, and *K*_i_ is an empirical inhibition constant.^21^ The turnover rate, *v*_*s*_, approaches a pseudozero-order rate constant, *k*_obs_, when [(*S*)-**1a**]_*t*_/[(*n*Bu)_3_SiH]_*t*_ ≈ 1, as shown in eq 2 (see Supporting Information Section 8.1 for details).

1 $$\nu_{S}\approx \frac{{k_{\mathrm{rxn}}[\left(n{\mathrm{Bu})}_{3}\mathrm{SiH}\right]}_{t}{\left[\mathrm{Cu}\right]}_{t}}{1+K_{i}[{\mathrm{(S)‐}1a]}_{t}}$$

2 $$\nu_{S}\approx \frac{{k_{\mathrm{obs}}[\left(n{\mathrm{Bu})}_{3}\mathrm{SiH}\right]}_{t}}{[{\mathrm{(S)‐}1a]}_{t}}\mathrm{; when}\;K_{i}[{\mathrm{(S)‐}1a]}_{t}\gg 1$$

**Figure 2:**
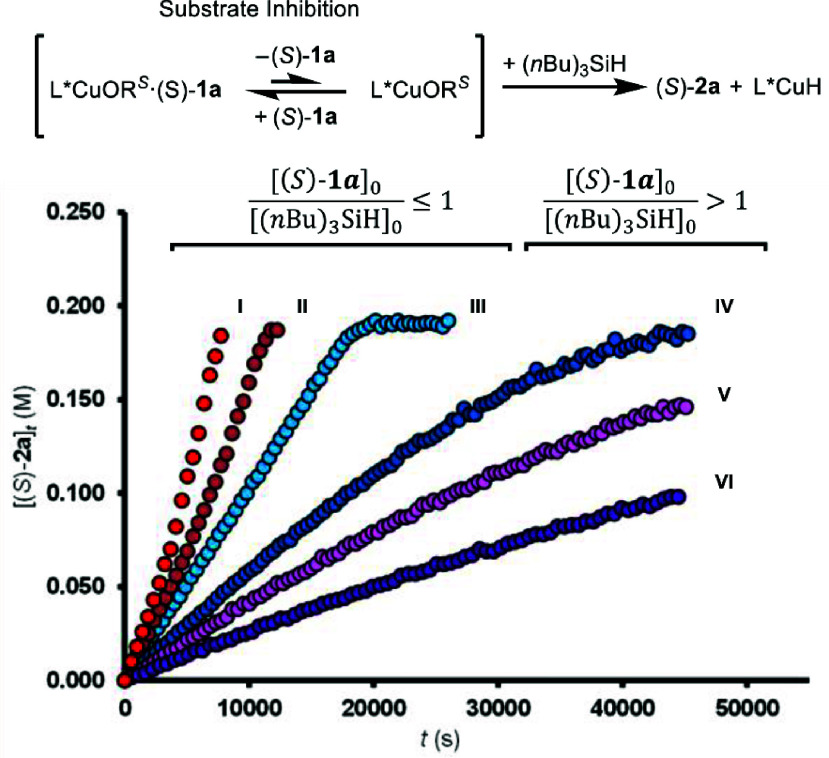
Temporal concentration profiles of (*S*)-**2a** generation under varied initial conditions: [Cu]_0_ = 0.005 M (I–VI). [styrene]_0_ = 0.27 M (I–VI). [(*S*)-**1a**]_0_ = 0.2 M (I–III) [(*S*)-**1a**]_0_ = 0.32 M (IV), 0.41 M (V), 0.65 M (VI), [(*n*Bu)_3_SiH]_0_ = 0.4 M (I), 0.27 M (II), 0.2 M (III–VI).

### 2 Mismatched, Racemic, and Pseudoracemic Alcohol 1a

The slower-reacting enantiomer (*R*)-**1a** undergoes dehydrogenative coupling with kinetics analogous to those of (*S*)-**1a** (eq 1), and *v*_0,*S*_/*v*_0,*R*_ = 4.3 under pseudozero-order conditions (Figure 3). In contrast to the reactions of the isolated *S*- and *R*-enantiomers, racemic **1a** undergoes dehydrogenative coupling with a nonlinear temporal concentration profile under conditions where the reactant ratio is unity, i.e., [**1a**]_0_/[(*n*Bu)_3_SiH]_0_ ≈ 1 (Figure 3, I). Analysis of the ^1^H NMR signals of the methine protons of (*S*)-**1a** and (*R*)-**1a** revealed a significant differential in their progressive downfield shifts (Δδ_*S*_ > Δδ_*R*_). Furthermore, an analogous upfield shift of the respective ^19^F­{^1^H} NMR signals was observed (see Supporting Information Section 3.4). Evidence that the observed shift dynamics of (*S*)-**1a** result from interactions between L*CuOR^*S*^ and (*S*)-**1a** was obtained from a mixture of L*, mesityl copper (MesCu), and (*S*)-**1a** assembled in situ and subjected to NMR spectroscopic analysis (see Supporting Information Section 5.3 for details). These effects are consistent with a significant difference in the inhibition association constants, with the overall faster reacting enantiomer (*S*)-**1a** rapidly and reversibly binding more strongly to L*CuOR than (*R*)-**1a**.

**Figure 3:**
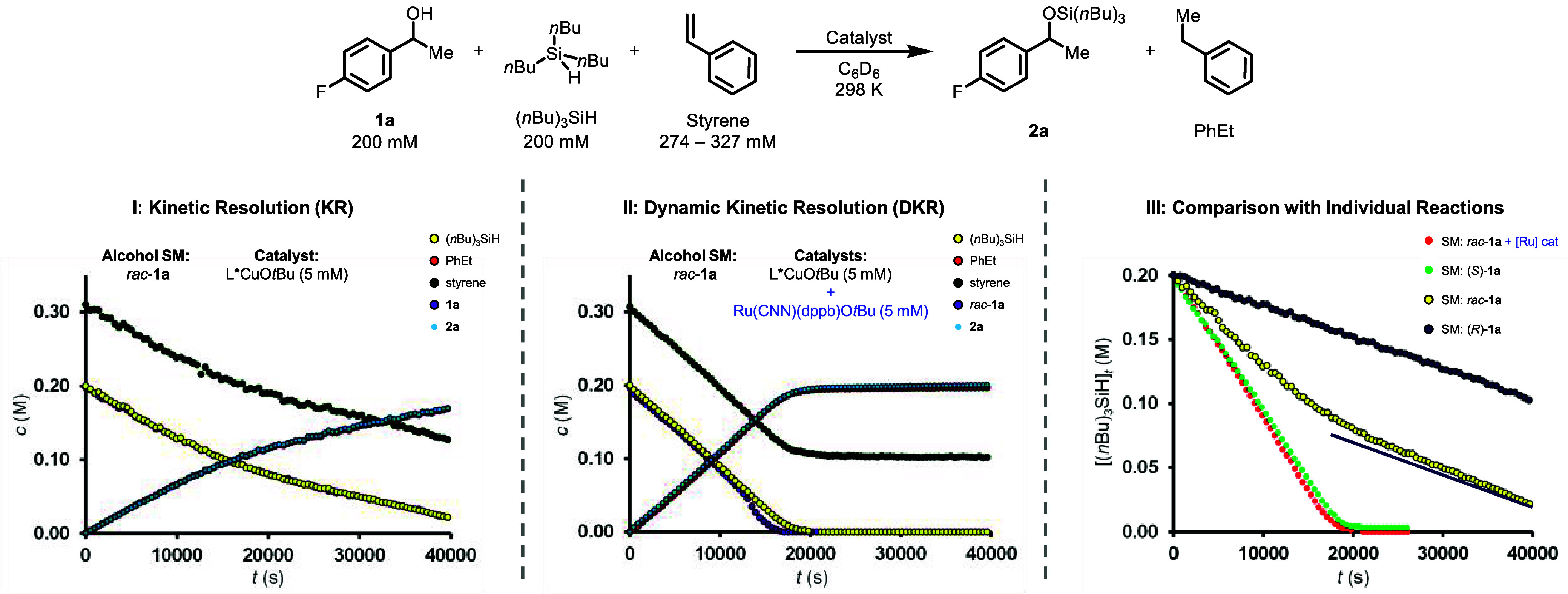
Dehydrogenative coupling of *rac*-**1a** (200 mM), (*n*Bu)_3_SiH (200 mM), styrene (274–327 mM, see Supporting Information for details of individual reactions), and L*CuO*t*Bu (5 mM) in C_6_D_6_ at 298 K (KR, I, SI Section 3.4), and a reaction under identical conditions with the addition of a racemization catalyst, Ru­(CNN)­(dppb)­O*t*Bu (prepared in situ from Ru­(CNN)­(dppb)­Cl^14j,22^ and NaO*t*Bu, 5 mM, DKR, II, SI Section 3.5). A comparison of the (*n*Bu)_3_SiH consumption of I, II, and dehydrogenative couplings of enantiopure (*R*)-**1a** (SI Figure S9, Entry 18), and enantiopure (*S*)-**1a** (Figure 1) is shown (III). A blue line is added to the reaction of *rac*-**1a** for comparison with the reaction of (*R*)-**1a** as a visual aid.

Subsequently, the behavior of the system under DKR conditions, facilitated by Ru­(CNN)­(dppb)­O*t*Bu^14j,22^ cocatalysis, was investigated. Separate time-averaged ^19^F­{^1^H} NMR signals of identical intensities were detected for (*S*)-**1a** and (*R*)-**1a** throughout the reaction, indicative that alcohol **1a** is dynamically racemic throughout the dehydrogenative coupling. Identical conclusions are drawn by analysis of the pseudoquintet corresponding to the methine signals of (*S*)-**1a** and (*R*)-**1a** during ^1^H NMR reaction monitoring. Racemization is thus rapid in comparison to the concurrent dehydrogenative coupling process, as is ideal for a DKR process (*k*_rac_ ≥ 400·*k*_obs_, see Supporting Information Section 8.3 for discussion). Moreover, the racemization restores the pseudozero-order kinetic regime (Figure 3, II) at a reaction rate that is similar to that of the dehydrogenative coupling of (*S*)-**1a** (Figure 3, III). The nonlinear kinetic regime in the absence of Ru cocatalysis (Figure 3, I) is thus the result of a progressive change in (*S*)-**1a**/(*R*)-**1a** ratio, as is expected in a KR process, whereas the (*S*)-**1a**/(*R*)-**1a** ratio is constant under DKR conditions.

As the reactive species L*CuOR^*S*^ and L*CuOR^*R*^ rapidly equilibrate, formation of (*S*)-**2a** is irreversible, and L*CuOR complexes are in low concentrations relative to (*n*Bu)_3_SiH, the relative rate of the substrate-committing elementary reactions, *k*_S_/*k*_R_, can be estimated by competition experiments. The pseudoenantiomeric pairs, (*S*)-**1a-C**_**D**_/(*R*)-**1a** (Figure 4, I) and (*S*)-**1a**/(*R*)-**1a**-**C**_**D**_ (Figure 4, II), facilitate the direct ^1^H NMR spectroscopic resolution and quantification of the stereochemical ratios in the substrates and products. The changes in pseudoenantiomeric substrate ratio, *R*_A_, with fractional conversion, *F*_L_, were fitted to first-order competition models to give (*k*_S_·*KIE*_H/D(*S*)_)/*k*_R_ = 7.2 and *k*_S_/(*k*_R_·*KIE*_H/D(*R*)_) = 7.6 (Figure 4). If the small inverse secondary kinetic isotope effect (KIE), *k*_H_/*k*_D_ ≈ 0.97, is enantiomer-independent, then the kinetic resolution factor, *k*_S_/*k*_R_, is 7.4 (see Supporting Information Section 4.5).^23^ The rate-differential between (*S*)-**1a** and (*R*)-**1a** in competition is thus greater than that in independent reactions, *v*_0,*S*_/*v*_0,*R*_ = 4.3. This is consistent with an inhibition constant for (*S*)-**1a**, *K*_i,*S*_, which is greater than *K*_i,*R*_ by a factor of 7.4/4.3 = 1.7, despite (*S*)-**1a** undergoing faster overall transformation to (S)-**2a** than (*R*)-**1a** to (*R*)-**2a**.

**Figure 4:**
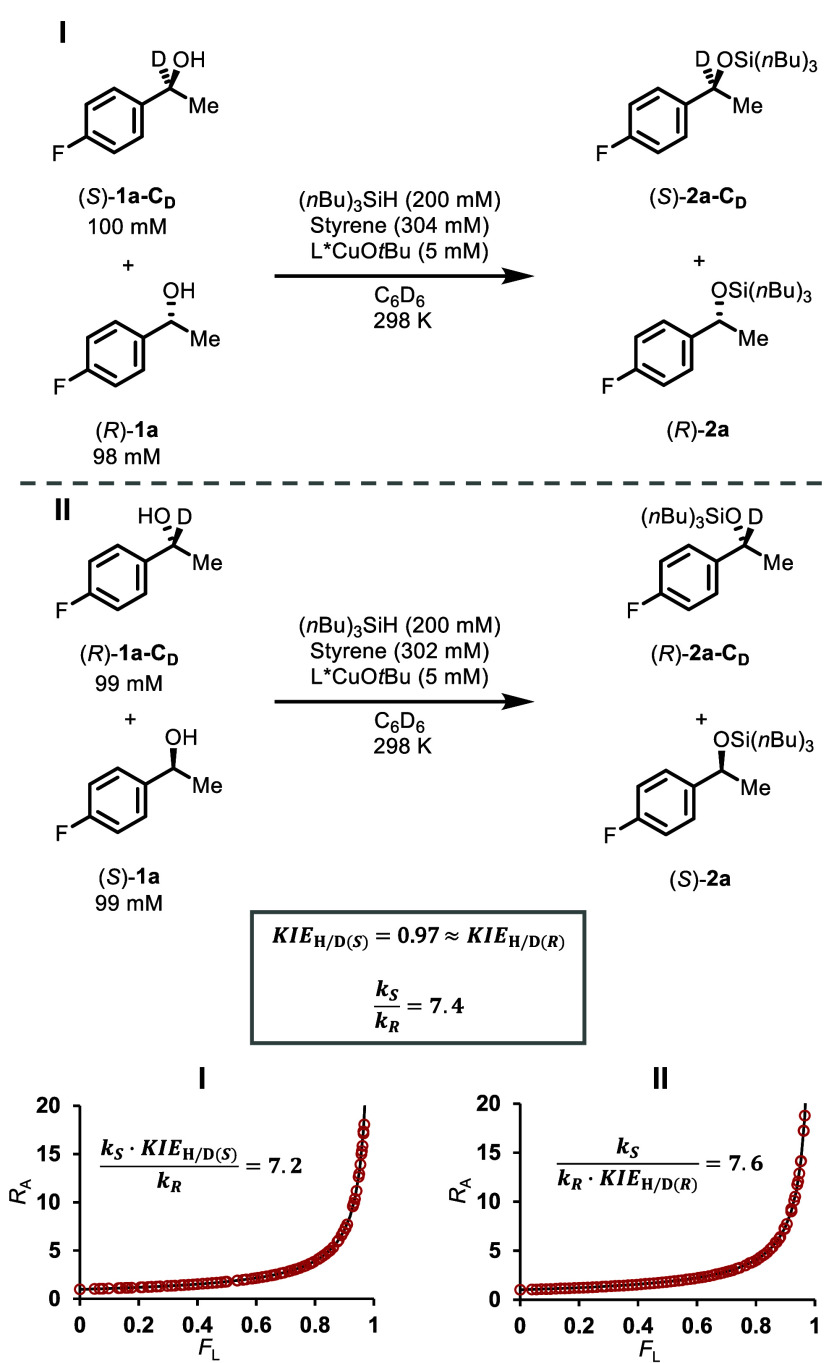
Determination of the relative reactivity of **1a** enantiomers via intermolecular competitions between (*S*)-**1a-C**_**D**_/(*R*)-**1a** (I), and (*S*)-**1a**/(*R*)-**1a**-**C**_**D**_ (II), see Supporting Information Section 4.2 for detailed discussion of the data fitting to a Bigeleisen–Wolfsberg equation.

### 3 Activation Parameters and Primary Si–H and O–H Kinetic Isotope Effects

The temperature dependence of the rates of the Cu-catalyzed reaction between (*S*)-**1a** and (*n*Bu)_3_SiH in the 288–308 K temperature range was determined under pseudozero-order conditions. Eyring analysis of the empirical rate coefficient, *k*_obs_ = *v*_0_/[Cu]_0_, gave approximate activation parameters (Figure 5, I, see Supporting Information Section 3.6). The enthalpy of activation (ΔH^‡^ = 14 kcal·mol^–1^), entropy of activation (ΔS^‡^ = −23 cal·K^–1^·mol^–1^), and free energy of activation (ΔG^‡^ = 21 kcal·mol^–1^ at 298 K) are consistent with a highly ordered transition state with loss of translational degrees of freedom, as would be expected for σ-bond metathesis (Figure 7) in an apolar aprotic solvent.^24,25^

**Figure 5:**
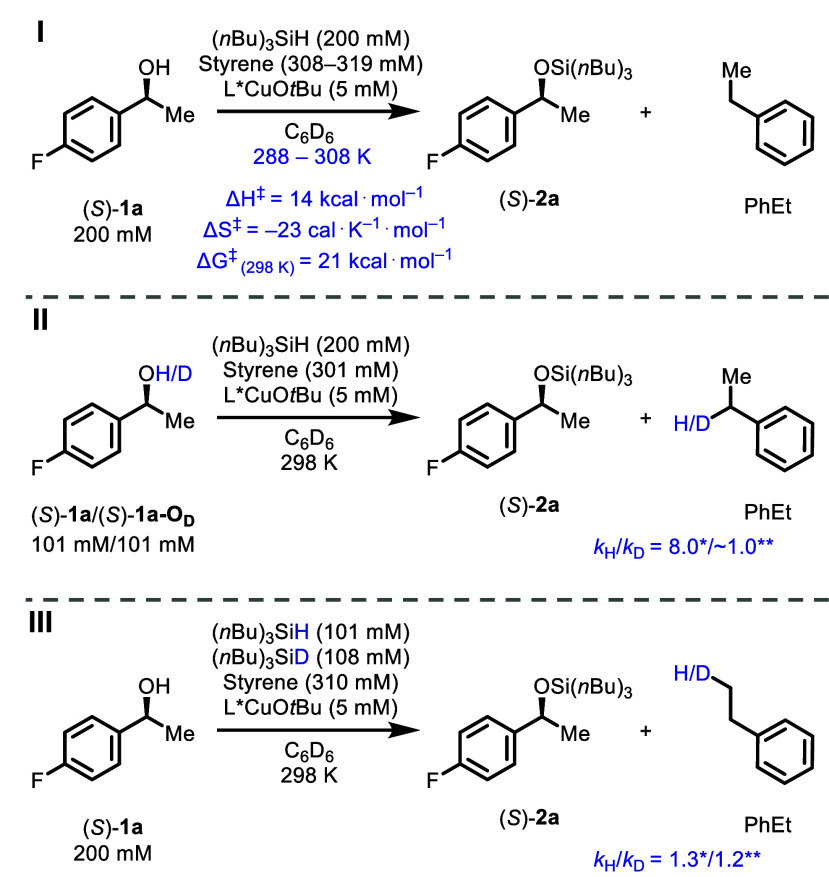
Summary of temperature and isotope effects: Eyring activation parameters (I), H/D isotope effect of hydroxy group measured via intermolecular competition (II), and H/D isotope effects of hydrosilane measured via intermolecular competition and independent kinetic measurements (III). *k*_H_/*k*_D_ values reported with a single asterisk (*) represent intermolecular competition, those reported with double asterisks (**) represent independent kinetic measurements. Concentrations in (II) and (III) are given for intermolecular competition experiments. The independent H/D KIE value for (*S*)-**1a**-**O**_**D**_ is represented as ∼1 at the same rate as (*S*)-**1a** at initial reaction stages, see Supporting Information Section 4.6 for details.

To further probe the process, the primary KIE for the Cu-catalyzed reaction of (*S*)-**1a** with (*n*Bu)_3_SiD versus (*n*Bu)_3_SiH was determined by intermolecular competition (*k*_H_/*k*_D_ = 1.3) and from independent reactions (*k*_H_/*k*_D_ = 1.2). The small normal primary KIE values are again consistent with a σ-bond metathesis process and agree with theoretical calculations (Figure 9). Deuterium incorporation in the ethylbenzene coproduct occurred exclusively at the terminus, forming PhCH_2_CH_2_D, consistent with prior reports on the reactions of Cu–D complexes with styrene (Figure 5, III).^13a^

Reaction of the O-deuteriatedalcohol (*S*)-**1a-O**_**D**_ under the standard reaction conditions shown in Figure 1 proceeded with the same initial rates as a reaction of (*S*)-**1a** (*v*_0,OH_/*v*_0,OD_ = 1), but departed from the pseudozero-order kinetic regime at an earlier stage than that of (*S*)-**1a** (see Supporting Information Section 4.6). This results from a large primary KIE attending the reaction of (*S*)-**1a-O**_**H/D**_ with L*CuCH­(Me)­Ph, with the change in kinetic regime which arises from the partial accumulation of the latter as the alcohol is depleted. The primary KIE for the deprotonation step was determined as *k*_H_/*k*_D_ = 8.0 through intramolecular competition (Figure 5, II), in agreement with theoretical calculations (Figure 9).

### 4 Alkoxide and Alcohol Substituent Effects

Further details on the elementary reaction of L*CuOR^*S*^ with (*n*Bu)_3_SiH were obtained from linear free energy relationships (LFERs), determined using *p*-substituted copper alkoxides generated via equilibration with the corresponding alcohols, (*S*)-**1a** (X = F), (*S*)-**1b** (X = H), (*S*)-**1c** (X = Me), (*S*)-**1d** (X = OMe), and (*S*)-**1e** (X = CF_3_). Copper alkoxides bearing an electron-withdrawing aromatic substituent react preferentially in intermolecular competition experiments (Figure 6, I). However, the difference in reactivity is small, with ρ = +0.6 using standard Hammett substituent parameters.^26^

**Figure 6:**
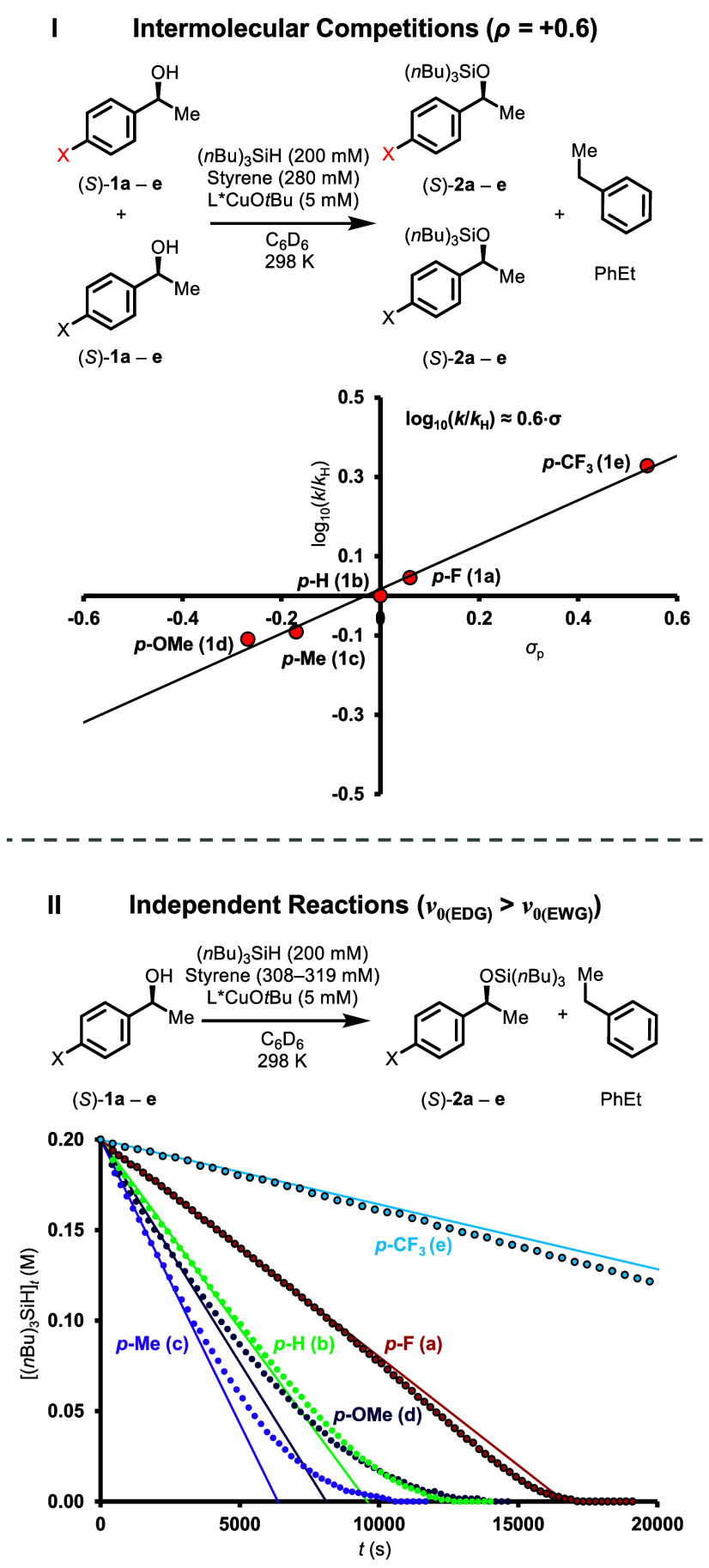
Substituent effects of *p*- substituted derivatives of (*S*)-**1**. Hammett correlation of intermolecular competition experiments between pairs of (*S*)-**1** derivatives (I), and independent reactions of alcohols (*S*)-**1** (II, *p*-substituent X: **a** = F, **b** = H, **c** = Me, **d** = OMe, **e** = CF_3_). Experimental data (circles) and a linear extrapolation from the initial slope of the reaction (∼10% conversion) are shown in (II) for the independent reactions.

In contrast to the competition experiments, independent reactions of the alcohols (*S*)-**1** undergo faster turnover when they have electron donating aromatic substituents (Figure 6, II, see Supporting Information Section 4.4 for detailed discussion). The faster reacting systems (X = H, Me, OMe) depart at an earlier stage from the pseudozero-order kinetic regime. Conversely, the reaction of (*S*)-**1e** (X = CF_3_) undergoes acceleration as (*S*)-**1e** is consumed. The data indicate that, under conditions where [(*S*)-**1**]_0_/[(*n*Bu)_3_SiH]_0_ ≈ 1, the concentration range enabling pseudozero-order kinetics is alcohol-dependent. These features likely result from competing interactions, as while alcohols with electron-withdrawing group (EWG) substituents form L*CuOR complexes which are more reactive toward (*n*Bu)_3_SiH, they also inhibit turnover by more strongly favoring the formation of off-cycle resting state complexes L*CuOR·ROH. A resting state in which ROH acts as an H-bond donor to a Cu-alkoxide oxygen acceptor (Figure 7) is consistent with the observed electronic effects and kinetic behavior. The free energy difference for inhibition, **ΔG**_**Inhib**_, increases when X = EWG, while the relative barrier for turnover, **ΔG** ^**‡**^_**TS**_, decreases. The opposite effects are induced when X = EDG. Thus, in intermolecular competition where all preceding equilibria are rapid, the relative rates are determined by **Δ­(ΔG** ^**‡**^_**TS**_ **)** (Figure 6, I), whereas relative reaction rates for the substrates in isolation (Figure 6, II) are governed by **Δ­(ΔG**_**Inhib**_ + **ΔG** ^**‡**^_**TS**_ **)**. This model rationalizes the alcohol-dependent concentration windows within which pseudozero-order kinetic regimes persist, consistent with differences in **ΔG**_**Inhib**_ across alcohols. The acceleration (X = CF_3_, F), and deceleration (X = OMe, Me, H) observed with alcohol conversion reflect that **ΔG**_**Inhib**_ is more sensitive to substituent X than **ΔG** ^**‡**^_**TS**_, in agreement with Hammett and Swain-Lupton analyses (see Supporting Information Section 4.4 for detailed discussion).

**Figure 7:**
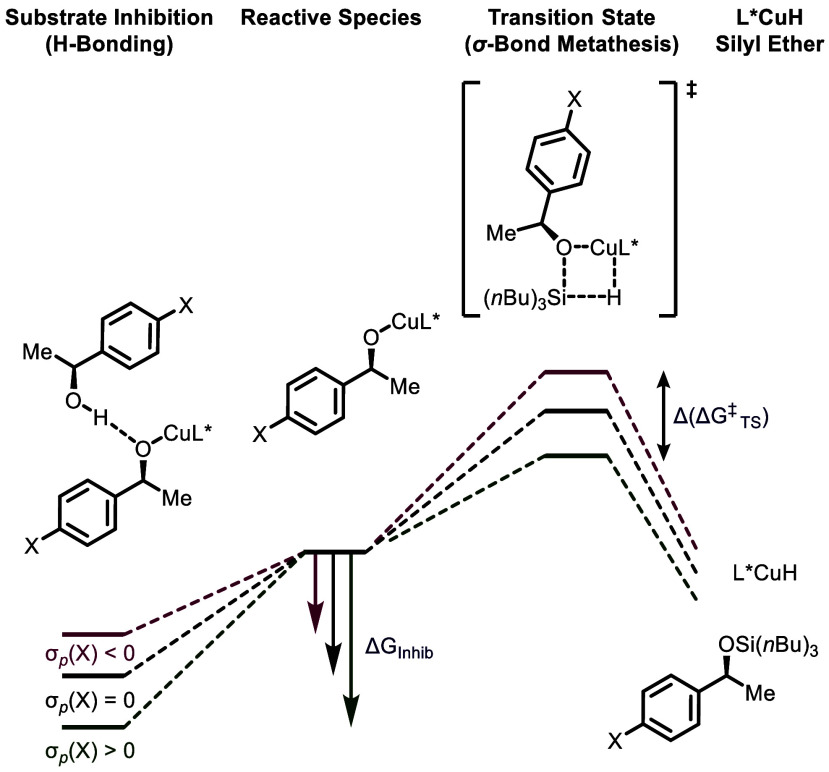
Schematic potential energy surface of dehydrogenative couplings of *p*-substituted (*S*)-**1** derivatives. Proposed resting and transition states consistent with the obtained experimental data are shown.

### 5 Kinetic Model

A minimal kinetic model that accounts for all of the experimental features of the Cu-catalyzed reaction of (*S*)-**1a** and (*n*Bu)_3_SiH in the presence of styrene was constructed using a local parameter solution from simultaneous least-squares fits across multiple data sets (Figure 8).^27^ The model assumes that the precatalyst, L*CuO*t*Bu, is rapidly and irreversibly converted to L*CuOR^*S*^ (*k*_act_, see Supporting Information Section 8.3 for discussion), consistent with generated *t*BuOH being at low concentrations and likely a poor inhibitor and substrate (see Section 4). Additionally, the model assumes no off-cycle higher-order aggregates of (*S*)-**1a** in order to avoid a model prone to overfitting.^28^ Equilibration of L*CuOR^*S*^ with the off-cycle hydrogen-bonded adduct [L*CuOR^*S*^·(*S*)-**1a**] (*K*_i_) and of its higher homologue [L*CuOR^*S*^·(*S*)-**1a**·(*S*)-**1a**] (*K*’_i_) accounts for the inhibition by (*S*)-**1a**, with the latter included to better account for increasing inhibition at high (*S*)-**1a** concentrations. The on-cycle copper alkoxide L*CuOR^*S*^ reacts with (*n*Bu)_3_SiH via Si–H/Cu–O σ-bond metathesis to generate (*S*)-**2a** and L*CuH (*k*_1_). There are two pathways for L*CuOR^*S*^ regeneration from L*CuH: hydrocupration of styrene (*k*_2_) followed by deprotonation of (*S*)-**1a** by L*CuCH­(Me)­Ph (*k*_3_), or direct deprotonation of (*S*)-**1a** by L*CuH (*k*_4_). Furthermore, a pathway for L*CuH deactivation through dimerization was included (*k*_dim_, see Supporting Information Section 3.3 for discussion).^29^

**Figure 8:**
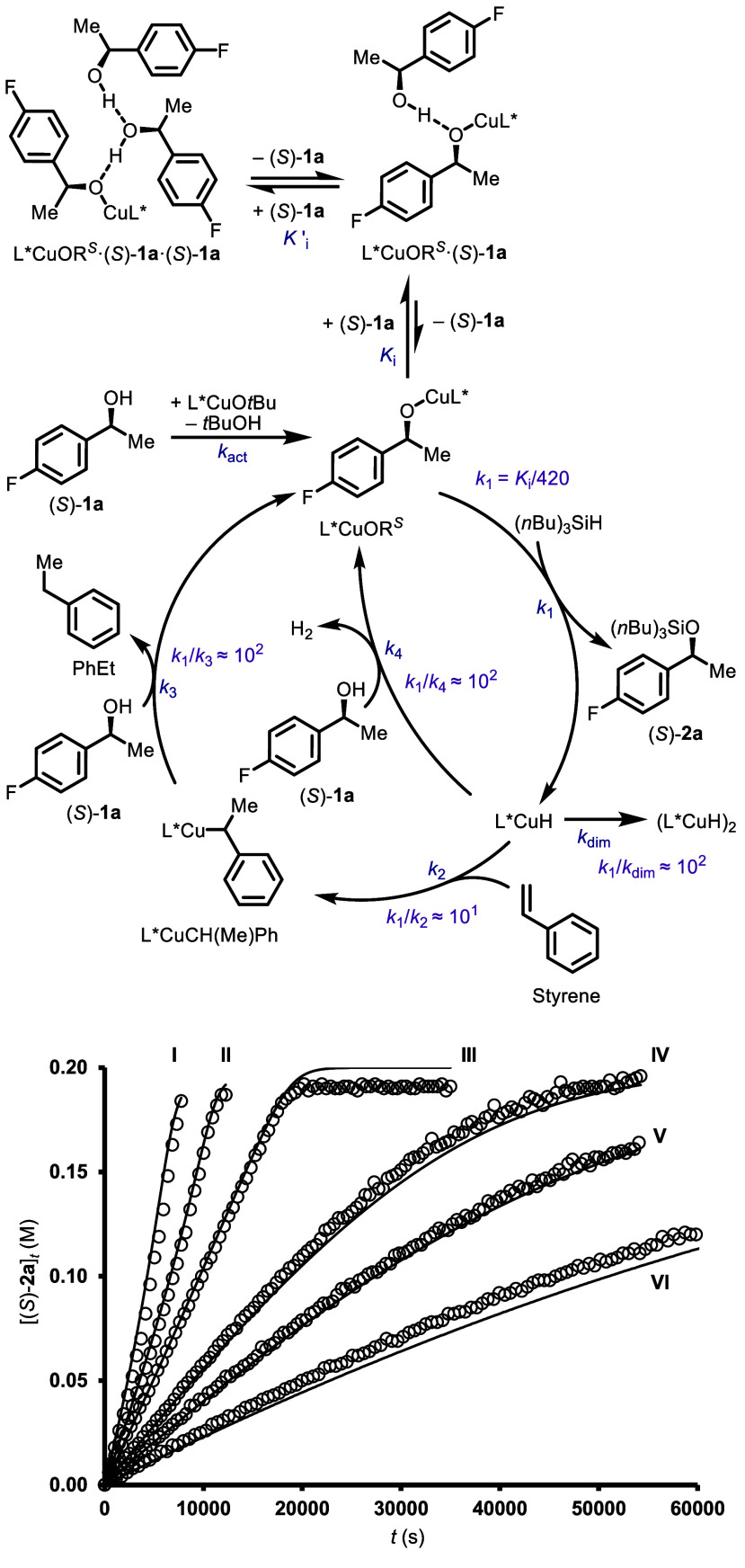
Kinetic model for reactions of (*S*)-**1a** (Top) and comparison of simulation (lines) with experimental data (open circles, ^1^H NMR spectroscopy, [(*S*)-**2a**]_*t*_, initial conditions: [Cu]_0_ = 0.005 M (I–VI). [styrene]_0_ = 0.27 M (I–VI). [(*S*)-**1a**]_0_ = 0.2 M (I–III), 0.32 M (IV), 0.41 M (V), 0.65 M (VI), [(*n*Bu)_3_SiH]_0_ = 0.4 M (I), 0.27 M (II), 0.2 M (III–VI) (Bottom). For fitted kinetic parameters see Supporting Information Section 8.1.

The model satisfactorily correlates with experimental data across a broad range of conditions, including the acceleration of reactions when [(*S*)-**1a**]_0_/[(*n*Bu)_3_SiH]_0_ < 1, and deceleration when [(*S*)-**1a**]_0_/[(*n*Bu)_3_SiH]_0_ > 1 (Figure 8). Although *k*_1_ is at least an order of magnitude larger than the other major on-cycle rate coefficients, *k*_2_, *k*_3_, *k*_4_, and *k*_dim_, under most conditions the dominant resting state is off-cycle because *K*_i_/*k*_1_ ≈ 4 × 10^2^ s. The latter accounts for the earlier departure from the pseudozero-order kinetic regime with (*S*)-**1a**-**O**_**D**_ versus (*S*)-**1a**, and changes in catalyst speciation detected by ^1^H and ^31^P­{^1^H} NMR spectroscopy when [(*S*)-**1a**]_*t*_ approaches [Cu]_0_. Furthermore, the model is fully applicable to (*R*)-**1a** based on the obtained (*R*)-**1a** data (see Supporting Information Section 8.1 for discussion).

### 6 Computational Investigations

DFT calculations were employed to elucidate the observed experimental trends and verify the feasibility of proposed intermediates (see Figure 9, A for full catalytic cycle and Supporting Information Section 7.1 for computational details).^30^ The calculations indicate that all of the H-bonded adducts, [L*CuOR·**1a**], are more stable than the parent monomeric copper alkoxides, and are therefore the major resting state under the reaction conditions. Coordination of the alcohol oxygen atom to the Cu center was found to be ≥ 3 kcal·mol^–1^ less favorable than the proposed H-bonding inhibition mode (see Supporting Information Section 7.2). The homologated inhibition adduct of (*S*)-**1a**, [L*CuOR^*S*^·(*S*)-**1a**·(*S*)-**1a**], employed in the kinetic model to account for stronger inhibition at high (*S*)**-1a** concentrations (Figure 8) was predicted to be –0.6 kcal·mol^–1^ more favorable than [L*CuOR^*S*^·(*S*)**-1a**]. While this indicates that higher-order inhibition effects through this mode are feasible, these will only be evident when (*S*)**-1a** is present at high concentrations. Dissociation of inhibited species to the reactive copper-alkoxide L*CuOR is followed by σ-bond metathesis with (*n*Bu)_3_SiH via TS-I (Δ**G** ^**‡**^_***S***_ = 16.8 kcal·mol^–1^, Δ**G** ^**‡**^_***R***_ = 17.7 kcal·mol^–1^), forming silyl ether **2a** and L*CuH.^31,32^ The L*CuH coproduct reacts with styrene via TS-II to form (*R*)-isophenethyl copper, L*Cu-(*R*)–CH­(Me)­Ph (Δ**G** ^**‡**^ = 8.4 kcal·mol^–1^), which subsequently deprotonates **1a** in TS-III (Δ**G** ^**‡**^_***S***_ = 17.6 kcal·mol^–1^, Δ**G** ^**‡**^_***R***_ = 19.6 kcal·mol^–1^), regenerating L*CuOR and irreversibly generating PhEt.

**Figure 9:**
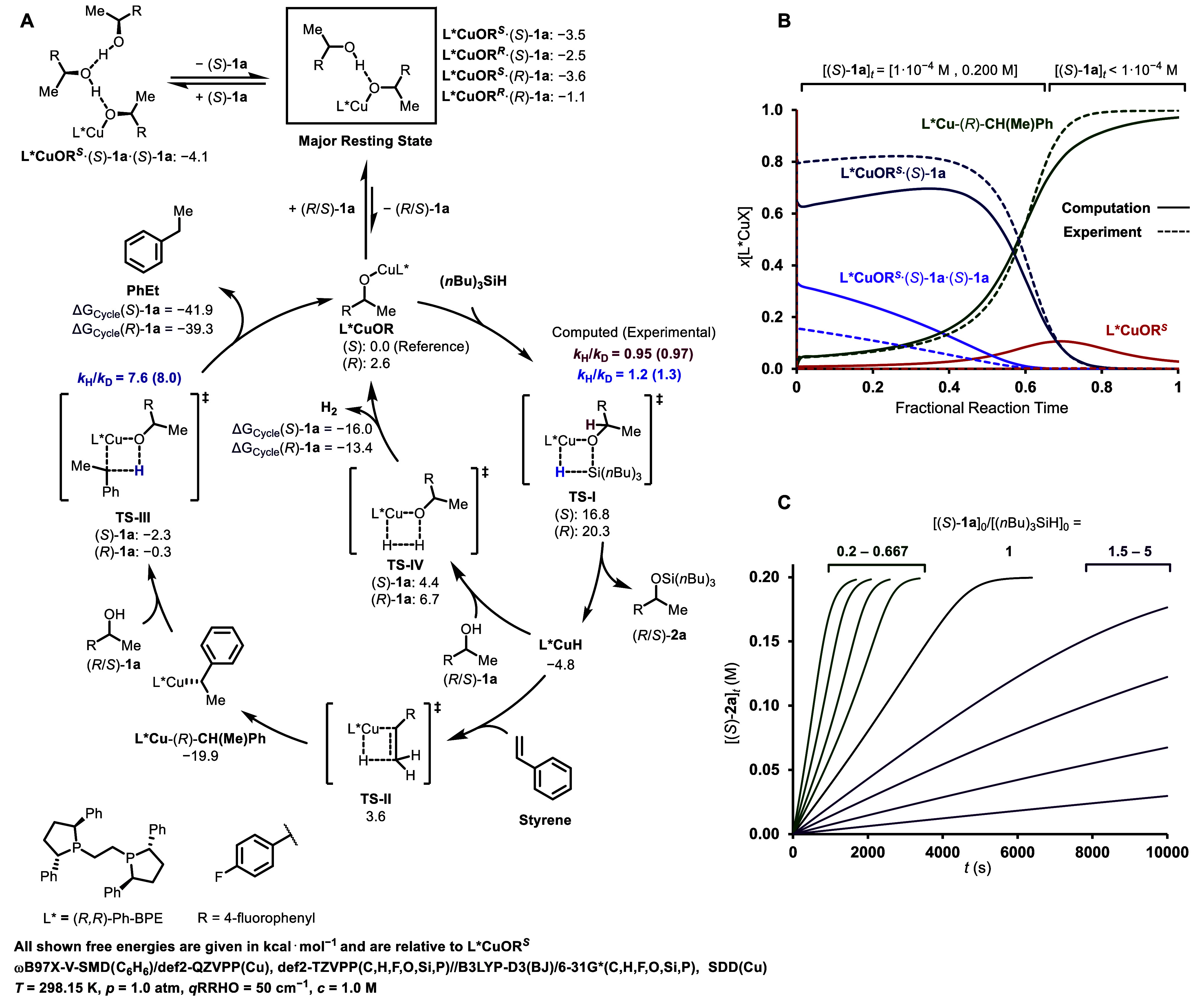
A: Computed catalytic cycle for reactions of (*S*)-**1a** and (*R*)-**1a** with (*n*Bu)_3_SiH, styrene in the presence of a L*Cu catalyst system. Values under species are relative free energies in kcal·mol^–1^ referenced to L*CuOR^*S*^. See Supporting Information Section 7.1 for details. Kinetic simulations^27^ employing the standard state computational free energies are depicted for mole fraction of catalyst species [L*CuOR^*S*^·(*S*)-**1a**], L*CuOR^*S*^, [L*CuOR^*S*^·(*S*)-**1a**·(*S*)-**1a**], and L*Cu-(*R*)-CH­(Me)­Ph during a reaction as (*S*)-**1a** is consumed (B, Initial conditions: [Cu]_0_ = 0.005 M, [styrene]_0_ = 0.274 M, [(*S*)-**1a**]_0_ = 0.200 M, [(*n*Bu)_3_SiH]_0_ = 0.200 M, data simulated from experimental kinetic parameters is shown for comparison as dashed lines (Figure 8, see Supporting Information Section 8.1 for details), and for the variation of [(*n*Bu)_3_SiH]_0_ and [(*S*)-**1a**]_0_, with [(*S*)-**1a**]_0_/[(*n*Bu)_3_SiH]_0_ = 0.2–5 (C, Initial conditions: [Cu]_0_ = 0.005 M, [styrene]_0_ = 1.0 M, [(*S*)-**1a**]_0_ = 0.200–1.0 M, [(*n*Bu)_3_SiH]_0_ = 0.200–1.0 M).

The reactions of both **1a** enantiomers have large magnitude, negative free energies of turnover (Δ**G**_**Cycle** **)**_(*S*)-**1a** = –41.9 kcal·mol^–1^, Δ**G**_**Cycle**_(*R*)-**1a** = –39.3 kcal·mol^–1^ relative to the L*CuOR^S^ reference state), in agreement with an overall irreversible reaction. Alternatively, L*CuH may react directly with either enantiomer of **1a** via TS-IV to form L*CuOR and H_2_ (Δ**G** ^**‡**^_***S***_ = 9.2 kcal·mol^–1^, Δ**G** ^**‡**^_***R***_ = 11.5 kcal·mol^–1^), and free energies of turnover calculated for this pathway are still indicative of an overall irreversible reaction (Δ**G**_**Cycle**_ *S*-**1a** = –16.0kcal·mol^–1^, Δ**G**_**Cycle**_ *R*-**1a** = –13.4 kcal·mol^–1^relative to the L*CuOR^S^ reference state).

Eyring activation parameters calculated for TS-I (from L*CuOR^*S*^·(*S*)-**1a** as the ground state, ΔH^‡^ = 16 kcal·mol^–1^, ΔS^‡^ **=** –12 cal·K^–1^·mol^–1^) as well as KIEs calculated for TS-I and TS-III (Figure 9, A) are in good agreement with the experimentally determined values (Figure 4 and Figure 5). Translation of the computed standard state free energies for species in Figure 9 into rate and equilibrium constants for the kinetic model in Figure 8 results in holistic qualitative agreement with experimental observations. These include the predicted speciation of L*CuX (Figure 9, B), and the extent and direction of curvature in the evolution of temporal concentrations (Figure 9, C). Overall, the agreement with the experimental kinetic model indicates that the manifested computational errors are mostly systematic in nature.

## Conclusion

The kinetics and mechanism of the copper-catalyzed dehydrogenative silylation of (*S*)-1-(4-fluorophenyl)­ethanol, (*S*)-**1a**, with tri-*n*-butylhydrosilane, (*n*Bu)_3_SiH (Figure 1), using styrene to undergo hydrocupration and avoid H_2_ generation, have been investigated using ^1^H and ^19^F­{^1^H} NMR spectroscopy reaction monitoring, isotopic labeling, and computation. Initial experimental evidence showed styrene insertion into a copper hydride intermediate to be rapid and [styrene]_0_ not to affect the reaction kinetics when used in excess. Increasing values of [(*n*Bu)_3_SiH]_0_ and [Cu]_0_ showed a positive correlation with reaction rate. Conversely, the alcohol substrate was found to be an inhibitor, with the observed reaction rate correlating negatively with [(*S*)-**1a**]_0_. Consequently, the substrate ratio [(*S*)**-1a**]_0_/[(*n*Bu)_3_SiH]_0_ governs the observed macrokinetic regime, with values close to unity resulting in pseudozero-order temporal concentration profiles (Figure 2).

A similar kinetic regime was observed with the slower reacting enantiomer (*R*)-**1a** (see Supporting Information Figure S9, Entry 16), but not with racemic **1a** (Figure 3, I), which elicited biphasic kinetics that tended toward linear after full consumption of (*S*)-**1a** (see Supporting Information Section 3.4). This was interpreted as evidence that the speciation of the catalyst resting state is enantiomer dependent in the reactions of **1a**. Addition of a racemization catalyst, Ru­(CNN)­(dppb)­O*t*Bu, resulted in a temporal concentration profile of *rac*-**1a** that was virtually identical to that of (*S*)-**1a** (Figure 3, II and III). This is consistent with the hypothesis that constant enantiomeric ratio results in constant speciation of the catalyst resting state, and that the catalyst resting state in the DKR experiment is identical or of comparable energy to that of reactions involving only (*S*)-**1a** (see Supporting Information Section 3.5).

The relative rate of the substrate-committing elementary reactions of the two enantiomers of the alcohol was determined as *k*_S_/*k*_R_ = 7.4 using pseudoracemic mixtures of isotopically labeled **1a** enantiomers (Figure 4, I and II) and correcting for the kinetic isotope effect resulting from deuteriation at the benzylic carbon. The relative rate was also estimated directly by ^19^F­{^1^H} NMR reaction monitoring of the reaction of *rac*-**1a** (see Supporting Information Sections 3.4 and 4.5). It was also observed that an increased ligand loading did not affect the overall kinetics or enantioselectivity of the process (see Supporting Information Section 4.5). The *k*_S_/*k*_R_ value significantly differed from the macrokinetic value obtained from independent reactions of enantiopure (*R*)-**1a** and (*S*)-**1a** (*v*_0,*S*_/*v*_0,*R*_ = 4.3). Unlike macrokinetic rates *v*_0_, values of *k*_S_/*k*_R_ do not depend on the catalyst resting state, but only on relative product-committing transition state energies. This difference provides compelling evidence that the catalyst resting state speciation is a function of the evolving enantiomeric composition of **1a**. The effects of changes in temperature and isotopic substitution at reactive sites were investigated over a temperature range of 288–308 K (Figure 5, I), yielding the enthalpy (ΔH^‡^ = 14 kcal·mol^–1^) and entropy of activation (ΔS^‡^ = −23 cal·K^–1^·mol^–1^). Additionally, H/D KIEs were observed for (*S*)-**1a**/(*S*)-**1a**-**O**_**D**_ (*k*_H_/*k*_D_ = 8.0, Figure 5, II), and (*n*Bu)_3_SiH/(*n*Bu)_3_SiD (*k*_H_/*k*_D_ = 1.3, Figure 5, III) in intermolecular competition experiments. The activation parameters and Si–H/D KIE are consistent with a highly ordered transition state with loss of translational degrees of freedom, while the O–H/D KIE value is consistent with a strong change in vibrational properties in the transition state relative to the ground state, such as in a deprotonation step.

The reaction kinetics for (*S*)-**1** derivatives bearing other substituents than fluorine ((*S*)-**1b**–**e**) in their aromatic ring were subsequently investigated. For intermolecular competitions the electron-richer alcohols react slower (Figure 6, I). Conversely, in their independent reactions the electron-richer (*S*)-**1** alcohols generally react faster (Figure 6, II). This shows that different factors govern catalyst inhibition and substrate-committing reactivity. Modes of inhibition and reactivity that are holistically consistent with experimental observations include inhibition of the catalytically active species L*CuOR^*S*^ via H-bonding, and a substrate-committing step involving Si–H/Cu–O σ-bond metathesis (Figure 7).

A minimal kinetic model accounting for all experimental observations was developed and shown to correlate well with the experimental data (Figure 8). The reaction of **1a** was also investigated with theoretical calculations in broad agreement with experimental observations, reproducing measured H/D KIEs, the relative energies of elementary steps, and the overall enantioselectivity.^33^ Moreover, when the experimental kinetic model was applied using the energies obtained from theoretical calculations, the computed catalytic cycle is in good agreement with the predicted catalyst speciation (Figure 9).

These mechanistic investigations have resulted in a holistic representation of the general reactivity in systems containing secondary benzylic alcohols, (*n*Bu)_3_SiH, styrene, and L*CuO*t*Bu in benzene, and the general features identified are expected to be applicable across various secondary alcohols with comparable electronics and across hydrosilanes of comparable hydricity. While substrate inhibition by derivatives of **1** undergoing kinetic resolution limits the turnover rates, this can be attenuated by slow addition of **1** to stoichiometric quantities of (*n*Bu)_3_SiH in the presence of a racemization cocatalyst, e.g., Ru­(CNN)­(dppb)­O*t*Bu. Furthermore, it was found that the absence of styrene from the reaction mixture resulted in eventual stalling and potential catalyst deactivation (see Supporting Information Section 3.3) while generating stoichiometric, potentially hazardous quantities of H_2_. Overall, it is shown that a combination of a racemization catalyst (such as Ru­(CNN)­(dppb)­O*t*Bu) and styrene, or other substrates able to efficiently undergo hydrocupration at comparable rates, are unlikely to compromise the process kinetics of benzylic secondary alcohols while improving overall efficiency and safety.

## Supplementary Material





## Notes

Experimental details, reaction profiles, and characterization data, as well as computational details (PDF)
Computational geometries (XYZ)
