# Supplementary material for: Kinetics and Mechanism of Enantioselective Cu-Catalyzed Alcohol Silylation
Source: J Am Chem Soc. 2025 Nov 20;147(48):44372–83. doi: 10.1021/jacs.5c14937 (PMC12679643; doi:10.1021/jacs.5c14937)
Supplement: Supplementary file 1 [file ja5c14937_si_001.pdf]

SUPPORTING INFORMATION

Kinetics and Mechanism of Enantioselective Cu-Catalyzed Alcohol Silylation

Pedro H. Helou de Oliveira,[a,b] Jan Seliger,[a] Shoutong Rao,[c] Guy C. Lloyd-Jones,[b]* Guoqiang Wang,[c]* and Martin Oestreich[a]*

*E-mail: wangguoqiang710@nju.edu.cn; guy.lloyd-jones@ed.ac.uk; martin.oestreich@tuberlin.de

[a]Institut für Chemie, Technische Universität Berlin, 10623 Berlin, Germany; [b]School of Chemistry, University of Edinburgh, David Brewster Road, Edinburgh, EH9 3FJ, UK; [c]State Key Laboratory of Coordination Chemistry, School of Chemistry and Chemical Engineering, Nanjing University, Nanjing 210023, P.R. China.

TABLE OF CONTENTS

1. General Considerations **S4**

2. General Procedures **S6**

2.1 Reaction Monitoring Experiments (GP1) **S6**

2.2 Preparation of *rac*-**1a**–**e** alcohols (GP2) **S6**

2.3 Kinetic Resolution of *rac*-**1a**–**e** alcohols by Enzymatic Acylation (GP3) **S6**

2.4 General Procedure for the Base-Mediated Hydrolysis of Acetic Acid Esters (GP4) **S7**

2.5 General Procedure for the TBAF-Mediated Protodesilylation of Silyl Ethers (GP5) **S7**

2.6 Preparation of L*CuO*t*Bu stock solutions (GP6) **S7**

3. Reaction Monitoring Details **S8**

3.1 General Considerations and Model Reaction of (*S*)-**1a** **S8**

3.2 Summary of Varied Reaction Conditions of **1a** **S11**

3.3 Reaction without Styrene **S17**

3.4 Reaction of *rac*-**1a** **S19**

3.5 Dynamic Kinetic Resolution of *rac*-**1a** **S22**

3.6 Temperature Effects and Eyring Analysis **S25**

4. Substrate Variations and Intermolecular Competitions **S25**

4.1 Reactions of (*S*)-**1b**–**e** **S25**

4.2 Bigeleisen-Wolfsberg Analysis **S28**

4.3 Intermolecular Competition Reactions of (*S*)-**1a**–**e** **S29**

4.4 Hammett Correlation **S33**

4.5 Determination of k_S/k_R: Intermolecular Competition Reaction of (*S*)-**1a** vs (*R*)-**1a-C$_D$**, (*R*)-**1a** vs (*S*)-**1a-C$_D$**, and (*S*)-**1a** vs (*S*)-**1a-C$_D$** **S40**

4.6 Reaction of (*S*)-**1a-O$_D$** **S48**

4.7 Intermolecular Competition Reaction of (*S*)-**1a** vs (*S*)-**1a-O$_D$** and Kinetic Isotope Effect **S49**

4.8 Reaction of (*S*)-**1a** with $(n\text{Bu})_3$SiD **S51**

4.9 Intermolecular Competition Reaction of $(n\text{Bu})_3$SiH vs $(n\text{Bu})_3$SiD and Kinetic Isotope Effect **S54**

5. Catalytic System Investigations **S55**

5.1 Scalemic Mixtures of (*R*,*R*)-Ph-BPE and (*S*,*S*)-Ph-BPE **S55**

5.2 Reaction Initiation with CuO*t*Bu + (*R*,*R*)-Ph-BPE and MesCu + (*R*,*R*)-Ph-BPE **S57**

5.3 ^{1}H, ^{19}F and ^{31}P NMR Spectroscopy **S57**

6. Synthetic Procedures and Characterization Data **S62**

6.1 (*S*)-**1a** and (*R*)-**3a** **S62**

6.2 (*R*)-**1a** **S62**

6.3 (*S*)-**1c** **S63**

6.4 (*S*)-**1d** **S64**

6.5 (*S*)-**1e** **S65**

6.6 (*S*)-**1a-C$_D$** + (*R*)-**3a-C$_D$** **S66**

6.7 (*R*)-**1a-C$_D$** **S67**

6.8 (*S*)-**1a-O$_D$** **S67**

7. Computational Investigations **S69**

7.1 General Considerations **S69**

7.2 Aggregation of **1a** **S70**

7.3 Summary of Energies **S72**

8. Kinetic Modeling **S73**

8.1 General Considerations **S73**

8.2 Experimental Data Fits **S76**

8.3 Additional Boundary Conditions **S79**

9. References **S81**

1. General Considerations

Chemicals. Unless otherwise stated, the chemical reactions were assembled using standard air-free techniques under a static pressure of nitrogen (dried over phosphorus pentoxide) within a well-ventilated fume hood. 1-(4-Fluorophenyl)ethanol (**1a**, Acros Organics), and 4-fluoroanisole (Acros Organics), were distilled under vacuum before being introduced into a glovebox and dried over molecular sieves (4 Å) for at least 72 h before use. $(n\text{Bu})_3\text{SiH}$ (TCI) was dried over molecular sieves (4 Å) for at least 72 h before use. (*R*,*R*)-Ph-BPE (ABCR), (*S*,*S*)-Ph-BPE (ABCR), CuCl (99.999%, ABCR) were stored in a glovebox. NaO*t*Bu (ABCR) was sublimed under oil pump vacuum and stored in a glovebox. C_6D_6 (Eurisotop or Aldrich) was degassed by three freeze-pump-thaw cycles prior to introduction into a glovebox and subsequently dried over 4 Å molecular sieves for at least 72 h. Dry diethyl ether was obtained from distillation under N_2 atmosphere over sodium using benzophenone as an indicator. Anhydrous THF (Fisher Scientific, HPLC grade, unstabilized) and *n*-hexane (Aldrich, HPLC grade) were dispensed from an MBraun® solvent system (SPS-800) equipped with alumina columns under positive pressure of argon. Synthesized copper complexes and prepared stock solutions were stored at -40 °C in a glovebox freezer. Unless otherwise stated acetone (Fisher Scientific, reagent grade), dichloromethane (DCM, Aldrich, HPLC grade), *n*-hexane (Aldrich, HPLC grade), methyl tert-butyl ether (MTBE, Fisher Scientific, 99%) and magnesium sulfate ($MgSO_4$, Merck) were used as received. Synthetic procedures have not been optimized.

Chromatography. Analytical thin-layer chromatography (TLC) was performed on Macherey-Nagel Alugram® Xtra SIL G/UV254 silica gel 60 pre-coated aluminum-backed plates (200 µm layer thickness). Product spots were visualized under UV light (λ_{max} = 254 nm) and/or by staining with a ceric ammonium molybdate solution. Flash column chromatography was carried out on Grace 60 silica gel (30–63 µm, 230–400 mesh, ASTM) as a stationary phase. Silica was initially loaded as a slurry with the eluent. Eluents made of solvent mixtures were prepared by adding the corresponding volume of solvent per volume of the other using a measuring cylinder and shaking the mixture thoroughly before loading into the column. In-house N_2 gas was used to apply pressure. A dry loading technique was used to load crude mixtures into the column, using DCM (to partially dissolve the crude product), followed by careful evaporation in a rotary evaporator, using Celite® 545 (Aldrich) as the supporting material for sample preparation. **High performance liquid chromatography** (HPLC) to determine enantiomeric excesses (*ee*) was carried out on an Agilent Technologies 1290 Infinity instrument equipped with chiral stationary columns (Daicel Chiralcel® OJ-H, AD-H) using *n*-heptane/isopropanol mixtures as the mobile phase.

Stock solutions for kinetic experiments. Volumetric glassware, microsyringes, plastic consumables (syringes, syringe filters, vial caps, etc.), needles, and septa were dried at 60 °C

overnight before being transferred into the glovebox. Stock solutions were prepared by weighing the chemical reagents directly into volumetric flasks using analytical balances (± 0.01 mg weight precision). The required volume of the corresponding stock solution to prepare samples for kinetic experiments was measured with gas-tight syringes and detachable Sterican® needles.

NMR Spectroscopy. NMR spectra were acquired with a Bruker AV 500 at the chemistry institute of the Technische Universität Berlin, and with a Bruker Avance HD III 400 MHz spectrometer fitted with a 5 mm BBO Prodigy CryoProbe (LN_2) at the University of Edinburgh. The nuclear resonance frequencies of individual nuclei in MHz for the Bruker Avance HD III spectrometer are as follows: ^{1}H (400), ^{2}H (61) $^{13}C\{^{1}H\}$ (101), ^{19}F (377), ^{29}Si (79), and ^{31}P (162). The nuclear resonance frequencies of individual nuclei in MHz for the Bruker AV 500 spectrometer are as follows: ^{1}H (500), ^{2}H (77) $^{13}C\{^{1}H\}$ (126), ^{19}F (471), ^{29}Si (99), and ^{31}P (202). Spectra for characterization of air-stable species were acquired in borosilicate NMR tubes (O.D. ~ 5 mm) with PTFE caps at a probe temperature of 300 K unless otherwise stated. Spectra were recorded in $CDCl_3$ or C_6D_6 with the deuterated solvent acting as an internal deuterium lock. NMR tubes were dried overnight in a vacuum oven at 80 °C and either introduced into a glovebox or allowed to cool down at ambient temperature under air before use. NMR data were processed using MestReNova software (version 15.0.0). Chemical shifts are reported in parts per million (ppm). Integrations were performed after phase correction followed by baseline correction (Whittaker smoother). Characterization data were obtained by dissolving the sample in ~ 0.6 mL of deuteriated solvent. ^{1}H and ^{13}C NMR spectra were referenced using the residual protiated solvent signal (^{1}H) or deuteriated solvent carbon resonances (^{13}C): ($CHCl_3$: δ = 7.26 ppm for ^{1}H NMR and $CDCl_3$: δ = 77.16 ppm for ^{13}C NMR, C_6D_5H: δ = 7.16 ppm for ^{1}H NMR and C_6D_6: δ = 128.06 ppm for ^{13}C NMR), ^{19}F NMR chemical shifts are reported relative to $CFCl_3$ as 0.0 ppm as defined by the spectrometer's calibration. Abbreviations are as follows: singlet (s), doublet (d), triplet (t), doublet of doublets (dd), triplet of triplets (tt), sextet (hex), doublet of sextets (dhex), broad (br), and multiplet (m). When given, concentrations at each time point have been calculated by calibrating to an internal standard of known concentration, 4-fluoroanisole.

Infrared. IR spectra were recorded from neat samples on an Agilent Technologies Cary 630 FTIR spectrometer equipped with a diamond attenuated total reflectance (ATR) unit. Selected absorption maxima are reported in wavenumbers (cm^{-1}).

Kinetic Simulations and Numerical Methods. Kinetic models were fitted to experimental data using standard numerical approaches. Competition models were fitted by minimizing the sum of square residues using Excel Solver. All absolute and relative rate constant values are given with errors rounded to the nearest tenth, reflecting an overestimation of uncertainty.

2. General Procedures

2.1. Reaction Monitoring Experiments (GP1).

In an inert gas-filled glovebox, aliquots of C_6D_6 and of stock solutions in dry and degassed C_6D_6 containing the reagents for individual reactions were added to an NMR tube (J-Young) using gas-tight microsyringes. The NMR tube was tightly sealed, removed from the glovebox, shaken thoroughly, inserted into the spectrometer, locked to the deuterium signal of the solvent, and t = 0 s ^{1}H and ^{19}F{^{1}H} NMR spectra were recorded at the indicated probe temperature. The sample was reintroduced into the glovebox, and the J-Young valve was slowly opened, in which care was taken to minimize the amount of solution lost through removal of the valve. An aliquot of a stock solution containing catalytic components of the reaction was then added directly into the NMR tube and the time of the injection recorded. The sample was loaded into the NMR spectrometer and the locking routine repeated, after which ^{1}H and ^{19}F{^{1}H} spectra were taken alternatingly. The time of the first acquired ^{1}H spectrum was recorded and the time difference between the catalyst injection and the first acquisition adjusted for the t = 0 s spectra.

2.2. Preparation of *rac*-1a–e alcohols (GP2).

Following a modified literature procedure,[S1] in a round-bottom flask, the indicated ketone (1.0 equiv) is dissolved in methanol (1 mL/mmol), and the solution is cooled to 0 °C. $NaBH_4$ (1.0 equiv) is added portionwise, and the reaction mixture is stirred at 0 °C for 10 min and at room temperature for further 30 min. Water is added, and the aqueous phase is extracted three times with MTBE. The combined organic layers are washed with brine, dried over anhydrous $MgSO_4$, filtered, and concentrated under reduced pressure. The residue is purified by flash column chromatography on silica gel using cyclohexane:MTBE or *n*-pentane:diethyl ether mixtures as the eluent to afford the corresponding benzylic alcohol.

2.3. Kinetic Resolution of *rac*-1a–e alcohols by Enzymatic Acylation (GP3).

Following a modified literature procedure,[S2] the indicated alcohol (1.0 equiv) is dissolved in anhydrous *n*-hexane or toluene as indicated (1.0–1.5 mL/mmol) under an N_2 atmosphere, and Novozym® 435 (6 mg/mmol with respect to the alcohol) and vinyl acetate (0.2–0.6 equiv) are added sequentially. The reaction mixture is stirred at room temperature for the indicated period, and the conversion is monitored by ^{1}H NMR analysis. The reaction mixture is directly subjected to flash column chromatography on silica gel using cyclohexane:MTBE mixtures as the eluent to afford the corresponding enantioenriched acetate and alcohol.

2.4. General Procedure for the Base-Mediated Hydrolysis of Acetic Acid Esters (GP4).
The indicated acetate (1.0 equiv) is dissolved in methanol (2 mL/mmol), and a solution of NaOH (2.2 M in water, 4.4 equiv) is added. The reaction mixture is stirred at room temperature for the indicated period, and the conversion is monitored by TLC analysis, and the aqueous phase is extracted three times with MTBE. The combined organic layers are washed with water and brine, dried over $MgSO_4$, filtered, and concentrated. The residue is purified by bulb-to-bulb distillation to afford the corresponding alcohol.

2.5. General Procedure for the TBAF-Mediated Protodesilylation of Silyl Ethers (GP5).
A round-bottom flask containing a magnetic stir bar is charged with the indicated silyl ether (0.1 mmol, 1 equiv), and TBAF (1 M in THF, 0.5 mL, 0.5 mmol, 5 equiv) is added. The resulting mixture is stirred at room temperature for 30 min. Water (4 mL) is added, and the aqueous phase is extracted three times with MTBE (10 mL). The combined organic layers are dried over anhydrous $MgSO_4$, filtered, and concentrated under reduced pressure. The residue is purified by flash column chromatography on silica gel using cyclohexane:MTBE or *n*-pentane:diethyl ether mixtures as the eluent to afford the corresponding benzylic alcohol.

2.6. Preparation of L*CuO*t*Bu stock solutions (GP6).
In a glovebox, CuCl (1.0 equiv), NaO*t*Bu (1.0 equiv) and (*R*,*R*)-Ph-BPE (1.0 equiv) are weighed into a 6 mL screw cap vial containing a magnetic stirring bar, and C_6D_6 (10% of final stock solution volume) is added. The resulting suspension is stirred for 30 minutes at room temperature and then filtered using a small PTFE 0.45 μm syringe filter, and the filtrate dispensed directly into a volumetric flask. The parent vial was repeatedly rinsed with C_6D_6 (10% to 15% of final stock solution volume) and the resulting solution used to wash the syringe filter via dispensing into a volumetric flask. This procedure is repeated until the total stock solution volume is reached. The resulting yellow solution of precatalyst L*CuO*t*Bu is transferred to a clean medium-sized vial, tightly sealed, and stored inside a glovebox freezer (–30 °C to –40 °C).

3. Reaction Monitoring Details

3.1 General Considerations and Model Reaction of (*S*)-1a

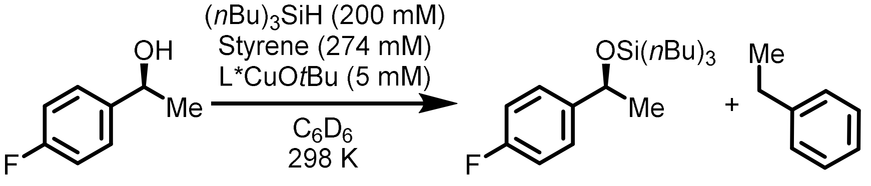

(*S*)-**1a**
200 mM

(*S*)-**2a**

PhEt

In an inert gas-filled glovebox, a 900 µL sample containing (*S*)-1-(4-fluorophenyl)ethanol, (*S*)-**1a**, 4-fluoroanisole, (nBu)$_3$SiH, and styrene in C_6D_6 was prepared according to GP1, and ^{1}H and ^{19}F{^{1}H} NMR t = 0 s spectra of the sample were acquired. The sample was reintroduced into the glovebox and the reaction started through addition of an aliquot of a stock solution containing the precatalyst L*CuO*t*Bu (100 µL, solution prepared via GP6). The sample was reinserted into the NMR spectrometer, and multiple single scan ^{1}H and ^{19}F{^{1}H} spectra were acquired in alternation. Representative labelled ^{1}H NMR spectra for the reaction depicted in Table S1, Entry 1 are shown below: before injection of the precatalyst stock solution (Figure S1; t = 0 s) and at approximately one reaction half-life (Figure S2; t = 9386 s), and the complete series of ^{1}H NMR spectra (Figure S3). Similarly, equivalent ^{19}F{^{1}H} NMR spectra are shown (Figures S4–S6)

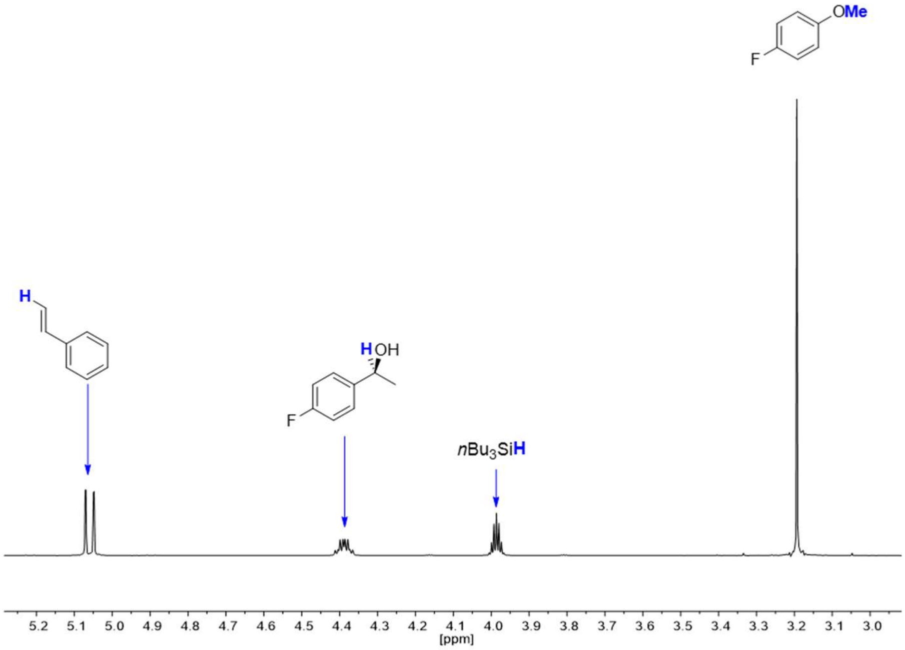

Figure S1. ^{1}H NMR spectrum of the reaction mixture before initiation (t = 0 s). The aromatic and aliphatic regions were omitted from the spectrum for clarity. Protons chosen for reaction monitoring purposes were highlighted in blue.

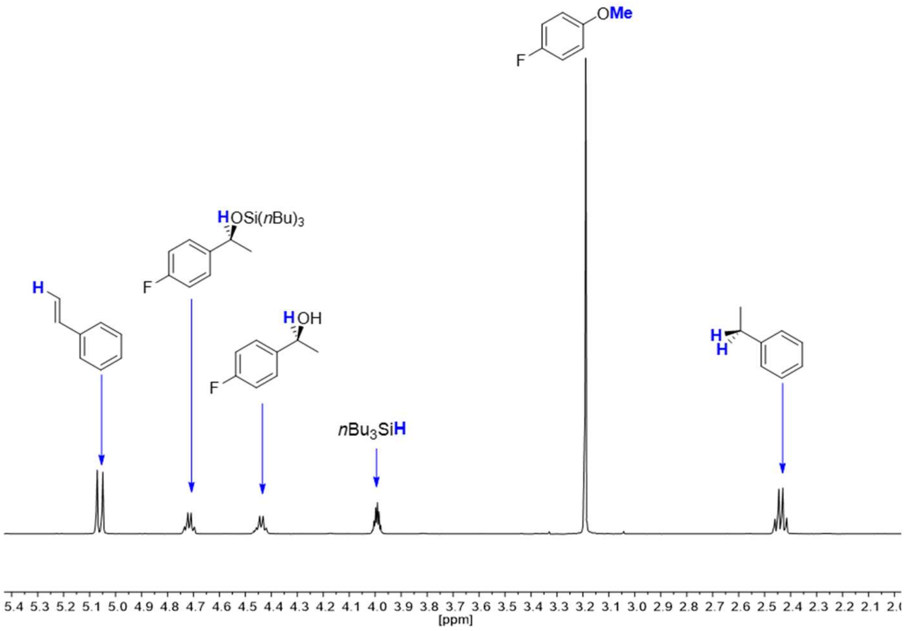

Figure S2. ^{1}H NMR spectrum of the reaction mixture at approximately one reaction half-life (t = 9386 s). The aromatic and aliphatic regions were omitted from the spectrum for clarity. Protons chosen for reaction monitoring purposes were highlighted in blue.

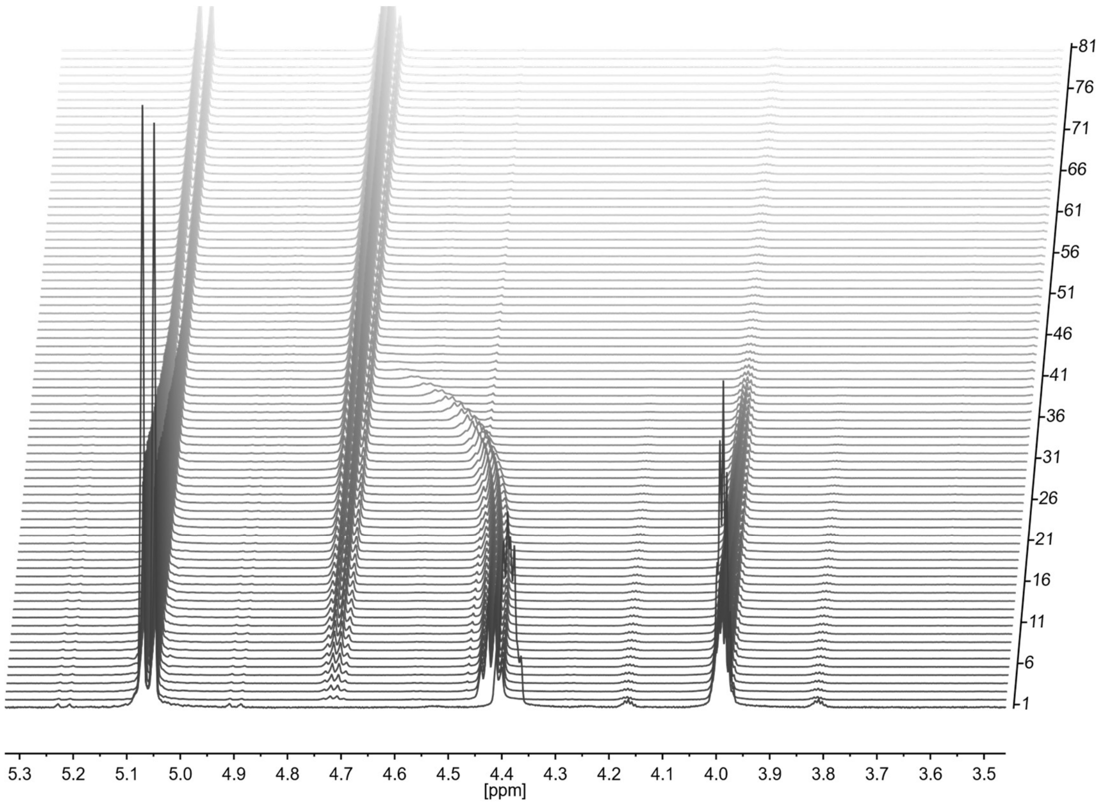

Figure S3. Stacked ^{1}H NMR spectra of the reaction mixture over the course of the complete reaction. The aromatic and aliphatic regions were omitted from the spectrum for clarity. From left to right, signals depicted are those of styrene, (1-(4-fluorophenyl)ethoxy)tri-*n*-butylsilane, (*S*)-**2a**, 1-(4-fluorophenyl)ethanol, (*S*)-**1a**, and (nBu)$_3$SiH.

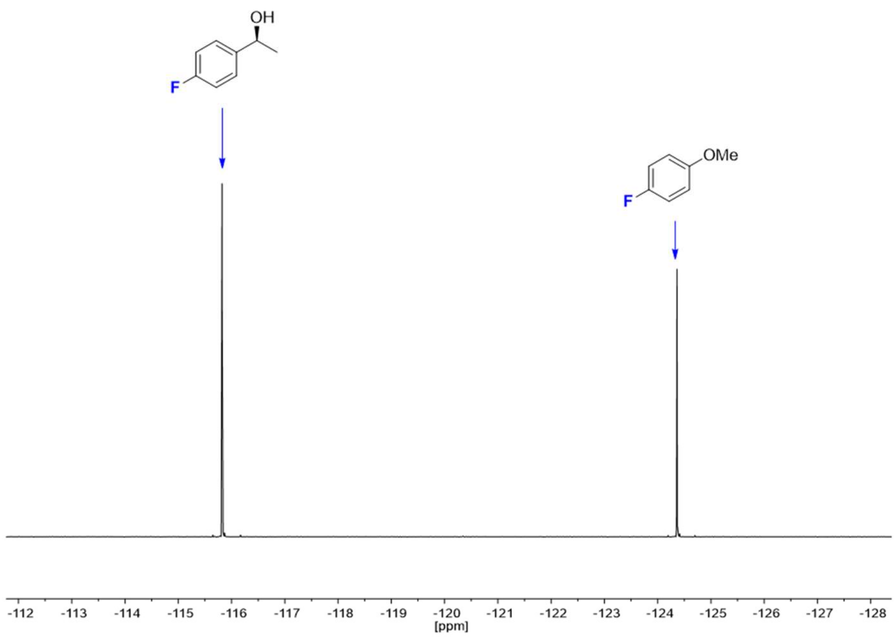

Figure S4. $^{19}F\{^{1}H\}$ NMR spectrum of the reaction mixture before initiation (*t* = 0 s). No other ^{19}F NMR signals are present in the spectrum. Fluorine atoms were highlighted in blue.

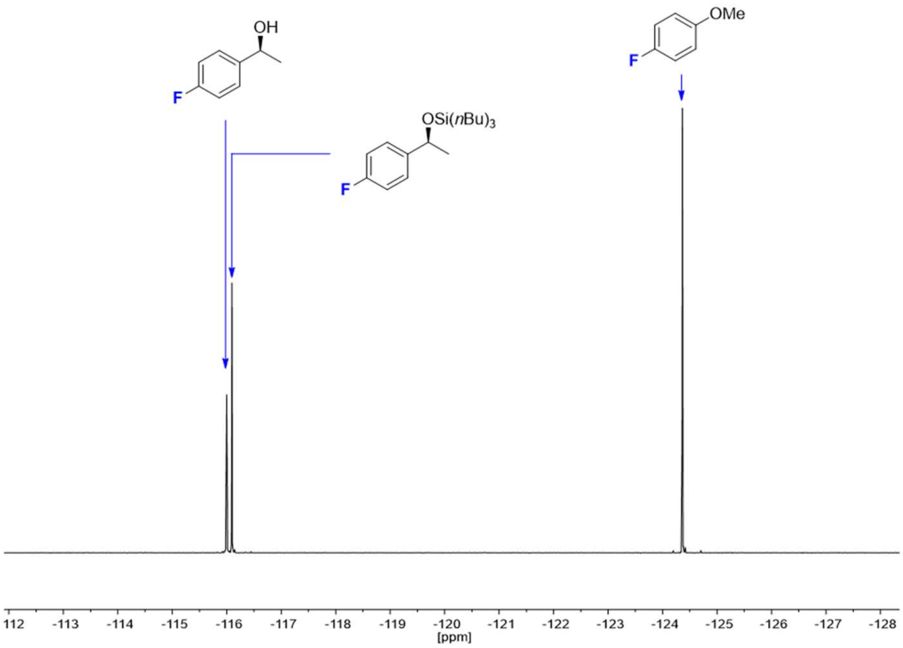

Figure S5. $^{19}F\{^{1}H\}$ NMR spectrum of the reaction mixture at approximately one reaction half-life (*t* = 9600 s). No other ^{19}F NMR signals are present in the spectrum. Fluorine atoms were highlighted in blue.

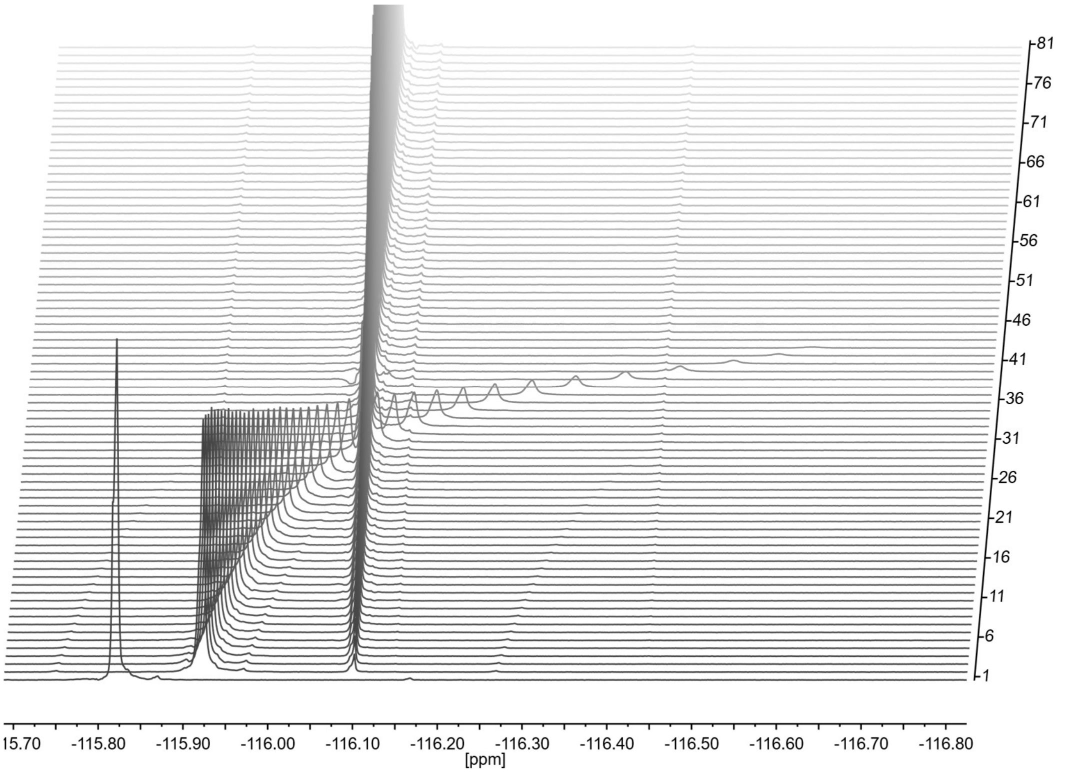

Figure S6. Stacked $^{19}F\{^{1}H\}$ NMR spectra of the reaction mixture over the course of the complete reaction. From left to right (at time of second spectrum), signals depicted are those of 1-(4-fluorophenyl)ethanol, (*S*)-**1a**, and (1-(4-fluorophenyl)ethoxy)tri-*n*-butylsilane, (*S*)-**2a**. The 4-fluoroanisole signal was omitted for clarity.

3.2. Summary of Varied Reaction Conditions of 1a

The conditions of various reaction monitoring experiments were systematically varied from those of the model experiment described in Section 3.1 (Table S1, Entry 1) and were summarized in Table S1. The initial rates of reaction (v_0) obtained from each experiment are given. Values for v_0 were obtained from a linear regression of all concentrations of **2a** up to 75% conversion for Entries 1, 2, 3, 10–16, and 19–23 where reaction profiles are typical of overall zeroth order reactions and from the first five product concentrations for Entries 4–9, 17, and 18, where reaction profiles deviate significantly from linearity. Substrate and L*CuO*t*Bu initial concentrations significantly varied from those in Entry 1 are highlighted in blue for clarity and v_0 values for each experiment were given. v_0 values which significantly deviate from that of Entry 1 are highlighted (green for higher, red for lower values). Temporal concentration profiles are depicted in Figures S7 (Entries 1–6), S8 (Entries 7–12), S9 (Entries 13–18), and S10 (Entries 19–23).

Table S1. Initial concentrations of reactants and initial rates of reaction monitoring experiments

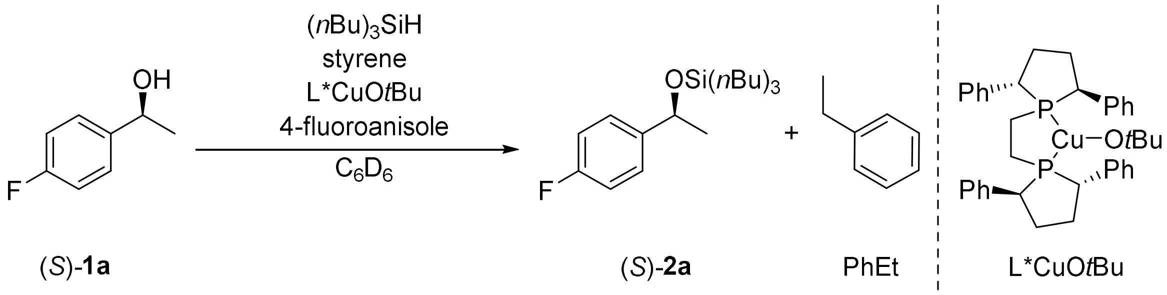

Entry	[(*S*)-**1a**]$_0$ [M]	[(nBu)$_3$SiH]$_0$ [M]	[styrene]$_0$ [M]	[L*CuO*t*Bu]$_0$ [M]	[4-fluoroanisole]$_0$ [M]	*T* [K]	$v_0 \cdot 10^5$ [M·s^{-1}]
1	0.200	0.200	0.274	0.005#1	0.148	298	1.0
2	0.200	0.200	0.270	0.005#2	0.160	298	0.99
3	0.200	0.200	0.292	0.005#3	0.182	298	1.1
4	0.194	**0.101**	0.276	0.005#1	0.148	298	**0.56**
5	0.196	**0.267**	0.278	0.005#1	0.148	298	**1.4**
6	0.194	**0.404**	0.277	0.005#1	0.148	298	**1.9**
7	**0.318**	0.200	0.270	0.005#1	0.157	298	**0.60**
8	**0.410**	0.200	0.270	0.005#1	0.160	298	**0.43**
9	**0.630**	0.203	0.271	0.005#1	0.154	298	**0.24**
10	0.199	0.203	**0.393**	0.005#2	0.054	298	1.0
11	0.203	0.203	**0.659**	0.005#2	0.054	298	0.96
12	0.191	0.199	0.265	**0.0100**#1	0.154	298	**2.2**
13	0.202	0.203	0.273	**0.0075**#1	0.160	298	**1.5**
14	0.200	0.209	0.270	**0.0025**#1	0.157	298	**0.47**
15	**0.297**	**0.300**	**0.364**	0.005#2	0.037	298	0.99
16[a]	0.201	0.200	0.327	0.005#2	0.043	298	**0.24**
17[b]	0.201	0.200	**0.000**	0.005#4	0.025	298	1.0
18[c]	0.200	0.200	0.310	0.005#1	0.028	298	**0.69**
19	0.200	0.200	0.308	0.005#4	0.034	**288**	**0.40**
20	0.200	0.200	0.308	0.005#4	0.034	**293**	**0.62**
21	0.200	0.200	0.307	0.005#4	0.034	**298**	0.95
22	0.200	0.200	0.316	0.005#4	0.034	**303**	**1.5**
23	0.200	0.200	0.319	0.005#4	0.034	**308**	**2.2**

[a] Reaction conducted with (*R*)-**1a**
[b] Reaction conducted without styrene, see Figure S11
[c] Reaction conducted with *rac*-**1a**
[L*CuO*t*Bu]#*n* Reactions with identical *n* values were conducted with identical stock solutions of L*CuO*t*Bu (see Section 5)

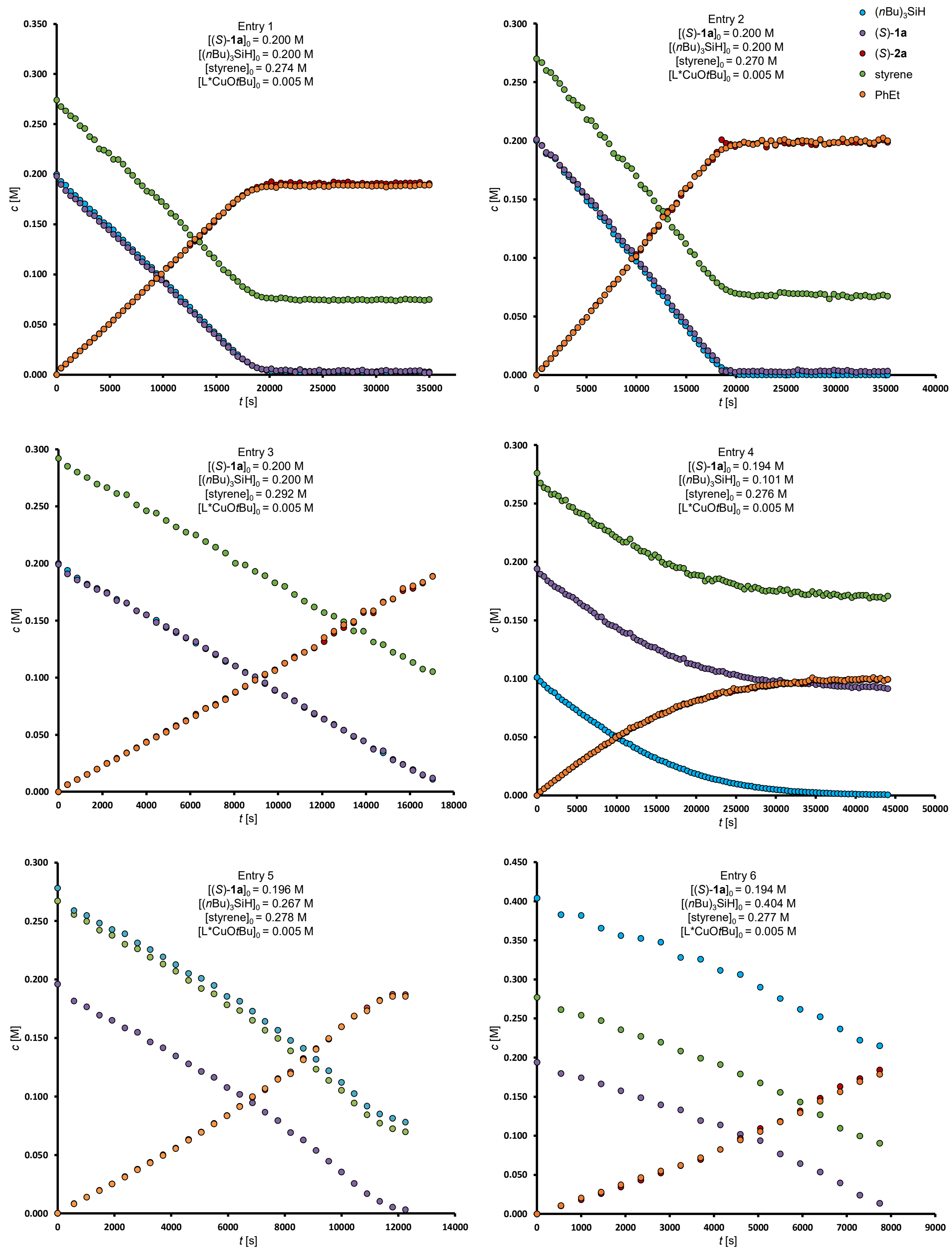

Figure S7. Temporal concentration profiles of reactants and products in reactions of Table S1, Entries 1–6.

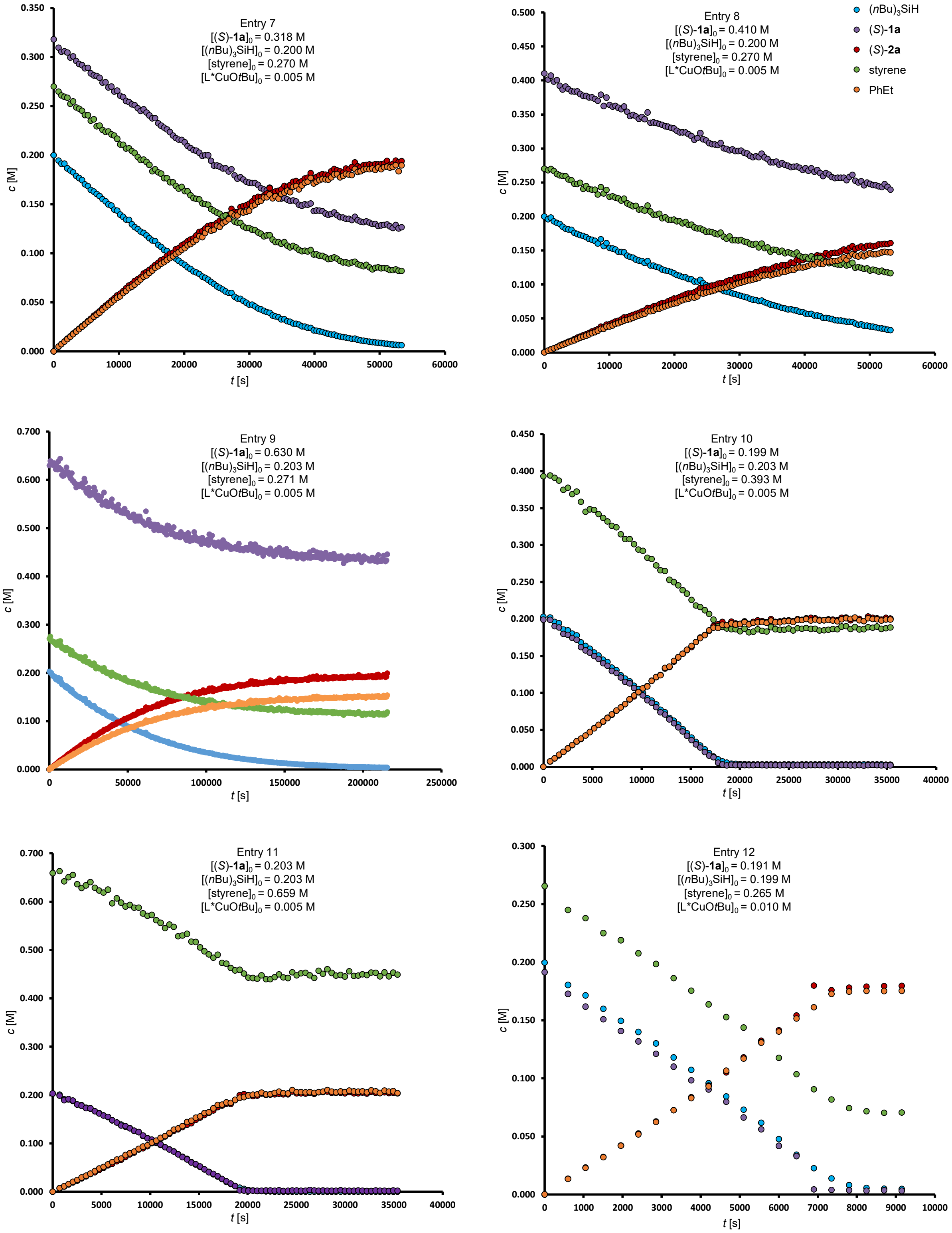

Figure S8. Temporal concentration profiles of reactants and products in reactions of Table S1, Entries 7–12.

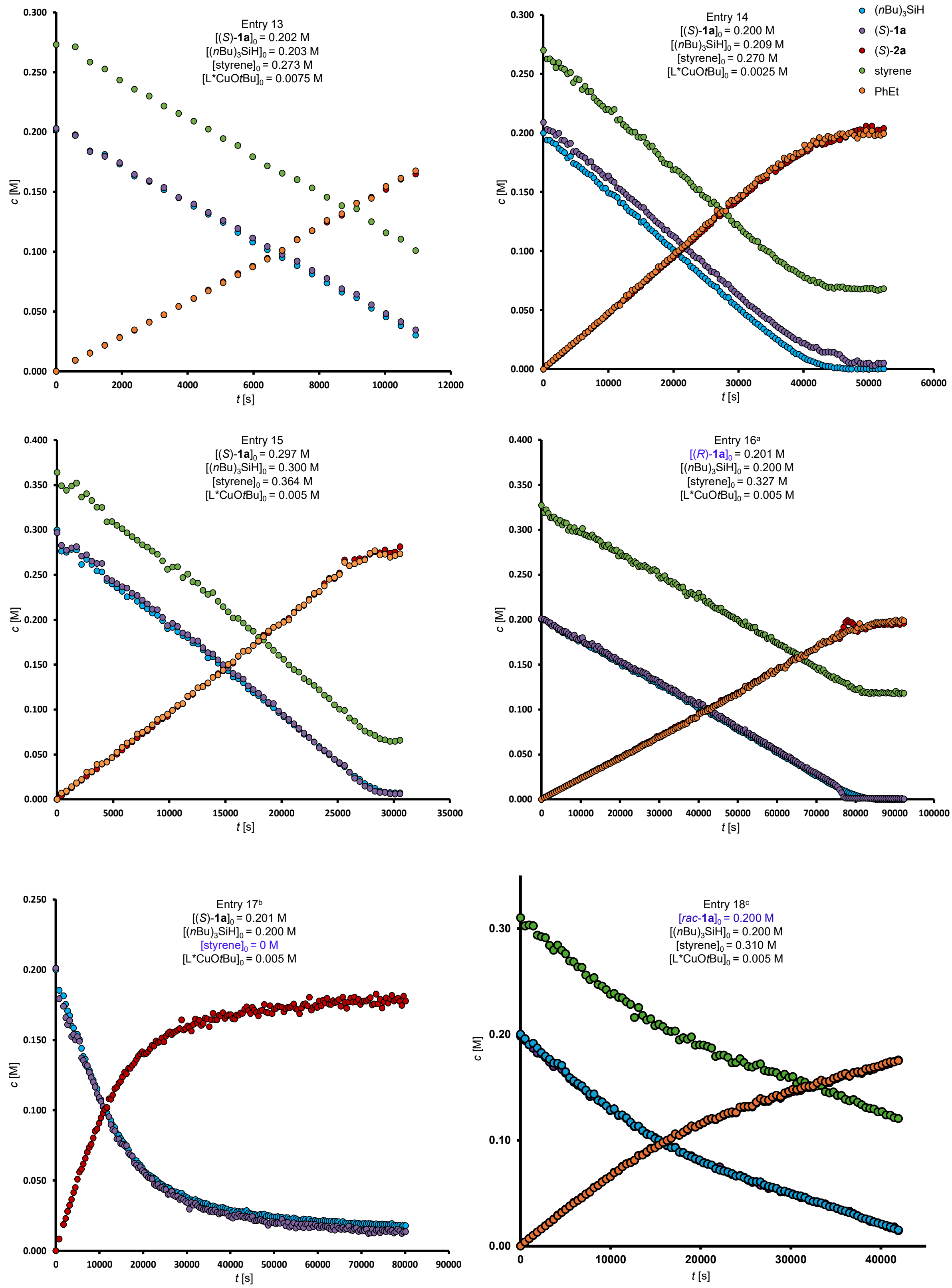

Figure S9. Temporal concentration profiles of reactants and products in reactions of Table S1, Entries 13–18. [a]: Reaction with enantiopure (*R*)-**1a**. [b]: Reaction without styrene, see Section 3.3 for discussion. [c]: Reaction with *rac*-**1a**, see Section 3.4 for discussion.

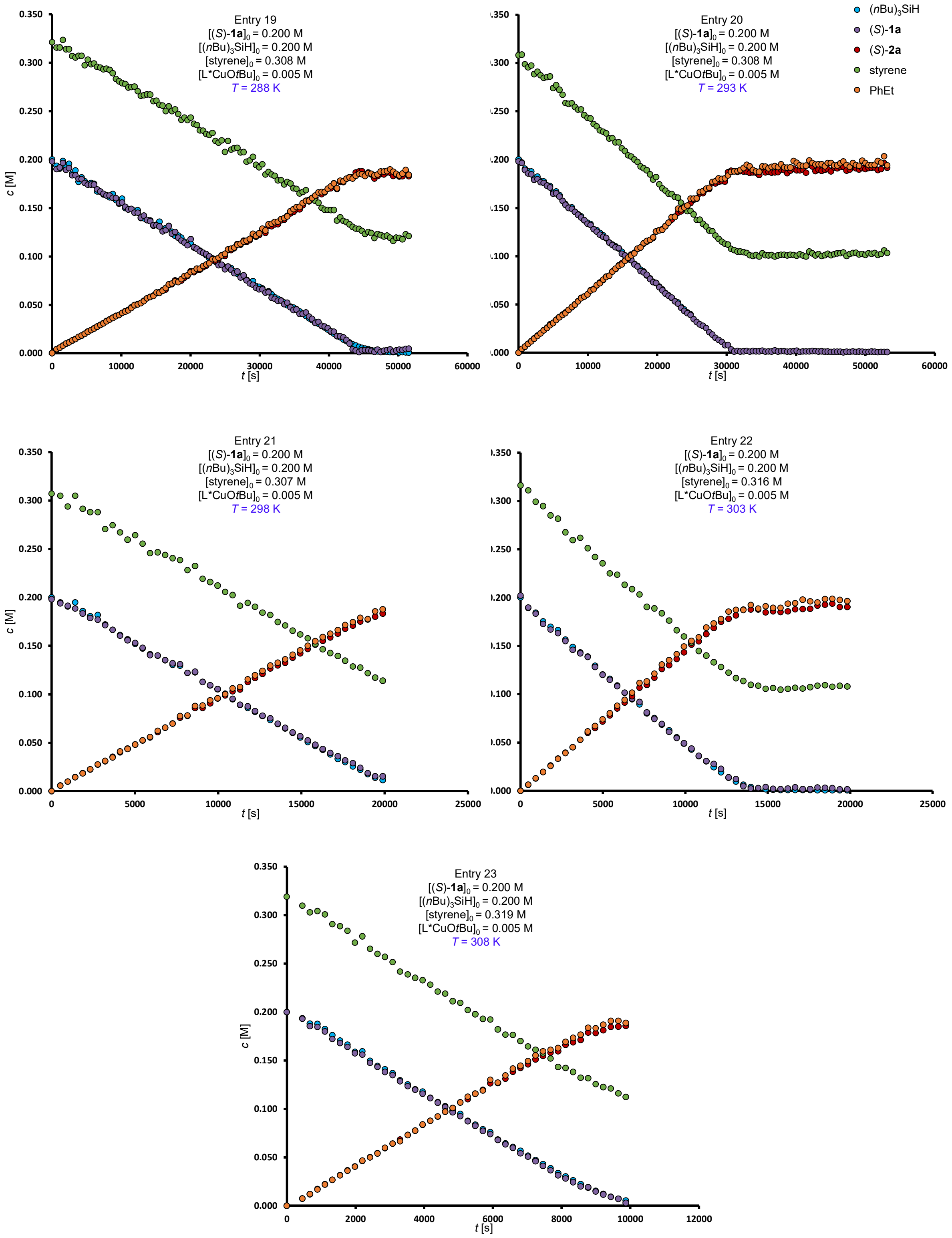

Figure S10. Temporal concentration profiles of reactants and products in reactions of Table S1, Entries 19–23.

3.3. Reaction Without Styrene

The reaction run in the absence of styrene (Table S1, entry 17) in a thick-wall NMR tube (Norell S-5-500-HW-IPV-8 NMR tube for intermediate pressures) resulted in an initially similar time concentration profile (see Figure S11 for comparison) to a reaction in the presence of styrene (Table S1, entry 1). After approximately 40% conversion, a progressive deviation from linearity was observed in the time-concentration profile and the reaction eventually appears to stall completely. Concurrently, stoichiometric amounts of H_2 gas relative to the production of (*S*)-**2a** are observed up to 40% conversion with a small, acute drop in H_2 concentration, potentially indicating rapid gas extrusion from solution, and slow, progressive reduction of H_2 concentration. Reintroduction of the NMR tube into a glovebox followed by venting excess H_2 gas (after $t \approx 85000$ s) did not affect the ensuing reaction rate. A change in chemical shift of (*S*)-**1a** like that observed under standard conditions (Figure S3) is initially present with a downfield shift ($\delta_H = 4.41$ ppm), but the trend reverses and the resonance of (*S*)-**1a** converges to its initial value ($\delta_H = 4.38$ ppm). This is consistent with a progressive decrease in concentration of copper species able to interact with (*S*)-**1a**. The absence of styrene results in (*S*)-**1a** being directly deprotonated by a copper hydride species. (see Figure 8 in main manuscript). For (*S*)-**1a**, this step is modeled to be approximately 20 times slower than styrene insertion into CuH, potentially leading towards accumulation of monomeric CuH.

Venting of excess H_2 did not result in an increase in rate, and therefore a further irreversible step which does not involve H_2 is likely to be present. Inclusion of an irreversible CuH dimerization step ($k = 0.3$ $M^{-1}\cdot s^{-1}$) to the kinetic model (see Figure 8 in main manuscript) resulted in good agreement with the observed reaction kinetics in Figure S11. Dimerization of monomeric CuH is computationally predicted to be thermodynamically favorable ($\Delta G = -29.4$ $kcal\cdot mol^{-1}$), although a transition state for this transformation could not be directly located on the electronic energy potential surface. Furthermore, the transiency and low concentrations of L*CuH in the presence of styrene causes dimerization to be kinetically unfavorable (see Section 8.1 for details).

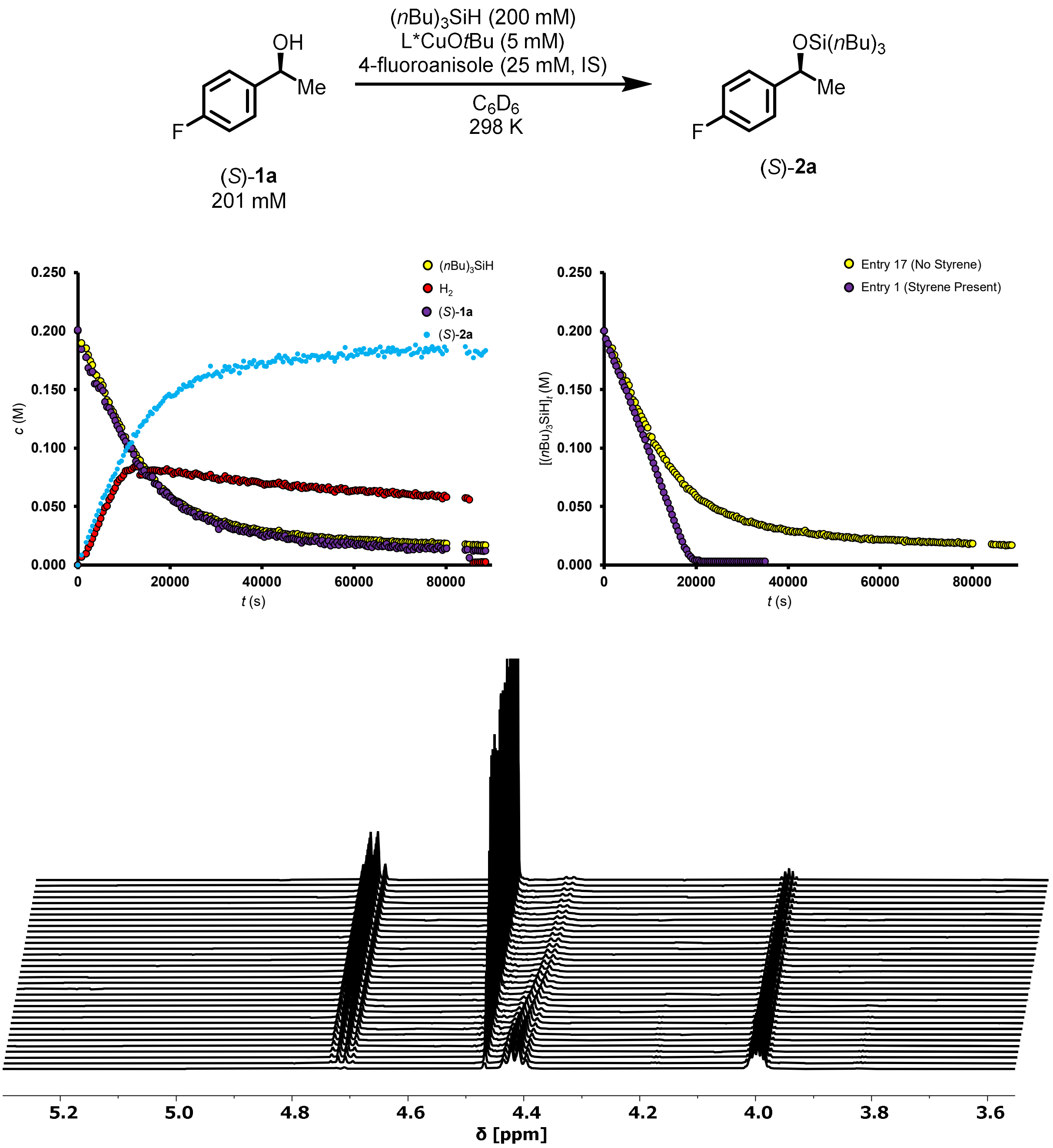

Figure S11 *Left Plot*: Temporal concentration profiles of reactants and products in Table S1, Entry 17, for the reaction without styrene. H_2 concentrations are not accurately reflected due to its low benzene solubility[S3] and due to venting of the NMR tube after $t \approx 85000$ s, *Right Plot*: Comparison of temporal concentration profiles of (nBu)$_3$SiH in reactions with (Table S1, Entry 1) and without (Table S1, Entry 17) styrene. *Bottom*: Stacked ^{1}H NMR spectra of the reaction mixture without styrene over the course of the complete reaction. The aromatic and aliphatic regions were omitted from the spectrum for clarity. From left to right, signals depicted are those of (S)-**2a**, H_2, (S)-**1a**, and (nBu)$_3$SiH.

3.4. Reaction of *rac*-1a

The reaction run with racemic **1a** (Table S1, entry 18, and Figure S12) revealed that separate quantification of (*R*)-**1a** and (*S*)-**1a** was possible with ^{19}F NMR spectroscopy (Figure S13), as the time-averaged resonances of each enantiomer were significantly separated (Figure S14). Deconvolution showed that a distinct linear regime suggested in the main text to consist solely of (*R*)-**1a** (Figure 3) is operative.

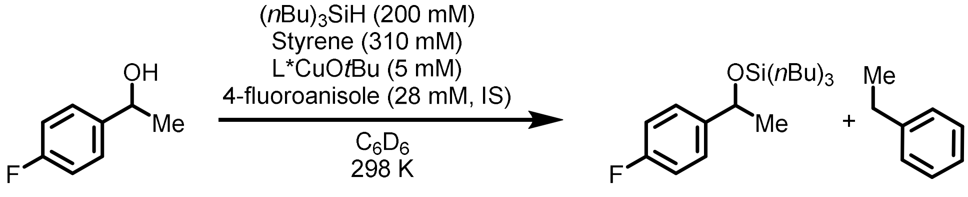

rac-**1a**
200 mM

rac-**2a**

PhEt

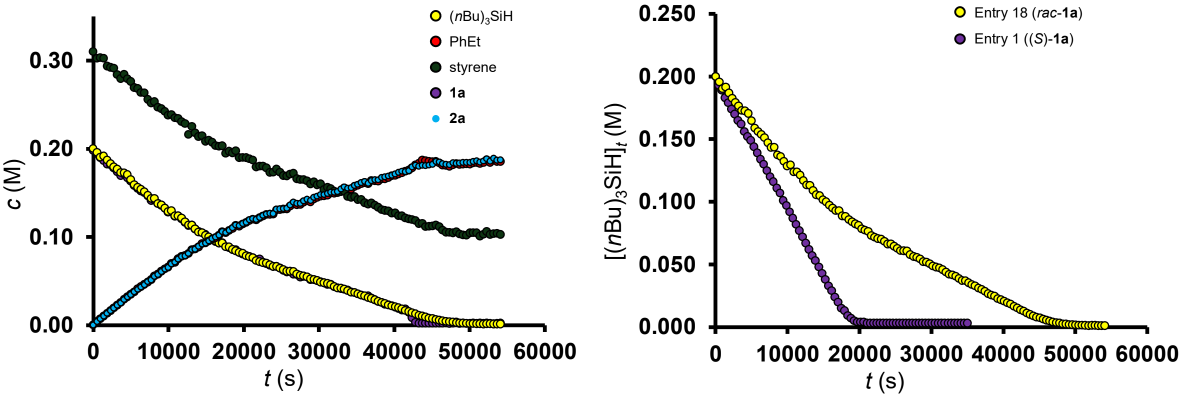

Figure S12 *Left Plot*: Temporal concentration profiles of reactants and products in Table S1, Entry 18, for the reaction with *rac*-**1a**. Temporal-concentration profiles depicted **1a** and **2a** where (*S*)-, (*R*)-, or *rac*- are not specified indicate scalemic mixtures. *Right Plot*: Comparison of temporal concentration profiles of $(nBu)_3SiH$ in reactions of enantiopure (*S*)-**1a** (Table S1, Entry 1) and of *rac*-**1a** (Table S1, Entry 18) as starting materials.

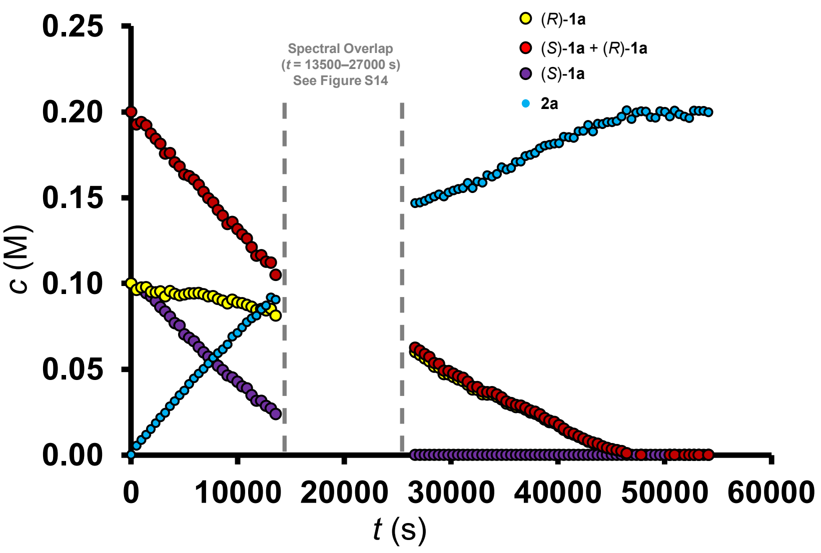

Figure S13 Temporal concentration profiles of reactants and products in Table S1, Entry 18, for the reaction with *rac*-**1a** monitored with ^{19}F NMR spectroscopy. Temporal-concentration profiles of (*S*)-**1a** and (*R*)-**1a** are obtained from separate integration of their respective time-averaged resonances (see Figure S14). The gap from *t* = 13500–27000 s was neglected due to significant overlap in resonances of **2a** and **1a** enantiomers (see Figure S14).

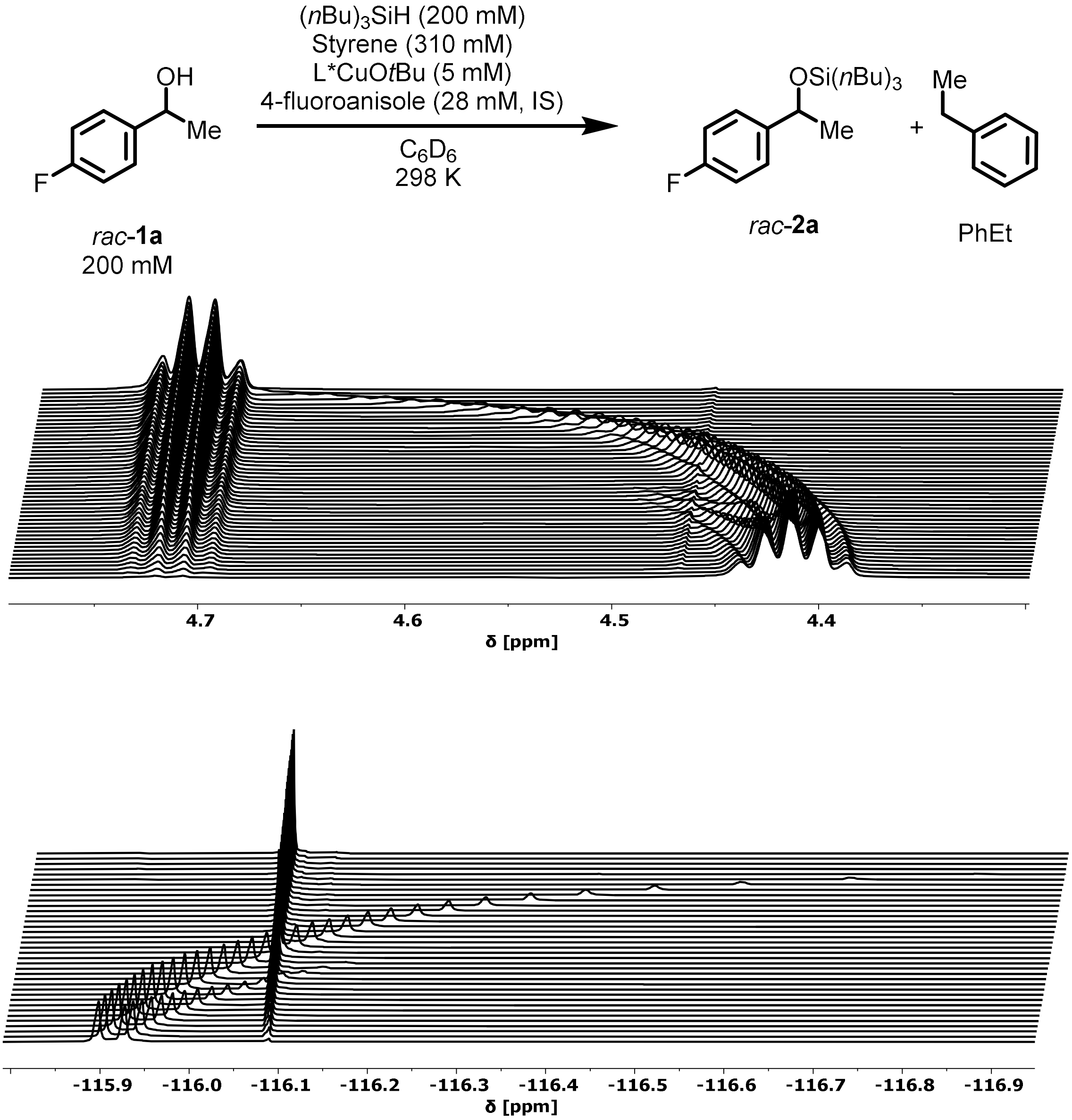

Figure S14 Stacked NMR spectra (*Top*: ^{1}H, *Bottom*: ^{19}F) of the reaction mixture from Table S1, Entry 18 over the course of the complete reaction. The aromatic and aliphatic regions were omitted from the spectrum for clarity. Signals depicted in ^{1}H spectra, from left to right, are those of **2a**, and the time-averaged resonances of (*S*)-**1a**, and (*R*)-**1a**. Signals depicted in ^{19}F spectra, from left to right, are the time-averaged resonances of (*R*)-**1a**, (*S*)-**1a**, and the signal of **2a**.

3.5 Dynamic Kinetic Resolution of *rac*-1a

Inside a glovebox, a 2 mL screw top vial was charged with NaO*t*Bu (2.4 mg, 25 µmol, 2.5 mol%) and Ru(CNN)(dppb)Cl[S4] (19 mg, 25 µmol, 2.5 mol%). A solution of *rac*-1-(4-fluorophenyl)ethan-1-ol (*rac*-**1a**, 140.2 mg, 1.0 mmol, 1.0 equiv) in C_6D_6 (0.3 mL) was added, and the parent vial was rinsed four times with 0.15 mL of C_6D_6. The mixture was stirred at room temperature for 10 min. The resulting red solution was filtered over glass wool into a 2 mL volumetric flask, the parent vial and glass wool were rinsed five times with 0.2 mL of C_6D_6, and C_6D_6 was added until the 2 mL mark was reached. Subsequently, 400 µL of the solution were added to a J-Young NMR tube, followed by 400 µL of a stock solution of $(n\text{Bu})_3$SiH, styrene, and 4-fluoroanisole (0.50 M, 0.76 M, and 0.25 M, respectively, in C_6D_6), and by 400 µL of neat C_6D_6. The NMR tube was sealed, removed from the glovebox, well shaken, inserted into a Bruker Avance III 500 MHz NMR spectrometer, and a ^{1}H reference spectrum for t = 0 s was recorded using the acquisition parameters stated above. The sample was reintroduced into the glovebox, and the cap was carefully removed, making sure as little as possible of the containing solution adhered to the cap and the top part of the NMR tube. Then, 100 µL of a stock solution of L*CuO*t*Bu (0.05 M in C_6D_6) were added to the NMR tube, and the exact time of addition was noted. The NMR tube was sealed, shaken vigorously, removed from the glovebox, and reinserted into the NMR spectrometer. The reaction progress was monitored by repeated alternating acquisition of ^{1}H and ^{19}F{^{1}H} NMR spectra. The initial concentrations were determined to be 0.197 M for *rac*-1-(4-fluorophenyl)ethan-1-ol (*rac*-**1a**), 0.307 M for styrene, 0.104 M for 4-fluoroanisole, and 0.200 M for tri-*n*-butylsilane. Initial concentrations of L*CuO*t*Bu and Ru(CNN)(dppb)O*t*Bu were 0.005 M.

Unlike in the KR reaction (Section 3.4), the ^{1}H and ^{19}F intensities of *rac*-**1a** showed no significant difference in the rate of consumption of either enantiomer. In ^{1}H, a pseudo-pentet was present throughout the reaction, and in ^{19}F the resonances of individual enantiomers were shown to maintain a constant ratio of intensities throughout the reaction, showing that racemization of **1a** under these conditions is significantly faster than irreversible reactions of **1a**. Furthermore, addition of Ru(CNN)(dppb)O*t*Bu resulted in a time-concentration profile of *rac*-**1a** virtually identical to that of enantiopure (*S*)-**1a** under comparable reaction conditions (Figure S15). This could either indicate that both conditions share the same catalyst resting state, L*CuOR^S·(*S*)-**1a**, or that a resting state involving (*S*)-**1a** comparable to the ground state energy of L*CuOR^S·(*S*)-**1a** is present, such as either L*CuOR^R·(*S*)-**1a** or L*CuOR^S·(*R*)-**1a**. Computationally (see Figure 9 in the main manuscript for details), L*CuOR^S·(*R*)-**1a** (ΔG = −3.6 kcal·mol^{-1} relative to L*CuOR^S + (*R*)-**1a**) is predicted to have the same inhibition properties as L*CuOR^S·(*S*)-**1a** (ΔG = −3.5 kcal·mol^{-1} relative to L*CuOR^S + (*S*)-**1a**), whereas

L*CuOR^R·(*S*)-**1a** (ΔG = −3.5 kcal·mol^{-1} relative to L*CuOR^S + (*R*)-**1a**) is predicted to be a less stable resting state than L*CuOR^S·(*R*)-**1a** or L*CuOR^S·(*S*)-**1a**.

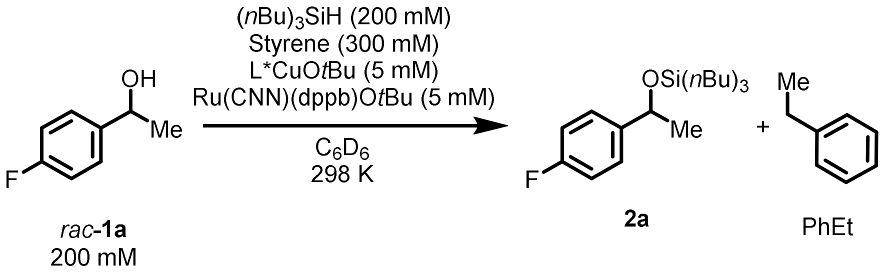

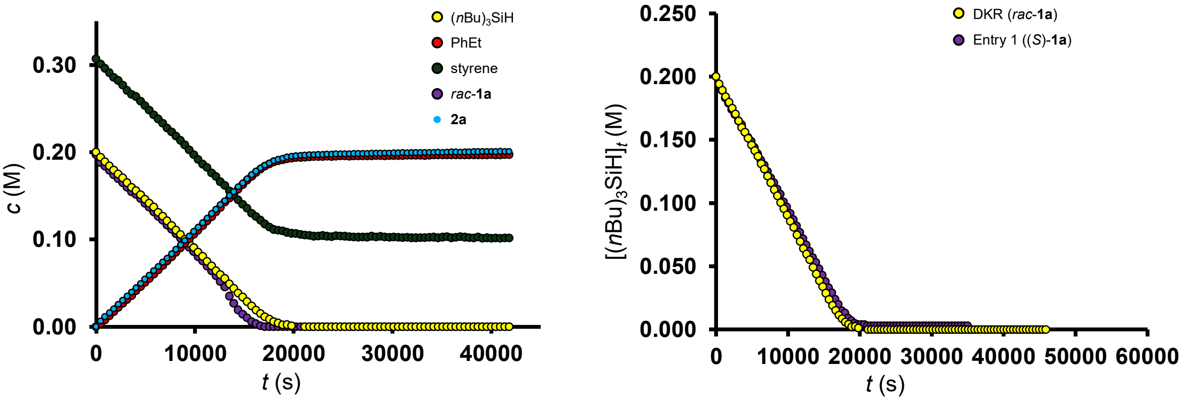

Figure S15: *Left Plot*: Temporal concentration profiles of reactants and products of the DKR of *rac*-**1a**. Temporal-concentration profiles depicted **1a** and **2a** where (*S*)-, (*R*)-, or *rac*- are not specified indicate scalemic mixtures. *Right Plot*: Comparison of temporal concentration profiles of (nBu)$_3$SiH in reactions of enantiopure (*S*)-**1a** (Table S1, Entry 1) and of *rac*-**1a** (under DKR conditions) as starting materials.

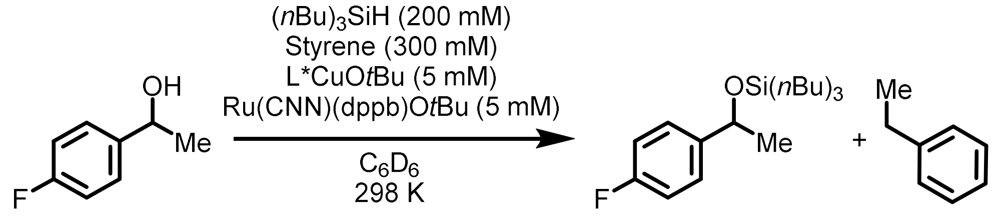

rac-**1a**
200 mM

2a

PhEt

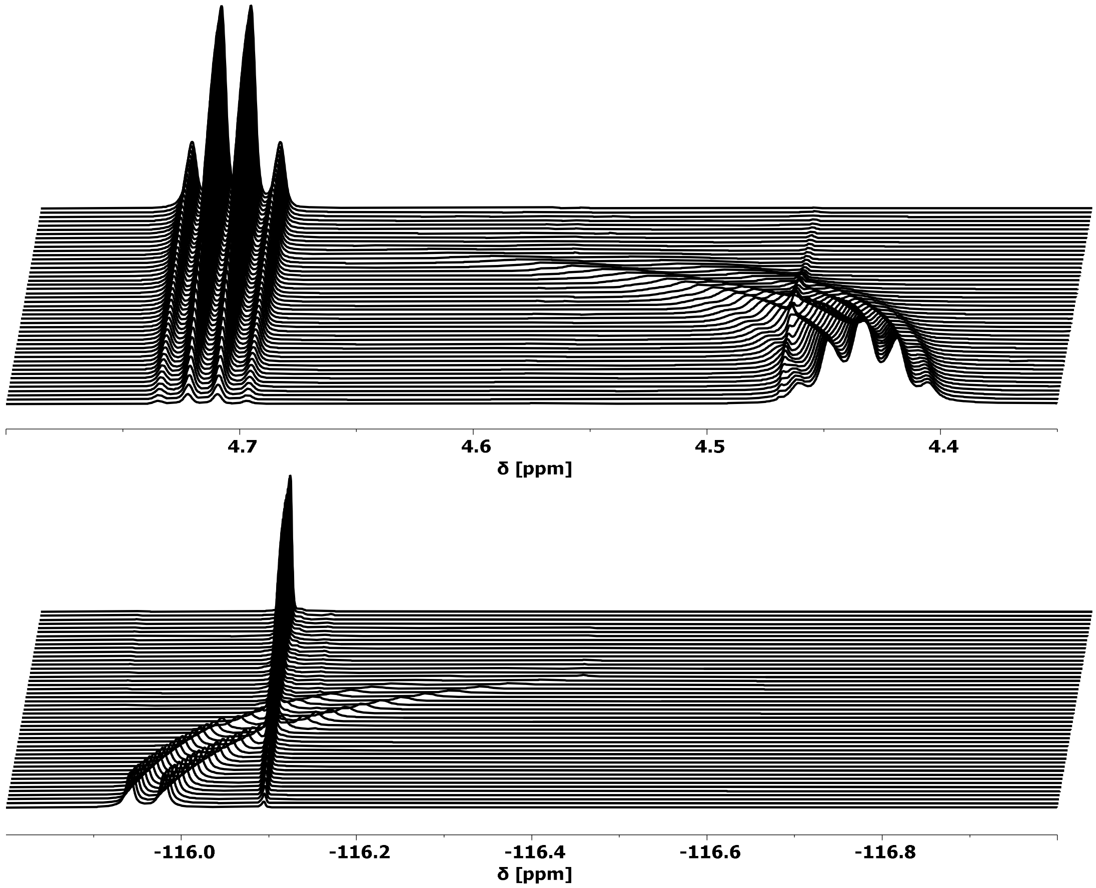

Figure S16: Stacked NMR spectra (*Top*: ^{1}H, *Bottom*: ^{19}F) of the DKR of *rac*-**1a** (Section 3.5)over the course of the complete reaction. The aromatic and aliphatic regions were omitted from the spectrum for clarity. Signals depicted in ^{1}H spectra, from left to right, are those of **2a**, and the time-averaged resonances of (*S*)-**1a**, and (*R*)-**1a**. Signals depicted in ^{19}F spectra, from left to right, are the time-averaged resonances of (*R*)-**1a**, (*S*)-**1a**, and the signal of **2a**.

3.6 Temperature Effects and Eyring Analysis

With the data obtained and summarized in Table S1 (Entries 19 to 23), the empirical activation parameters for turnover were estimated via Eyring analysis (288–308 K). Empirical coefficients (k_{rxn}/K_i, see Equation 1 in main manuscript) were determined from the respective v_0 values at each temperature and the initial catalyst concentrations of 0.005 M, $k_{rxn}/K_i \approx v_0/(0.005\ \text{M})$. The activation parameters, $\Delta G^‡$, $\Delta H^‡$ and $\Delta S^‡$ are shown in Figure S17.

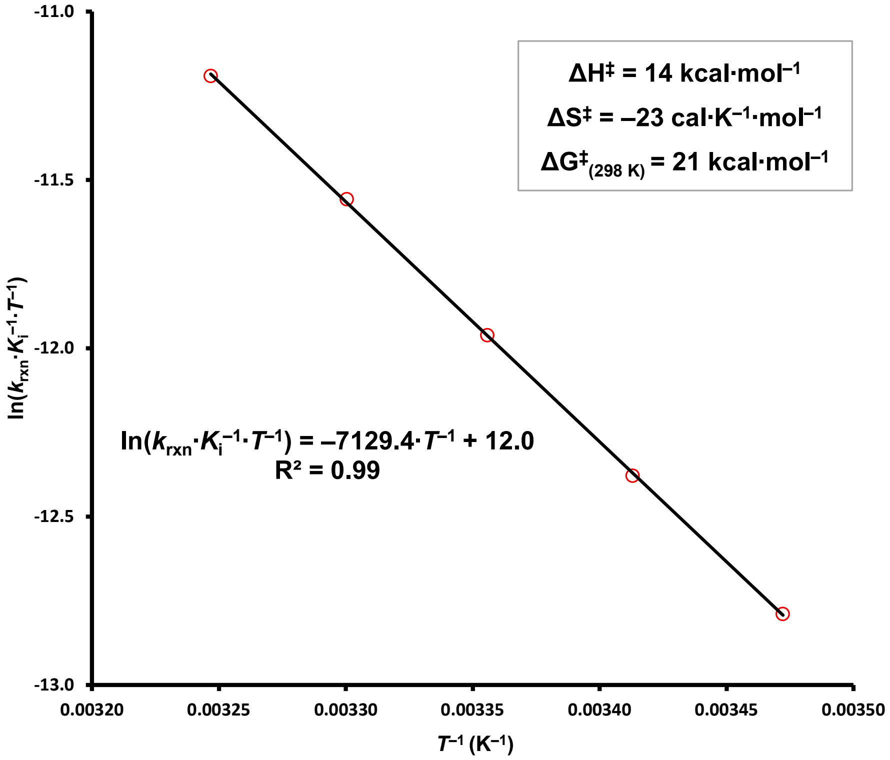

Figure S17 Eyring analysis with data from Table S1, Entries 19–23, see Figure S10 for time-concentration plots.

4. Substrate Variations and Intermolecular Competitions

4.1 Reactions of (*S*)-1b–e

Individual reaction mixtures of the enantiopure alcohols (*S*)-1-(phenyl)-ethan-1-ol ((*S*)-**1b**), (*S*)-1-(4-methylphenyl)ethan-1-ol ((*S*)-**1c**), (*S*)-1-(4-methoxyphenyl)ethan-1-ol ((*S*)-**1d**), and (*S*)-1-(4-(trifluoromethyl)phenyl)ethan-1-ol ((*S*)-**1e**), (nBu)$_3$SiH, styrene, and 4-fluoroanisole in C_6D_6 were assembled in J-Young NMR tubes according to general procedure GP1. Each reaction was monitored at 298 K. Initial conditions and temporal concentration profiles of each reaction are shown in Figures S18–S21. A temporal concentration profile of tri-*n*-butylsilane consumption of individual reactions containing the alcohols (*S*)-**1a**–**e**, initial rates of the individual reactions (~10% conversion), and linear extrapolations estimated from ~10% conversion as visual aids are shown in Figure S27.

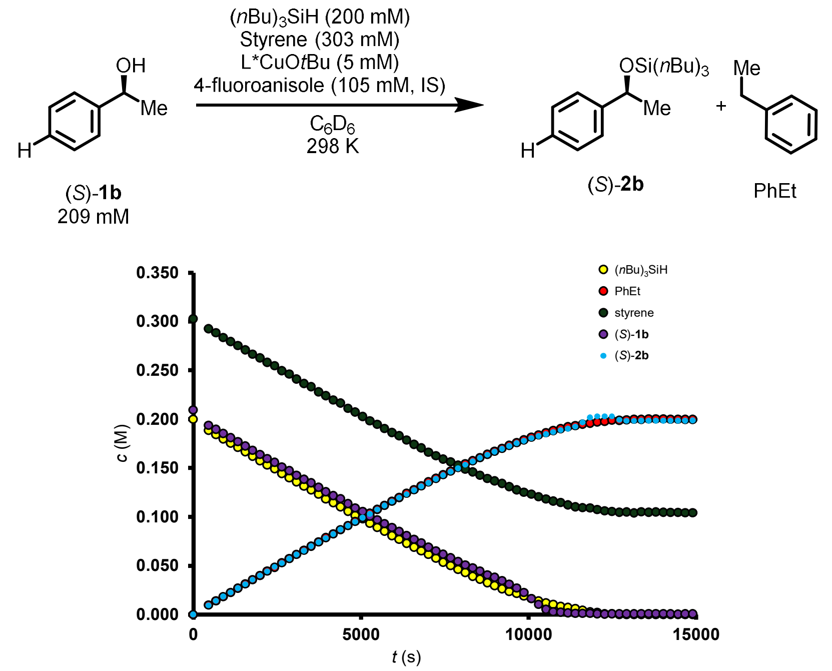

Figure S18. Initial reaction conditions and temporal concentration profiles of reactants and products in the reaction of (*S*)-1-(phenyl)-ethan-1-ol ((*S*)-**1b**). Concentrations of (*S*)-**1b** (from *t* ~ 10000 s) and of (*S*)-**2b** (from *t* ~ 12000 s) show artifacts resulting from peak drift of (*S*)-**1b** and subsequent overlap with (*S*)-**2b** at higher conversions.

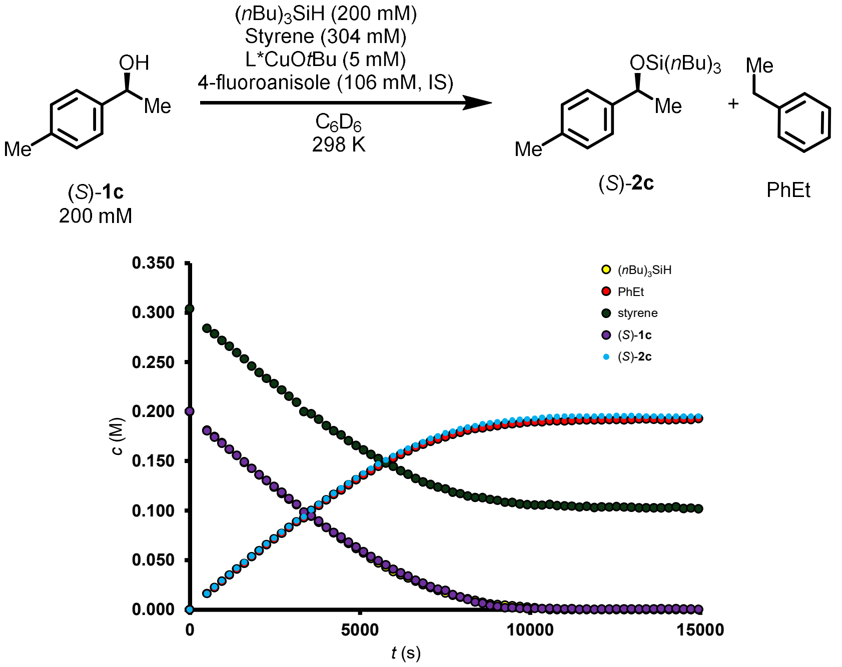

Figure S19. Initial reaction conditions and temporal concentration profiles of reactants and products in the reaction of (*S*)-1-(4-(methyl)phenyl)ethan-1-ol ((*S*)-**1c**).

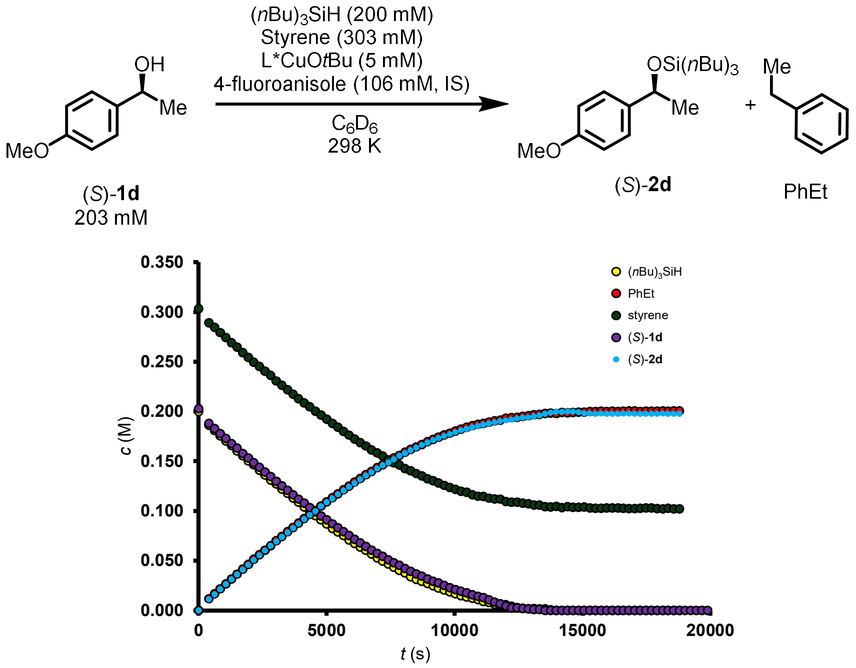

Figure S20. Initial reaction conditions and temporal concentration profiles of reactants and products in the reaction of (*S*)-1-(4-(methoxy)phenyl)ethan-1-ol ((*S*)-**1d**). Concentrations of (*S*)-**1d** (from *t* ~ 10000 s) and of (*S*)-**2d** (from *t* ~ 12000 s) show artifacts resulting from peak drift of (*S*)-**1d** and subsequent overlap with (*S*)-**2d** at higher conversions.

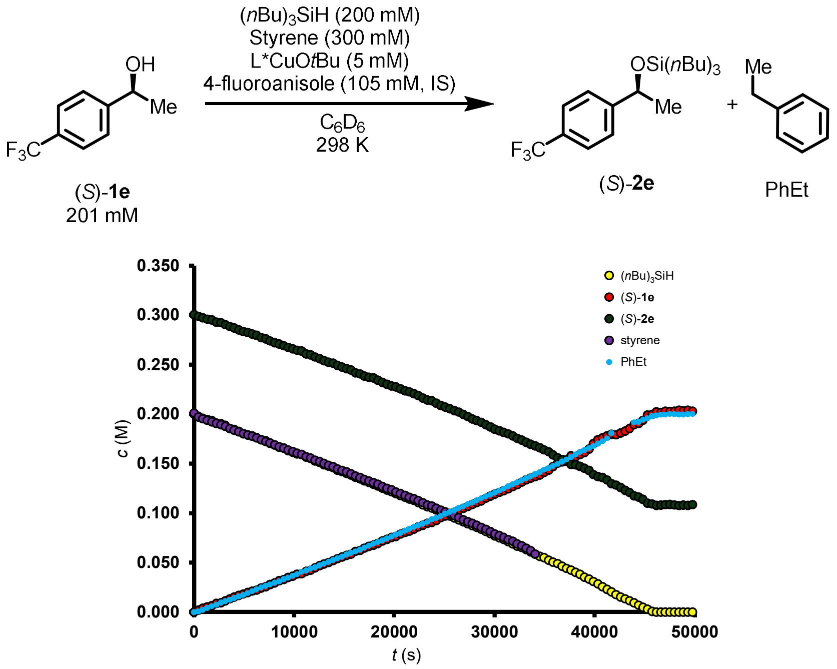

Figure S21. Temporal concentration profiles of reactants and products in the reaction of (*S*)-1-(4-(trifluoromethyl)phenyl)ethan-1-ol ((*S*)-**1e**). Concentrations of (*S*)-**1e** and (*S*)-**2e** are truncated due to unreliable integration resulting from peak drift of (*S*)-**1e** and overlap with (*S*)-**2e** at higher conversions.

4.2 Bigeleisen-Wolfsberg Analysis

For competition reactions where the fractional conversion of all competing species, F_T, is considered, Bigeleisen-Wolfsberg analysis[S5] (Equation S1) was carried out employing a rearranged form of Equation S1 to obtain a relative rate constant k_B/k_A (Equation S2). Total fractional conversion, F_T, substrate ratio, R_A, and initial substrate ratio, R_0, are defined in Equations S3–S5. For competition reactions where the fractional conversion of only one of the competing species, F_L (Equation S6), is considered, an equivalent expression (Equation S7) was employed. In all cases, k_B/k_A was determined by nonlinear regression in Excel Solver.

$$\frac{k_\mathrm{B}}{k_\mathrm{A}} = \frac{\ln\left((1-F_\mathrm{T})\cdot\left(\frac{1+R_0}{1+R_\mathrm{A}}\right)\right)}{\ln\left((1-F_\mathrm{T})\cdot\left(\frac{1+R_0}{1+R_\mathrm{A}}\right)\cdot\left(\frac{R_\mathrm{A}}{R_0}\right)\right)} \tag{S1}$$

$$F_\mathrm{T} = 1-\left(\frac{1+R_\mathrm{A}}{1+R_0}\right)\cdot\left(\frac{R_\mathrm{A}}{R_0}\right)^{\left(\frac{\frac{k_\mathrm{B}}{k_\mathrm{A}}}{1-\frac{k_\mathrm{B}}{k_\mathrm{A}}}\right)} \tag{S2}$$

$$F_\mathrm{T} = 1-\left(\frac{[\mathrm{A}]_t+[\mathrm{B}]_t}{[\mathrm{A}]_0+[\mathrm{B}]_0}\right) \tag{S3}$$

$$R_\mathrm{A} = \left(\frac{[\mathrm{B}]_t}{[\mathrm{A}]_t}\right) \tag{S4}$$

$$R_0 = \left(\frac{[\mathrm{B}]_0}{[\mathrm{A}]_0}\right) \tag{S5}$$

$$F_\mathrm{L} = 1-\left(\frac{[\mathrm{A}]_t}{[\mathrm{A}]_0}\right) \tag{S6}$$

$$\frac{R_\mathrm{A}}{R_0} = (1-F_\mathrm{L})^{\left(\frac{k_\mathrm{B}}{k_\mathrm{A}}\right)-1} \tag{S7}$$

4.3 Intermolecular Competition Reactions of (*S*)-1a–e

Reaction mixtures containing the respective enantiopure (*S*)-**1** alcohols, $(n\mathrm{Bu})_3\mathrm{SiH}$, styrene, and 4-fluoroanisole in C_6D_6 were assembled in J-Young NMR tube and monitored according to general procedure GP1. Bigeleisen-Wolfsberg analyses[S5] were carried out using Equation S2. Initial conditions and Bigeleisen-Wolfsberg plots of each reaction are shown in Figures S22–S25.

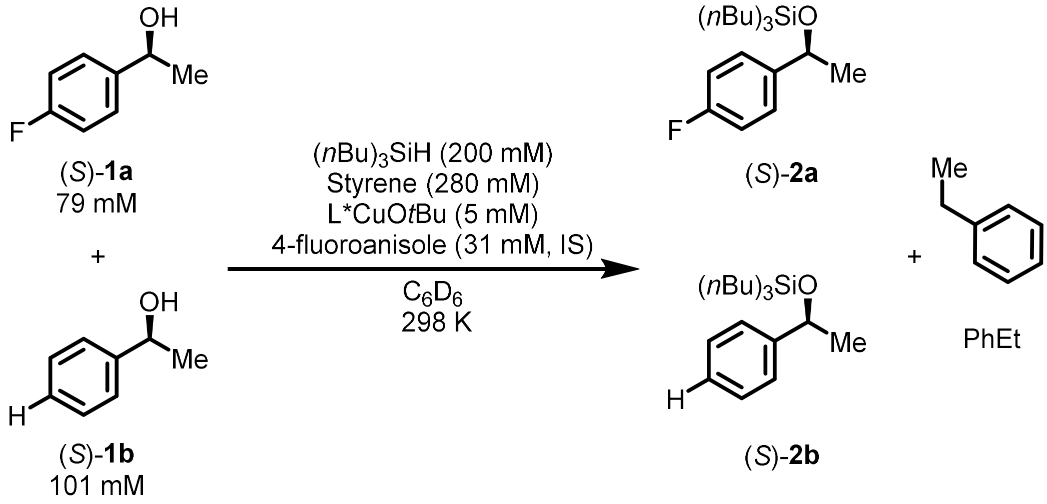

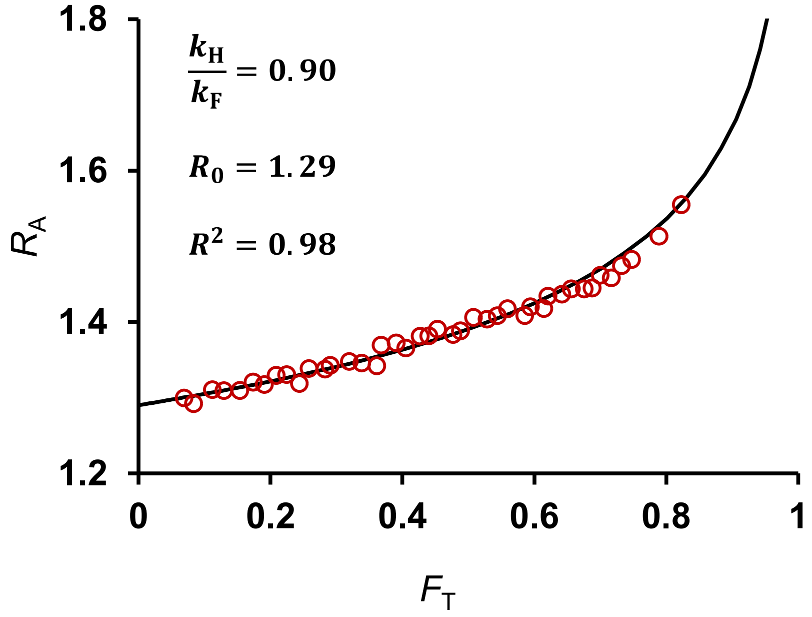

Figure S22. Initial reaction conditions and Bigeleisen-Wolfsberg[S5] plot of the competition reaction between (*S*)-**1a** and (*S*)-**1b**. The relative rate constant, initial substrate ratio R_0, and R^2 coefficient of determination from nonlinear fitting to Equation S2 are shown.

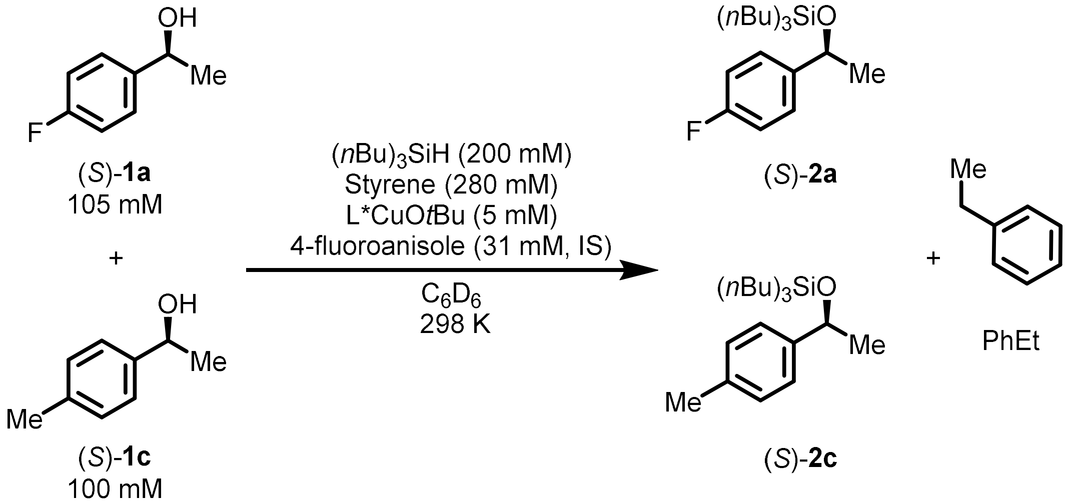

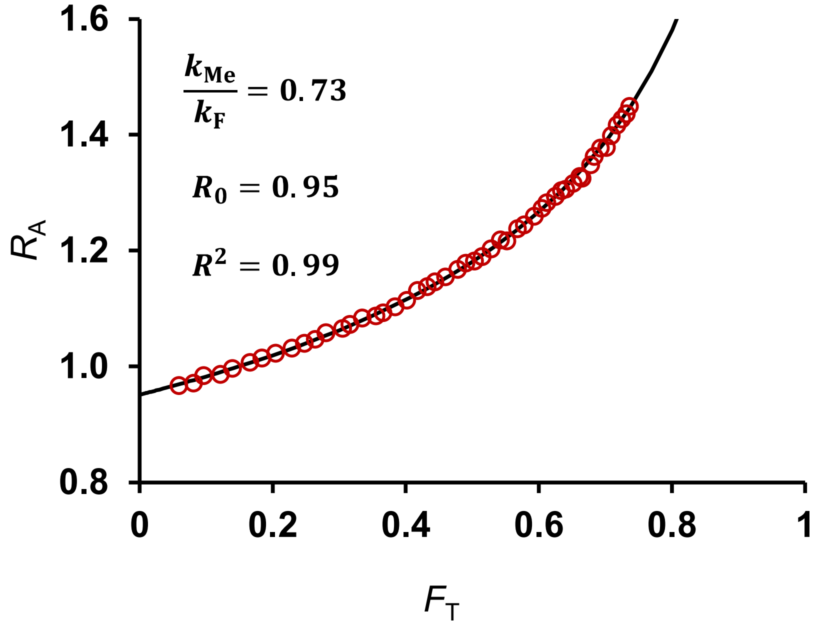

Figure S23. Initial reaction conditions and Bigeleisen-Wolfsberg[S5] plot of the competition reaction between (*S*)-**1a** and (*S*)-**1c**. The relative rate constant, initial substrate ratio R_0, and R^2 coefficient of determination from nonlinear fitting to Equation S2 are shown.

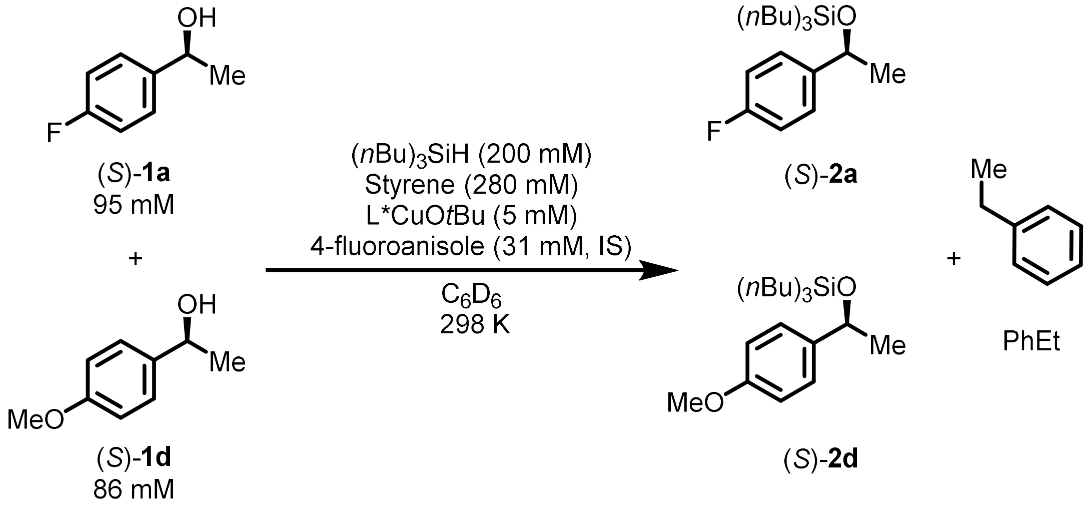

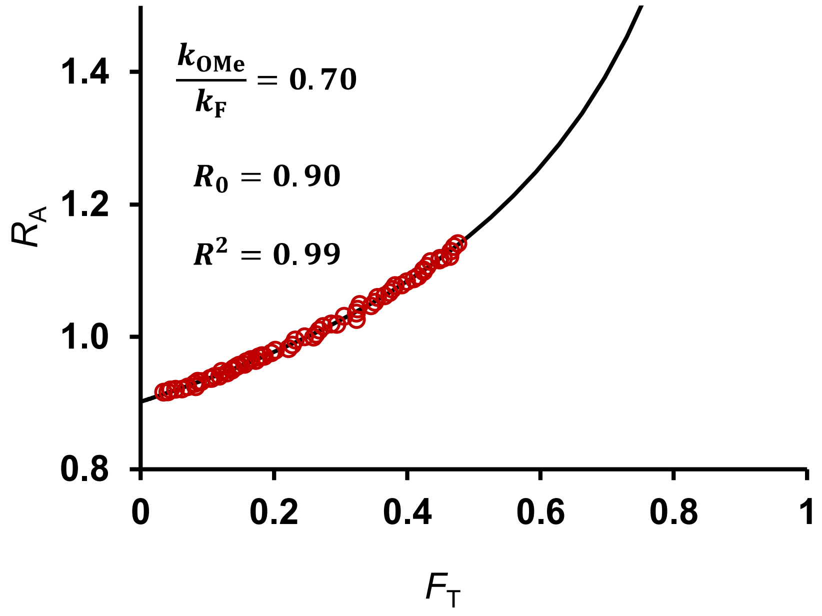

Figure S24. Initial reaction conditions and Bigeleisen-Wolfsberg[S5] plot of the competition reaction between (*S*)-**1a** and (*S*)-**1d**. The relative rate constant, initial substrate ratio R_0, and R^2 coefficient of determination from nonlinear fitting to Equation S2 are shown.

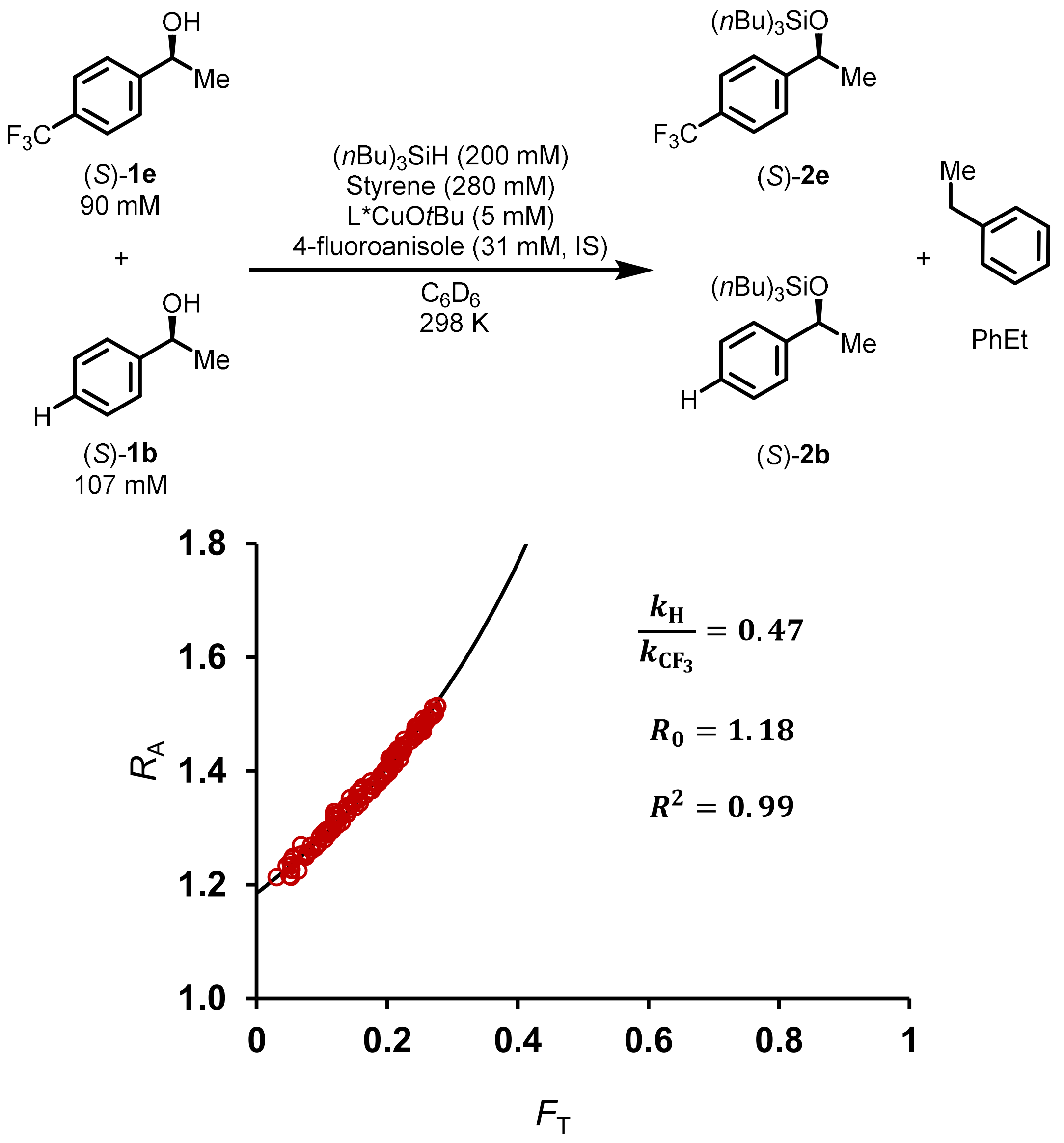

Figure S25. Initial reaction conditions and Bigeleisen-Wolfsberg[S5] plot of the competition reaction between (*S*)-**1e** and (*S*)-**1b**. The relative rate constant, initial substrate ratio R_0, and R^2 coefficient of determination from nonlinear fitting to Equation S2 are shown.

4.4 Hammett Correlation

A Hammett correlation of standard Hammett substituent constants[S6] and the relative k_1 values obtained from the competition reactions described in Section 4.3 is depicted in Figure S26 (see Section 8.1 for details on constants such as k_1, K_i, etc.). A positive ρ = +0.56 value is obtained, indicating a small dependency of reactivity on aromatic substituents.

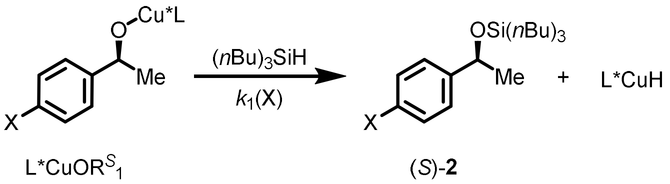

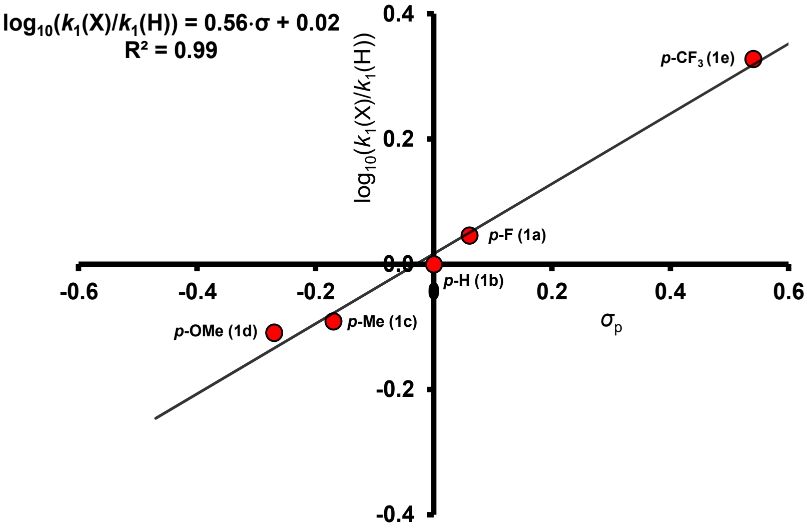

Figure S26. Hammett correlation employing relative rate constants from Section 4.3 (Intermolecular Competition Reactions of (*S*)-**1a**–**e**) and standard Hammett substituent parameters σ[S6]. σ_p(F) = 0.06, σ_p(H) = 0, σ_p(Me) = −0.17, σ_p(OMe) = −0.27, $\sigma_p(CF_3)$ = 0.54. The reaction depicted above the plot represents the (*S*)-**2** determining elementary step within the catalytic manifold.

The opposite trend is observed when reactions are carried out for the alcohols (*S*)-**1a**–**e** independently, where electron-withdrawing groups in (*S*)-**1a** result in slower reaction rates. Macrokinetic initial rates of $(nBu)_3SiH$ consumption, v_0, were obtained and normalized relative to the initial rate of the reaction of (*S*)-1-(phenyl)-ethan-1-ol ((*S*)-**1b**), Figure S27. The obtained relative rate v_0/v_H value was plotted against standard Hammett substituent constants,[S6] Figure S28. The resulting plot is overall more scattered and results in a larger, negative ρ = −1.2 value, indicating that inhibition by (*S*)-**1** derivatives is more sensitive to the nature of its substituents than the dehydrogenative silylation elementary step. At early reaction stages, it is valid to apply the steady-state approximation described in Equation S22 (see Section 8.1 for derivation and details) to deconvolute v_0 into two principal components, k_1 and K_i. The

resulting Hammett correlation of K_i results in a positive slope of ρ = +1.7 (Figure S29), consistent with K_i being more sensitive than k_1 to substituent effects in (*S*)-**1** derivatives.

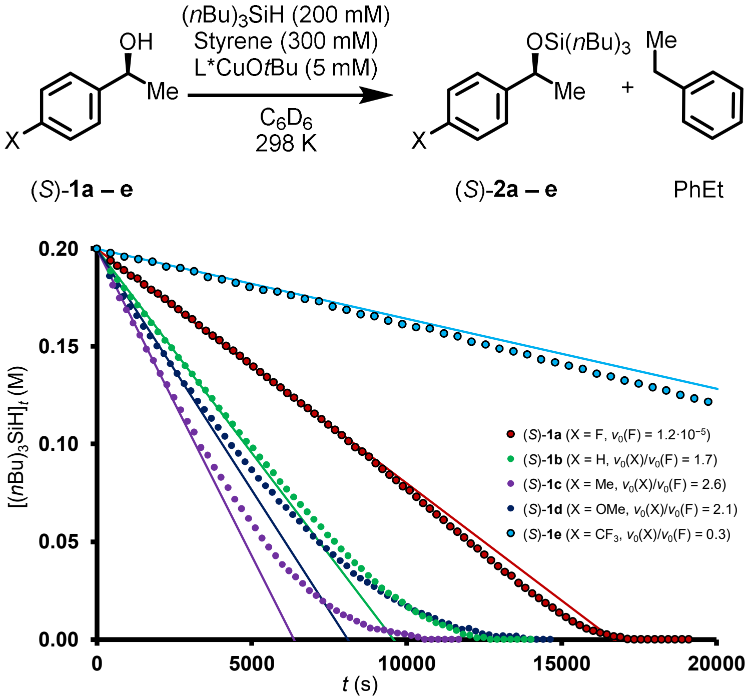

Figure S27. Temporal concentration profiles of (nBu)$_3$SiH in reactions of various (*S*)-**1** derivatives. Initial rates v_0 and linear extrapolations (provided as visual aids) are estimated from approximately 10% conversion.

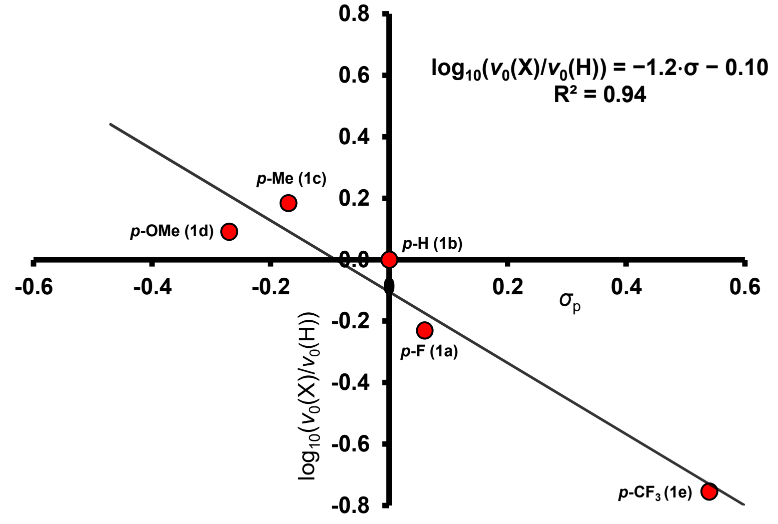

Figure S28. Hammett correlation employing relative initial rates v_0 from Figure S27 (Individual Reactions of (*S*)-**1a–e**) and standard Hammett substituent parameters σ[S6]. σ_p(F) = 0.06, σ_p(H) = 0, σ_p(Me) = −0.17, σ_p(OMe) = −0.27, σ_p(CF_3) = 0.54.

The joint role of K_i and k_1 in determining macrokinetic behavior, $v_0 \propto k_1/K_i$, is further reinforced by the obtained numerical values of ρ, as it is positive for the individual LFERs of both k_1 and K_i, but overall negative for v_0 due to the role of K_i as an inhibitory equilibrium and its higher sensitivity to substituents. It is also shown that the apparent scatter in v_0 results from its K_i component, whereas k_1 is better described by its LFER. The apparent discrepancy may result from higher order effects which a simpler LFER cannot adequately describe, as K_i is an equilibrium which contains two distinct species with identical substituents, the (*S*)-**1** derivative its alkoxide L*CuOR, respectively an H-bond donor and acceptor, which could lead to a mismatch in resonance and field contributions influencing the acceptor ability of L*CuOR and the donor ability of (*S*)-**1**.

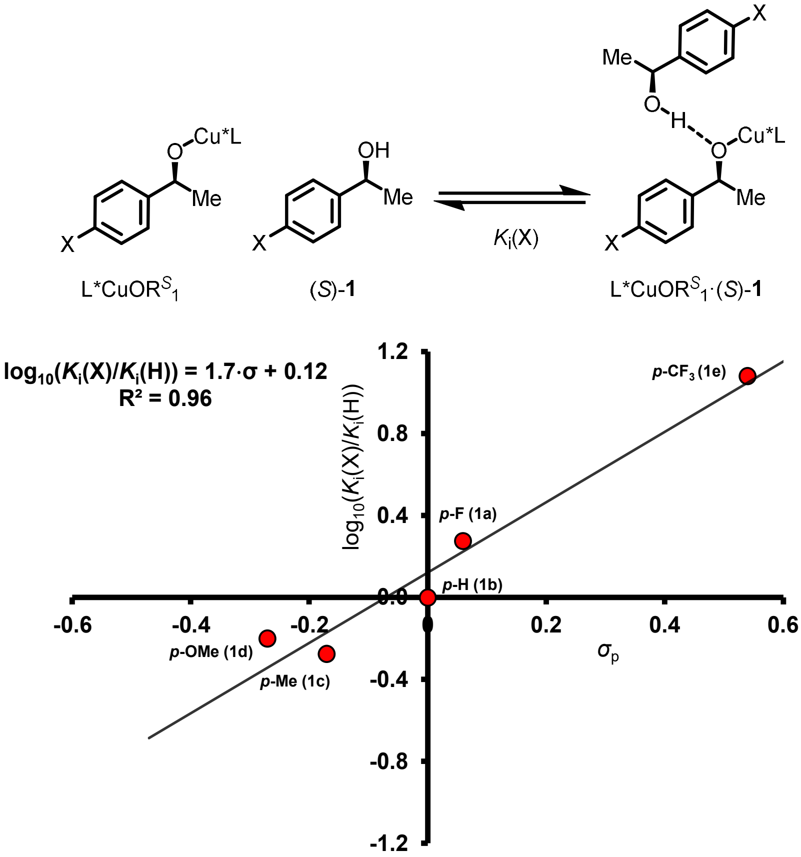

Figure S29. Hammett correlation employing relative equilibrium constants K_i obtained from Equation S22 (see Section 8.1 for details) and standard Hammett substituent parameters σ[S6]. σ_p(F) = 0.06, σ_p(H) = 0, σ_p(Me) = −0.17, σ_p(OMe) = −0.27, $\sigma_p(CF_3)$ = 0.54. The equilibrium depicted above the plot represents the proposed elementary step which inhibits turnover within the catalytic manifold.

The obtained K_i and k_1 values were validated via the minimal kinetic model employed in this work (Figure S55, see Section 8.1 for details). Additionally, estimated values for k_3, which

describe the deprotonation of alcohols (*S*)-**1a**−**e** by the copper species L*CuCH(Me)Ph were obtained from fitting the model to the respective dataset. The resulting LFER has a positive slope ρ = +0.96 (Figure S30), which indicates stronger sensitivity towards substituents than is detected with k_1, further elucidating the alcohol dependence of the departure from pseudo zeroth order conditions in Figure S27.

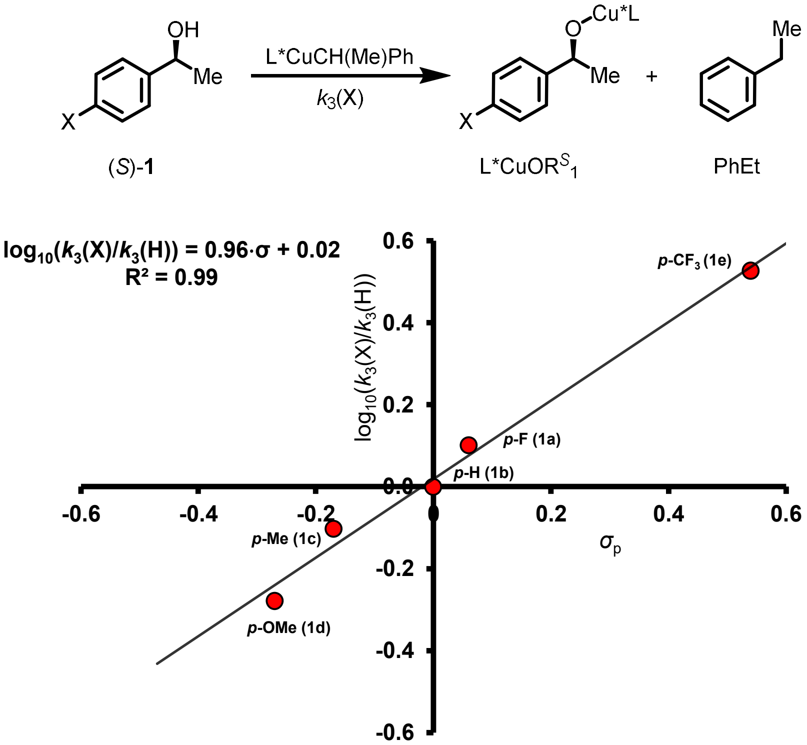

Figure S30. Hammett correlation employing relative deprotonation rate constants k_3 obtained from the kinetic model (see Section 8.1 for details) and standard Hammett substituent parameters σ[S6]. σ_p(F) = 0.06, σ_p(H) = 0, σ_p(Me) = −0.17, σ_p(OMe) = −0.27, σ_p(CF$_3$) = 0.54. The reaction depicted above the plot represents the elementary step within the catalytic manifold which irreversibly generates ethylbenzene.

A Swain-Lupton[S6] analysis for v_0 where σ_{SL} parameters are constructed from the respective standard *F* and *R* substituent parameters and weighing factors *f* and *r* (Equation S8) results in a linear correlation, Figure S31. The optimal value for *f* was determined via nonlinear regression using Excel Solver. Standard σ_p values for all substituents except for H ($\Delta\sigma_p$ = 0.03) are obtained with *f* = 1 and *r* = 1. In this analysis, *r* has been defined as (2−*f*) so that *f* + *r* = 2

$$\sigma_{SL} = f \cdot F + r \cdot R = f \cdot F + ((2 - f) \cdot R) \tag{S8}$$

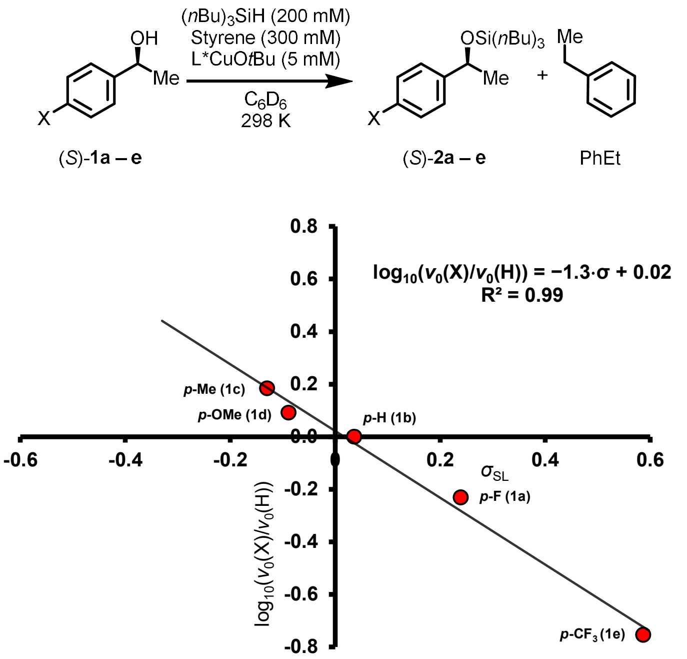

Figure S31. Hammett correlation employing relative rate constants from Figure S27 (Individual Reactions of (*S*)-**1a**–**e**) and substituent constants σ_{SL} constructed from weighted Swain-Lupton parameters (Equation S8). f = 1.21, r = 0.79, σ_{SL}(F) = 0.24, σ_{SL}(H) = 0.04, σ_{SL}(Me) = −0.13, σ_{SL}(OMe) = −0.09, σ_{SL}(CF_3) = 0.59.

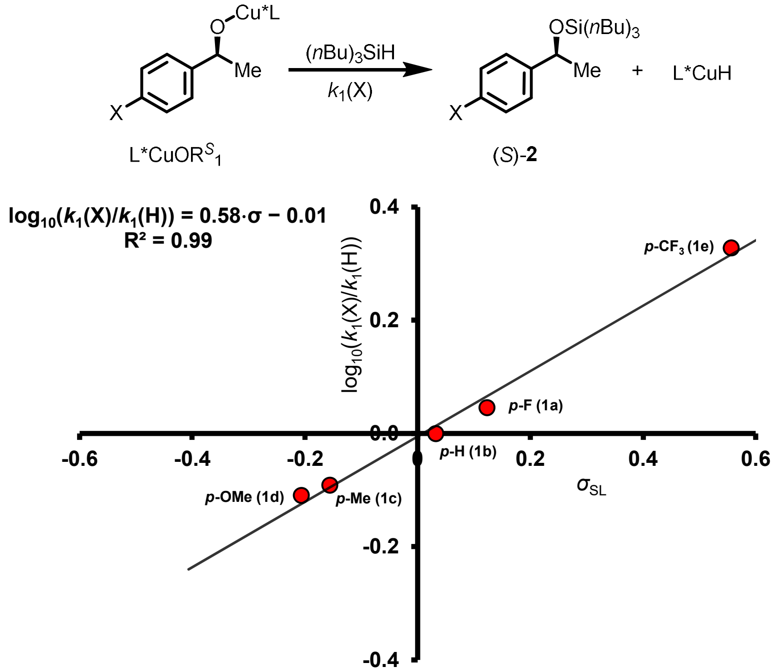

Figure S32. Hammett correlation employing relative rate constants from Section 4.3 (Intermolecular Competition Reactions of (*S*)-**1a**–**e**) and substituent constants σ_{SL} constructed from weighted Swain-Lupton parameters (Equation S8). f = 1.08, r = 0.92, σ_{SL}(F) = 0.12, σ_{SL}(H) = 0.03, σ_{SL}(Me) = −0.16, σ_{SL}(OMe) = −0.21, σ_{SL}(CF_3) = 0.56. The reaction depicted above the plot represents the (*S*)-**2** determining elementary step within the catalytic manifold.

The fitted value f = 1.21 (Figure S31) indicates that the overall field effects governing the macrokinetic regime of (S)-**1** alcohols are underestimated by standard σ_p values. In contrast, f = 1.08 was fitted for k_1 values obtained from the competition reactions described in Section 4.3 (Figure S32), indicating that while field effects are also underestimated for the dehydrogenative silylation elementary step, the step is less sensitive to field effects than the overall reaction.

These results indicate that substrate inhibition is strongly dominated by field effects, consistent with a H-bonding mode of inhibition. This conclusion was further validated by an additional Swain-Lupton analysis for K_i (f = 1.18, Figure S33). In contrast, the corresponding analysis for the deprotonation of alcohols (S)-**1a**−**e** by the copper species L*CuCH(Me)Ph, described by k_3, (f = 1.01, Figure S34) resulted in no significant mismatch between standard Hammett substituent parameters σ_p and the resulting Swain-Lupton parameters σ_{SL}.

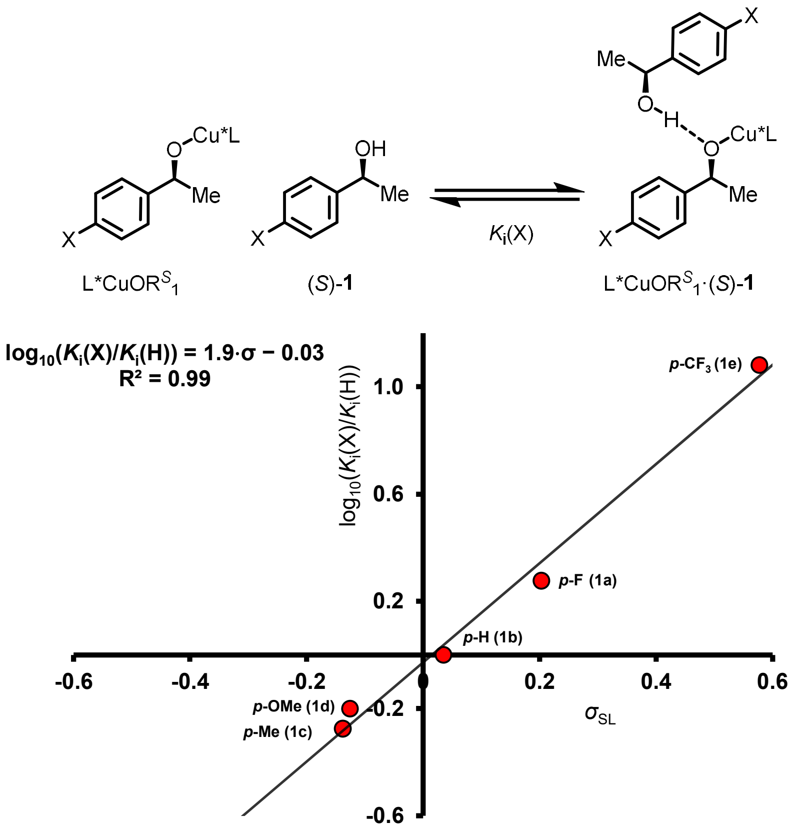

Figure S33. Hammett correlation employing relative equilibrium constants K_i obtained from Equation S22 (see Section 8.1 for details) and substituent constants σ_{SL} constructed from weighted Swain-Lupton parameters (Equation S8). f = 1.18, r = 0.82, σ_{SL}(F) = 0.20, σ_{SL}(H) = 0.04, σ_{SL}(Me) = −0.14, σ_{SL}(OMe) = −0.13, $\sigma_{SL}(CF_3)$ = 0.58. The equilibrium depicted above the plot represents the proposed elementary step which inhibits turnover within the catalytic manifold.

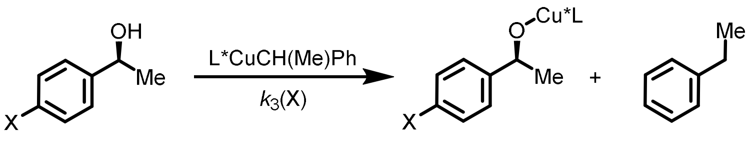

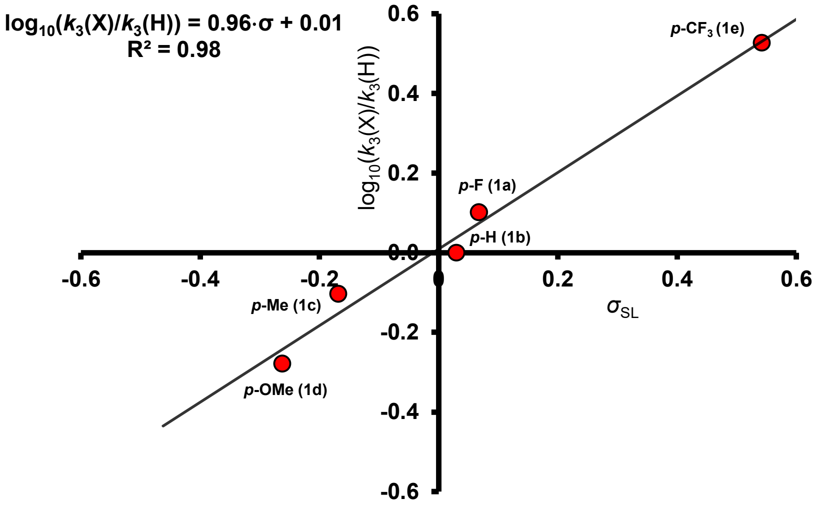

Figure S34. Hammett correlation employing relative deprotonation rate constants k_3 obtained from the kinetic model (see Section 8.1 for details) and substituent constants σ_{SL} constructed from weighted Swain-Lupton parameters (Equation S8). f = 1.01, r = 0.99, σ_{SL}(F) = 0.07, σ_{SL}(H) = 0.03, σ_{SL}(Me) = −0.17, σ_{SL}(OMe) = −0.26, $\sigma_{SL}(CF_3)$ = 0.54. The reaction depicted above the plot represents the elementary step within the catalytic manifold which irreversibly generates ethylbenzene.

4.5 Intermolecular Competition Reaction of (*S*)-1a vs (*R*)-1a-C_D, (*R*)-1a vs (*S*)-1a-C_D, and (*S*)-1a vs (*S*)-1a-C_D

Reaction mixtures containing the respective enantiopure (*S*)-**1a**, (*R*)-**1a**, (*S*)-**1a-C_D**, and (*R*)-**1a-C_D** alcohols, $(nBu)_3SiH$, styrene, and 4-fluoroanisole in C_6D_6 were assembled in J-Young NMR tube and monitored according to general procedure GP1. Bigeleisen-Wolfsberg analyses[S5] were carried out using Equation S7. Initial conditions and Bigeleisen-Wolfsberg plots of each reaction are shown in Figures S35−S38.

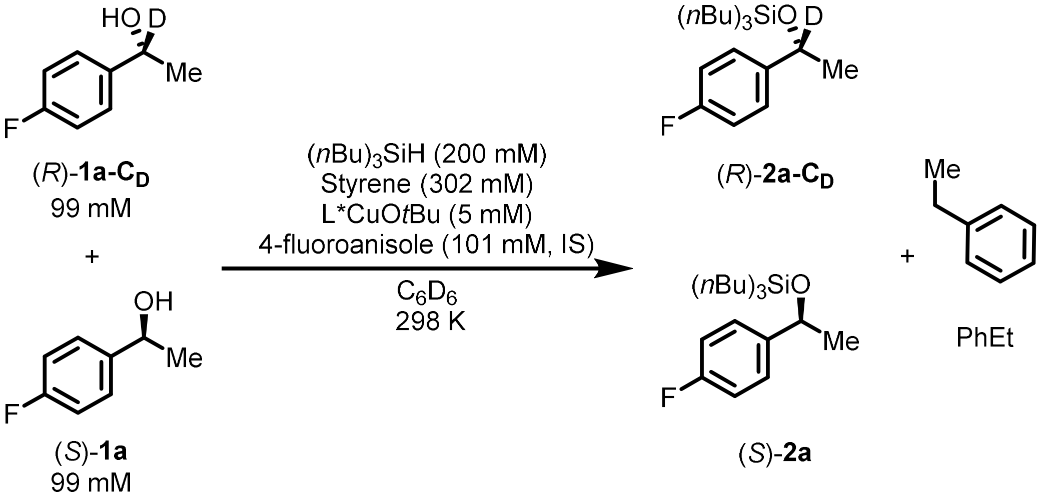

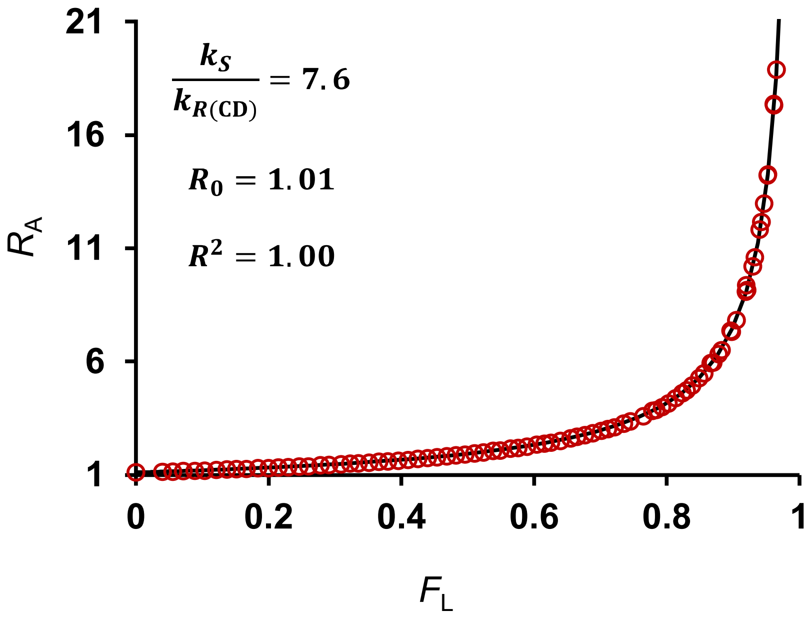

Figure S35. Initial reaction conditions and Bigeleisen-Wolfsberg[S5] plot of the competition reaction between (*S*)-**1a** and (*R*)-**1a-C_D**. The relative rate constant, initial substrate ratio R_0, and R^2 coefficient of determination from nonlinear fitting to Equation S7 are shown.

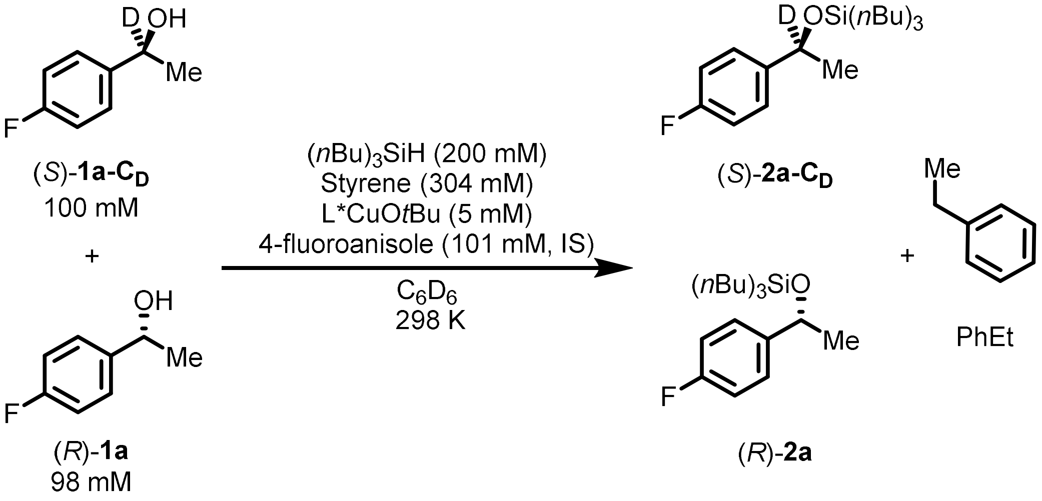

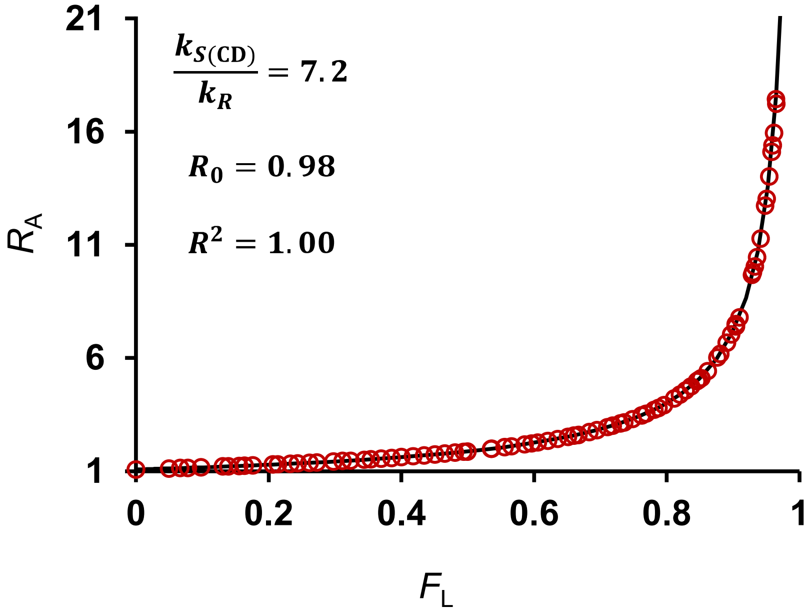

Figure S36. Initial reaction conditions and Bigeleisen-Wolfsberg[S5] plot of the competition reaction between (R)-**1a** and (S)-**1a-C$_D$**. The relative rate constant, initial substrate ratio R_0, and R^2 coefficient of determination from nonlinear fitting to Equation S7 are shown.

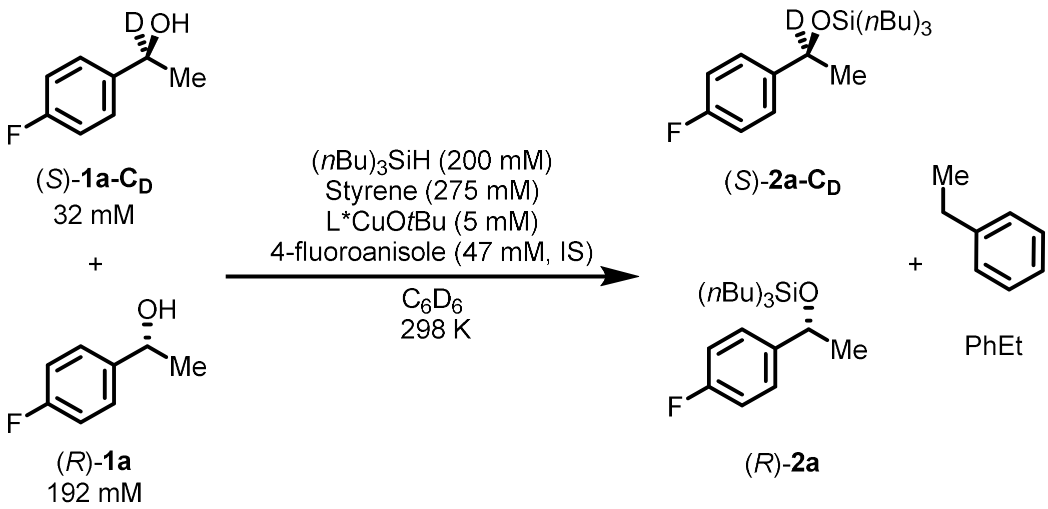

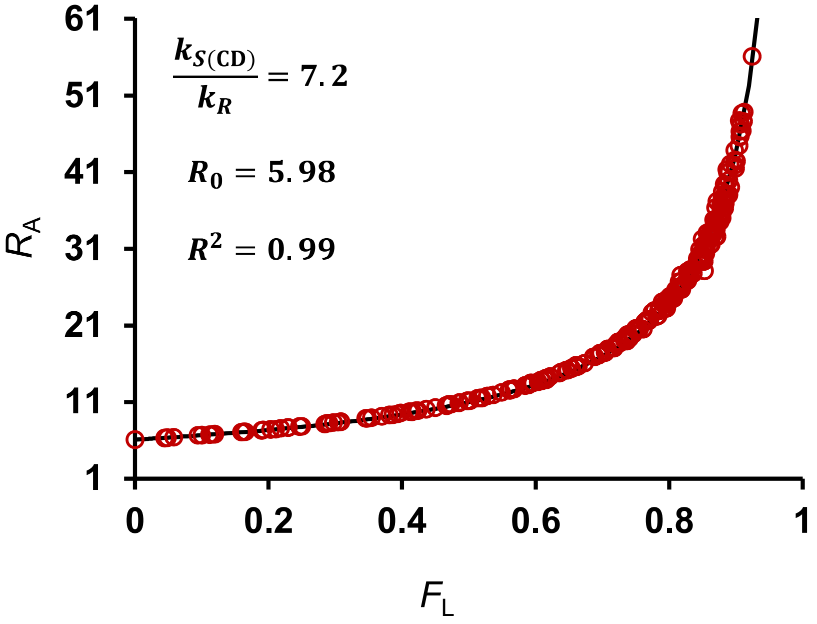

Figure S37. Initial reaction conditions and Bigeleisen-Wolfsberg[S5] plot of the competition reaction between (R)-**1a** and (S)-**1a-C$_D$** under alternative initial substrate ratios. The relative rate constant, initial substrate ratio R_0, and R^2 coefficient of determination from nonlinear fitting to Equation S7 are shown.

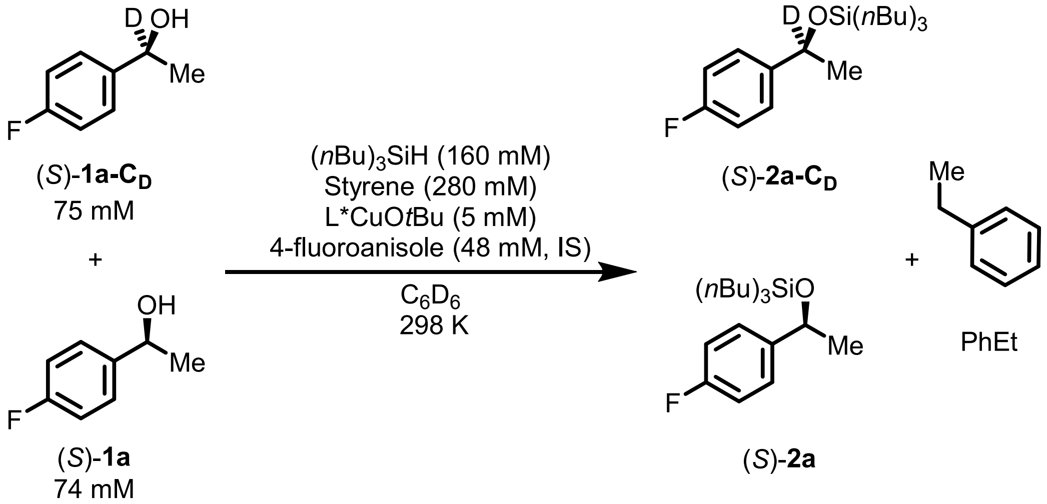

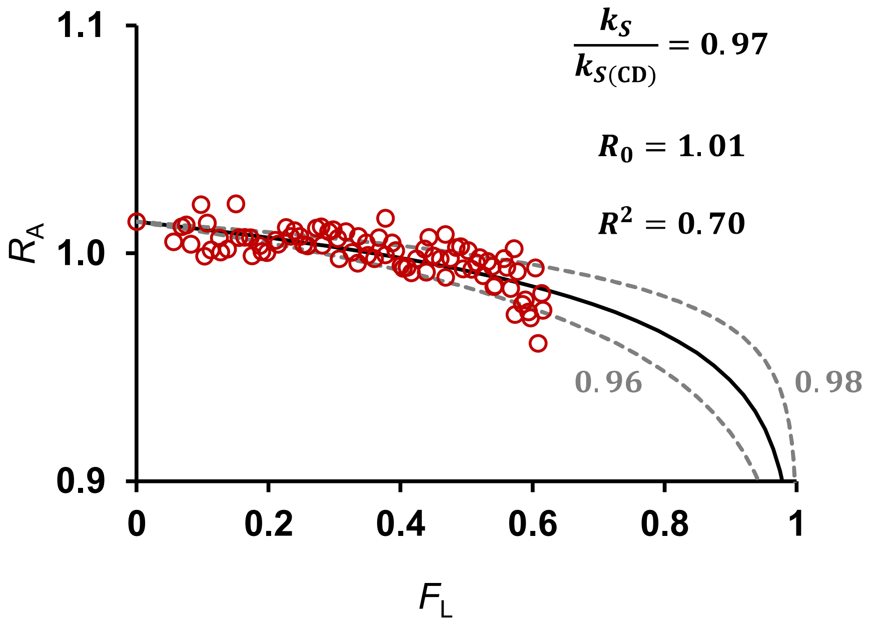

Figure S38. Initial reaction conditions and Bigeleisen-Wolfsberg[S5] plot of the competition reaction between (*S*)-**1a** and (*S*)-**1a-C$_D$**. The relative rate constant, initial substrate ratio R_0, and R^2 coefficient of determination from nonlinear fitting to Equation S7 are shown. Grey dotted lines are respectively simulations of $k_S/k_{S(CD)} = 0.98$ (upper) and $k_S/k_{S(CD)} = 0.96$ (lower).

To extract a relative rate constant between (*S*)-**1a** and (*R*)-**1a**, the respective kinetic isotope effects of (*R*)-**1a-C$_D$** (Figure S35) and of (*S*)-**1a-C$_D$** (Figure S36, Figure S37) must be considered. If either KIE value is known, values for k_S/k_R and the KIE value of the other enantiomer can be obtained (Equation S12)

$$\frac{k_{S(\mathrm{CD})}}{k_R} = \frac{k_S}{k_R \cdot KIE_{\mathrm{H/D}(S)}} \tag{S9}$$

$$\frac{k_S}{k_{R(\mathrm{CD})}} = \frac{k_S \cdot KIE_{\mathrm{H/D}(R)}}{k_R} \tag{S10}$$

$$\frac{k_S}{k_{S(\mathrm{CD})}} = KIE_{\mathrm{H/D}(S)} \tag{S11}$$

$$\frac{k_S}{k_R} = \frac{k_{S(\mathrm{CD})}}{k_R} \cdot KIE_{H/D(S)} = \frac{k_S}{k_{R(\mathrm{CD})} \cdot KIE_{\mathrm{H/D}(R)}} \tag{S12}$$

The respective relative rates $k_{S(CD)}/k_R$ = 7.2, $k_S/k_{R(CD)}$ = 7.6, and $KIE_{H/D(S)}$ = 0.97 (Figure S38) result in k_S/k_R = 7.4 and $KIE_{H/D(R)}$ = 0.98. When assuming instead that the secondary KIE is enantiomer independent, $KIE_{H/D(S)} = KIE_{H/D(R)}$, the measured value of $KIE_{H/D(S)}$ is not required to reach a solution, and the values k_S/k_R = 7.4 and *KIE* = 0.97 are obtained, indicating that either approach is valid within experimental accuracy. For simplicity, the values k_S/k_R = 7.4 and $KIE_{H/D(S)} = KIE_{H/D(R)}$ = 0.97 were used throughout this work.

Similar values for k_S/k_R are obtained when Bigeleisen-Wolfsberg analyses[S5] are carried out with individual concentrations of (*S*)-**1a** and (*R*)-**1a** obtained from integration of their resolved ^{19}F NMR signals (k_S/k_R = 7.4, Figure S39, see Figure S13 for time-concentration plot). Similarly, when limiting conversion with substoichiometric amounts of (nBu)$_3$SiH (0.108 M, k_S/k_R = 7.3, Figure S40), and when limiting conversion with substoichiometric (nBu)$_3$SiH and increasing the ligand loading (0.108 M (nBu)$_3$SiH, 6 mol% ligand with 0.001 M (*R*,*R*)-Ph-BPE added to 0.005 M [Cu]$_0$, k_S/k_R = 7.4, Figure S41), values of similar magnitude are obtained. No significant change in the overall kinetics or in the value of k_S/k_R was observed with increased loadings of (*R*,*R*)-Ph-BPE.

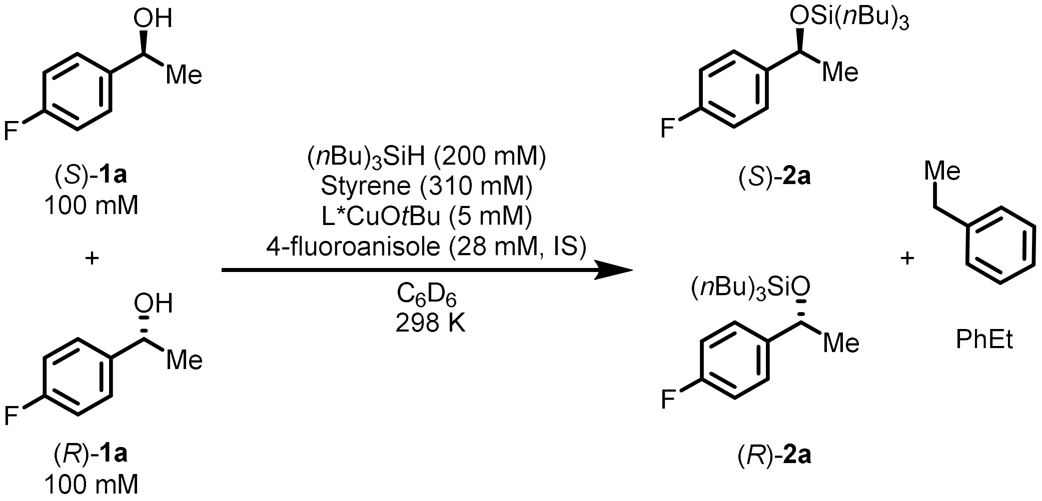

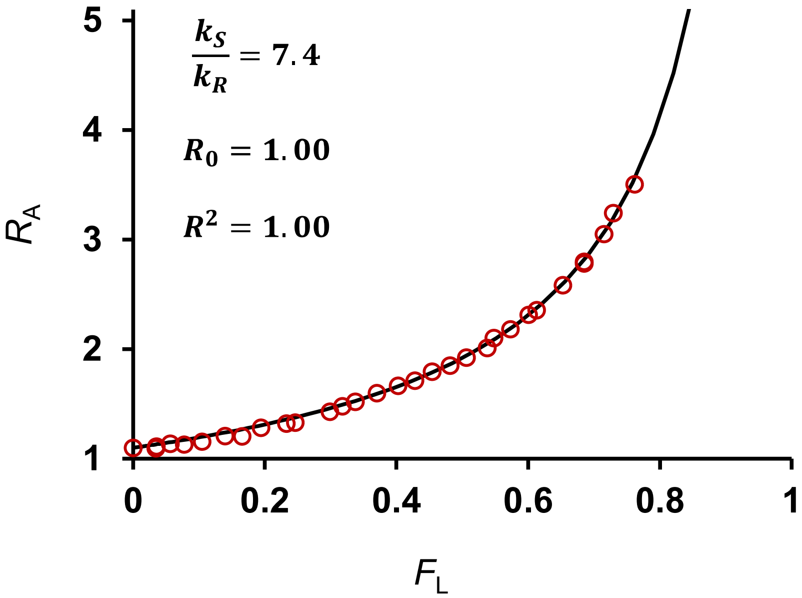

Figure S39. Initial reaction conditions and Bigeleisen-Wolfsberg[S5] plot of the competition reaction between (*R*)-**1a** and (*S*)-**1a** from a racemic mixture of **1a**. Individual concentrations of (*R*)-**1a** and (*S*)-**1a** were obtained from their individually resolved ^{19}F signals (see Figure S13 for time-concentration profile). The relative rate constant, initial substrate ratio R_0, and R^2 coefficient of determination from nonlinear fitting to Equation S7 are shown.

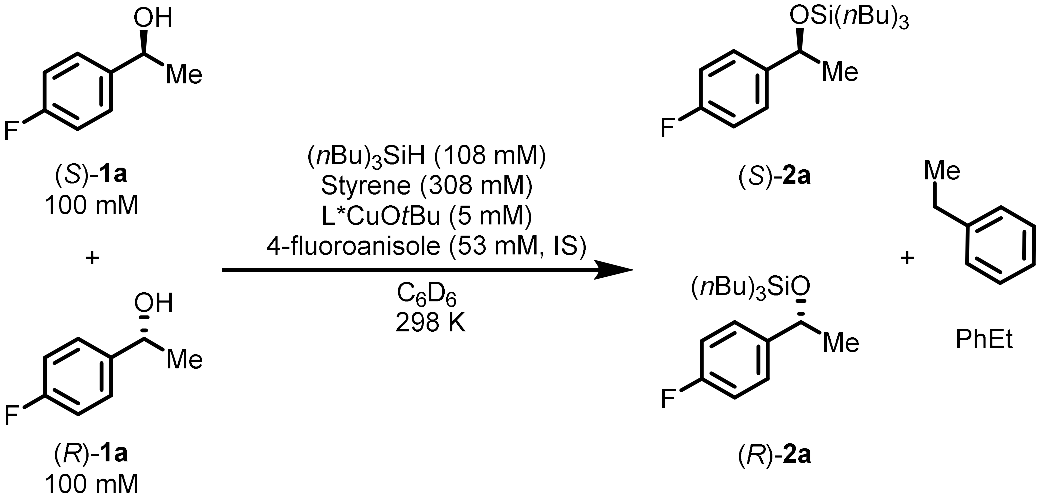

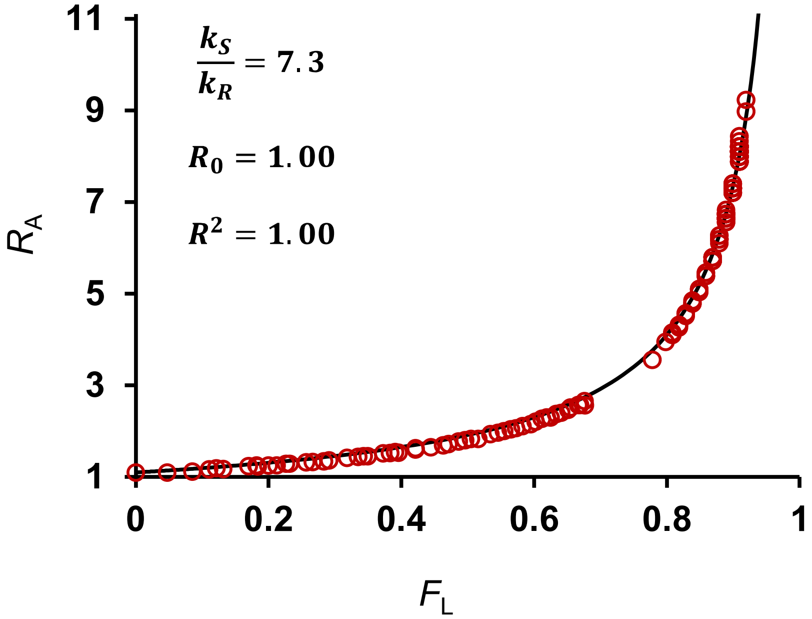

Figure S40. Initial reaction conditions and Bigeleisen-Wolfsberg[S5] plot of the competition reaction between (*R*)-**1a** and (*S*)-**1a** from a racemic mixture of **1a** with substoichiometric amounts of (nBu)$_3$SiH. Individual concentrations of (*R*)-**1a** and (*S*)-**1a** were obtained from their individually resolved ^{19}F signals (see Figure S13 for a representative example). The relative rate constant, initial substrate ratio R_0, and R^2 coefficient of determination from nonlinear fitting to Equation S7 are shown.

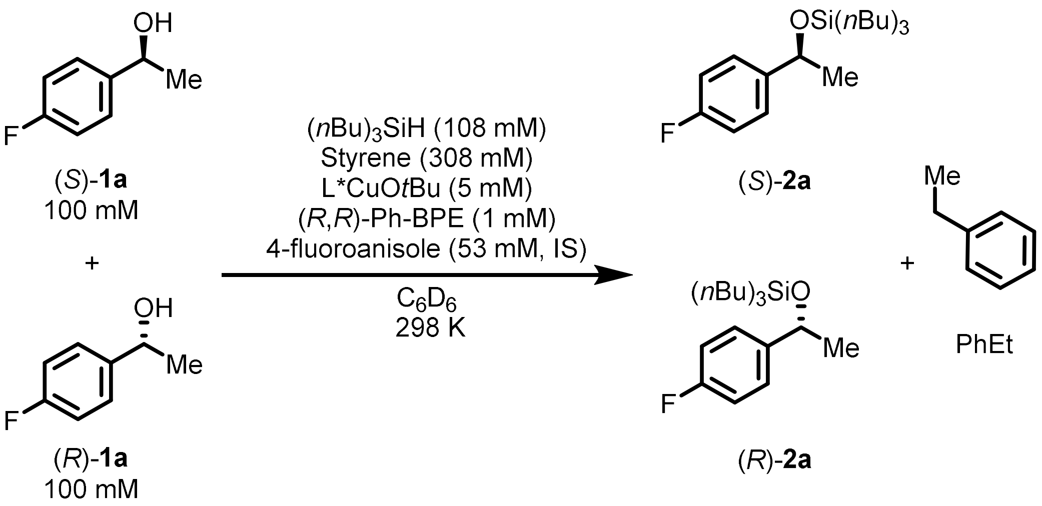

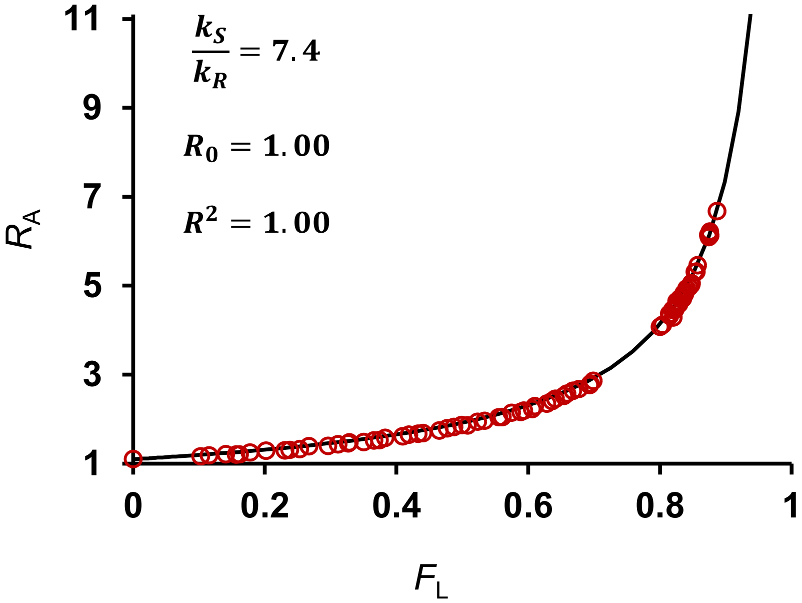

Figure S41. Initial reaction conditions and Bigeleisen-Wolfsberg[S5] plot of the competition reaction between (*R*)-**1a** and (*S*)-**1a** from a racemic mixture of **1a** with substoichiometric amounts of (nBu)$_3$SiH and added (*R*,*R*)-Ph-BPE. Individual concentrations of (*R*)-**1a** and (*S*)-**1a** were obtained from their individually resolved ^{19}F signals (see Figure S13 for a representative example). The relative rate constant, initial substrate ratio R_0, and R^2 coefficient of determination from nonlinear fitting to Equation S7 are shown.

4.6 Reaction of (*S*)-1a-O$_D$

A reaction mixture of the enantiopure alcohol (*S*)-1-(4-fluorophenyl)-ethan-1-ol-*d* ((*S*)-**1a-O$_D$**), (nBu)$_3$SiH, styrene, and 4-fluoroanisole in C_6D_6 was assembled in a J-Young NMR tube according to general procedure GP1. The reaction was monitored at 298 K. Initial conditions and the temporal concentration profile are shown in Figure S42. A temporal concentration profile comparison of tri-*n*-butylsilane consumption of individual reactions of (*S*)-**1a** and (*S*)-**1a-O$_D$** is shown in Figure S43.

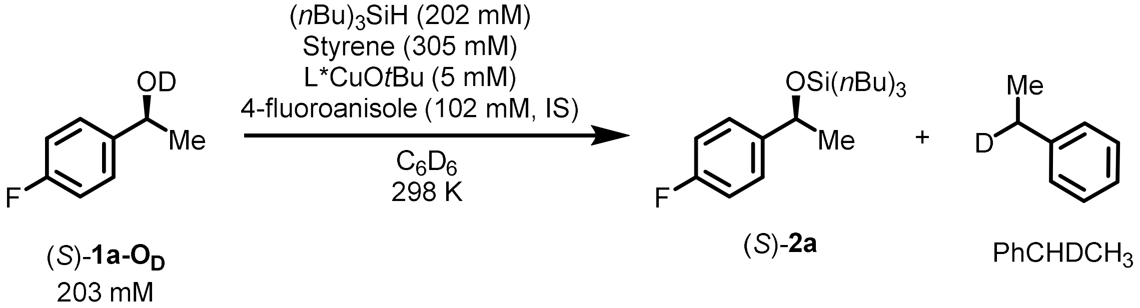

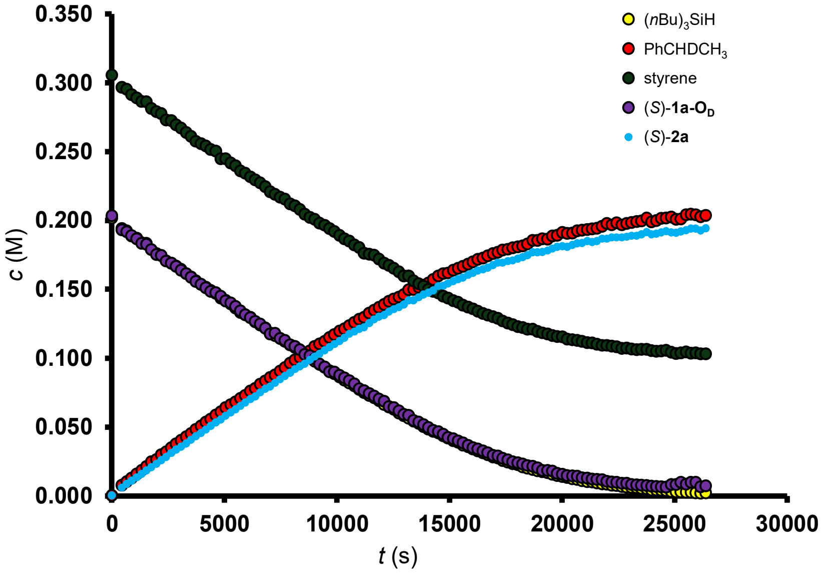

Figure S42. Initial reaction conditions and temporal concentration profiles of reactants and products in the reaction of (*S*)-1-(4-(fluoro)phenyl)ethan-1-ol-*d* ((*S*)-**1a-O$_D$**). Concentrations of (*S*)-**1a-O$_D$** (from t ~ 25000 s) show artifacts resulting from peak drift of (*S*)-**1a-O$_D$** and subsequent overlap with (*S*)-**2a** at higher conversions.

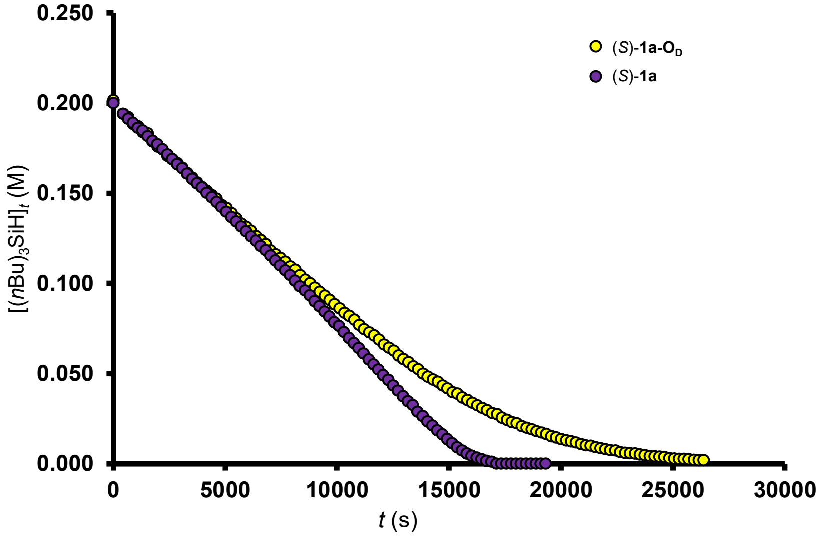

Figure S43. Comparison of temporal concentration profiles of $(n\text{Bu})_3\text{SiH}$ in reactions of (*S*)-**1a**-**O$_D$** and of (*S*)-**1a** under otherwise identical conditions.

4.7 Intermolecular Competition Reaction of (*S*)-1a vs (*S*)-1a-O$_D$ and Kinetic Isotope Effect

A reaction mixture of the enantiopure alcohols (*S*)-1-(4-fluorophenyl)ethan-1-ol-*d* ((*S*)-**1a-O$_D$**), (*S*)-1-(4-fluorophenyl)ethan-1-ol ((*S*)-**1a**), $(n\text{Bu})_3\text{SiH}$, styrene, and 4-fluoroanisole in C_6D_6 was assembled in a J-Young NMR tube according to general procedure GP1. The reaction was monitored at 298 K. Initial conditions and the temporal concentration profile are shown in Figure S44.

Concentrations of (*S*)-**1a** and (*S*)-**1a-O$_D$** were determined via the degree of deuteration of PhEt, which in turn was determined via the consumption of styrene and via the PhEt $-CH_3$ ^{1}H signal.

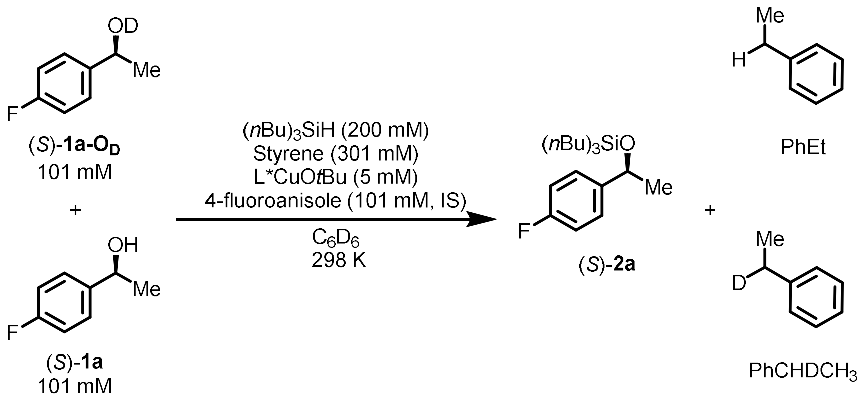

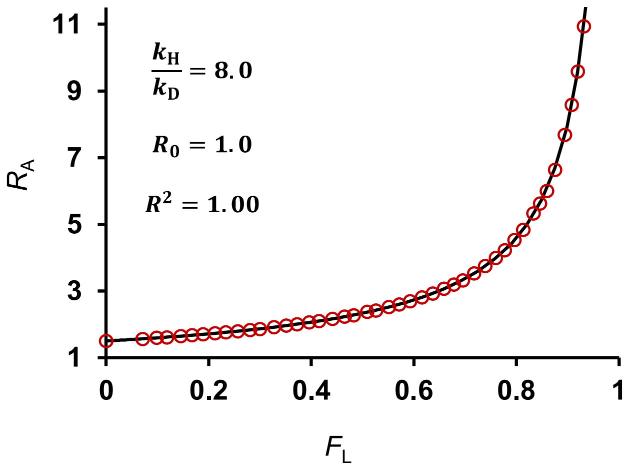

Figure S44. Initial reaction conditions and Bigeleisen-Wolfsberg[S5] plot of the competition reaction between (S)-**1a** and (S)-**1a-O$_D$**. The relative rate constant, initial substrate ratio R_0, and R^2 coefficient of determination from nonlinear fitting to Equation S2 are shown.

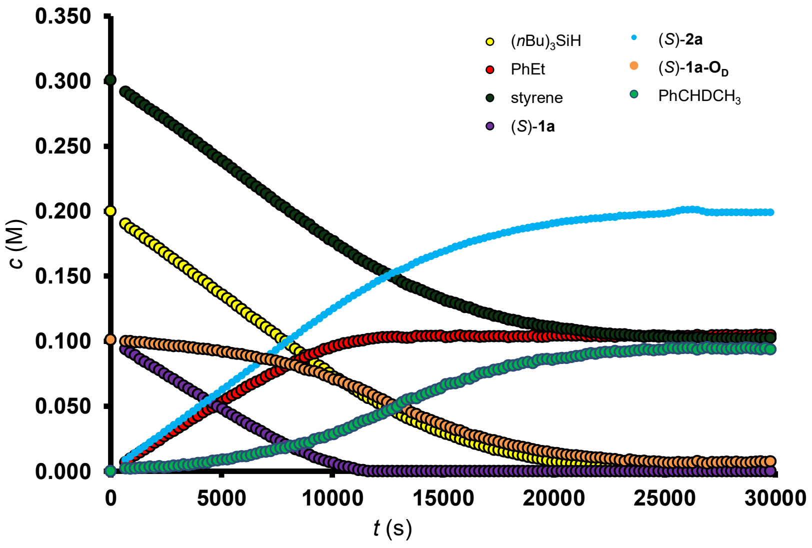

Figure S45. Temporal concentration profiles of reactants and products in the competition reaction between (S)-**1a** and (S)-**1a-O$_D$**. Concentrations of (S)-**1a** and (S)-**1a-O$_D$** were indirectly obtained from the respective concentrations of PhEt, $PhCHDCH_3$, and styrene.

4.8 Reaction of (*S*)-1a with (nBu)$_3$SiD

A reaction mixture of the enantiopure alcohol (*S*)-1-(4-fluorophenyl)ethan-1-ol ((*S*)-**1a**), (nBu)$_3$SiD, styrene, and 4-fluoroanisole in C_6D_6 was assembled in a J-Young NMR tube according to general procedure GP1. The reaction was monitored at 298 K. Initial conditions and the temporal concentration profile are shown in Figure S46.

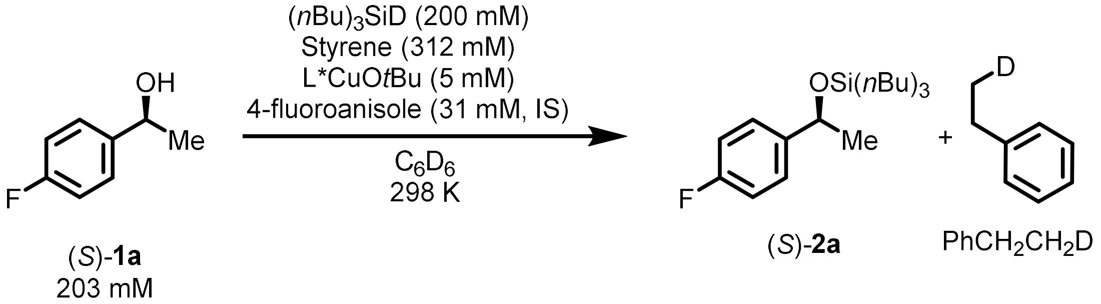

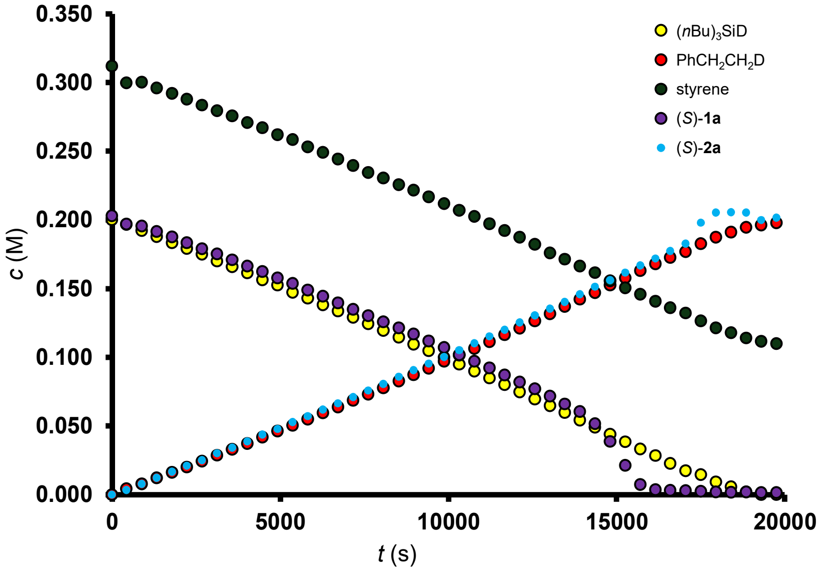

Figure S46. Initial reaction conditions and temporal concentration profiles of reactants and products in the reaction of (*S*)-1-(4-(methoxy)phenyl)ethan-1-ol ((*S*)-**1a**) and (nBu)$_3$SiD. Concentrations of (*S*)-**1a** (from *t* ~ 15000 s) shows artifacts resulting from peak drift of (*S*)-**1a** and subsequent overlap with (*S*)-**2a** at higher conversions.

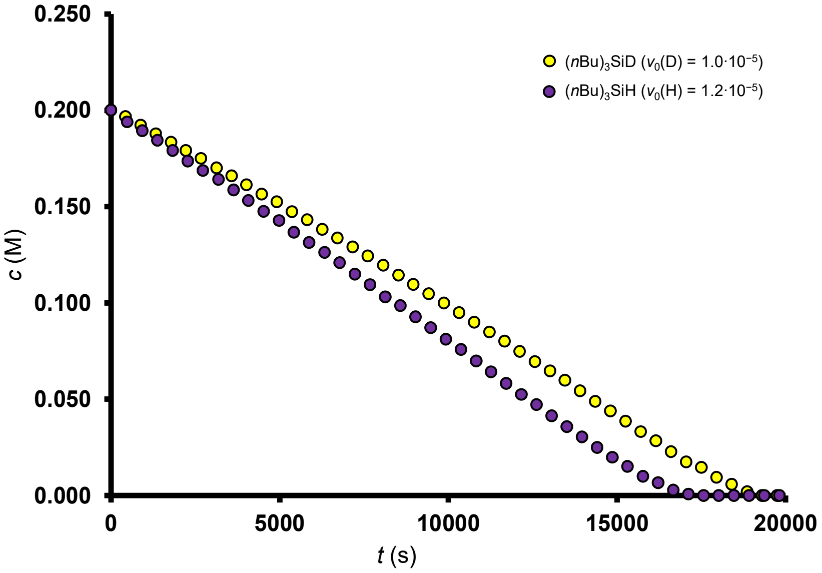

Figure S47. Comparison of temporal concentration profiles of (nBu)$_3$SiH and (nBu)$_3$SiD in their respective individual reactions under otherwise identical conditions. Initial rates v_0 are estimated from approximately 25% conversion, the initial rate ratio and therefore the SiH/SiD KIE via independent measurement is equal to 1.2.

Spectrum	Isotope Source	Product	
**I**	Reagents at Natural Abundance	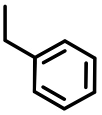	PhEt
**II**	$(n\text{Bu})_3\text{Si}\mathbf{D}$	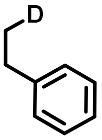	$PhCH_2CH_2D$
**III**	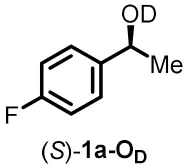 (*S*)-**1a-O$_D$**	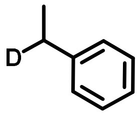	$PhCHDCH_3$

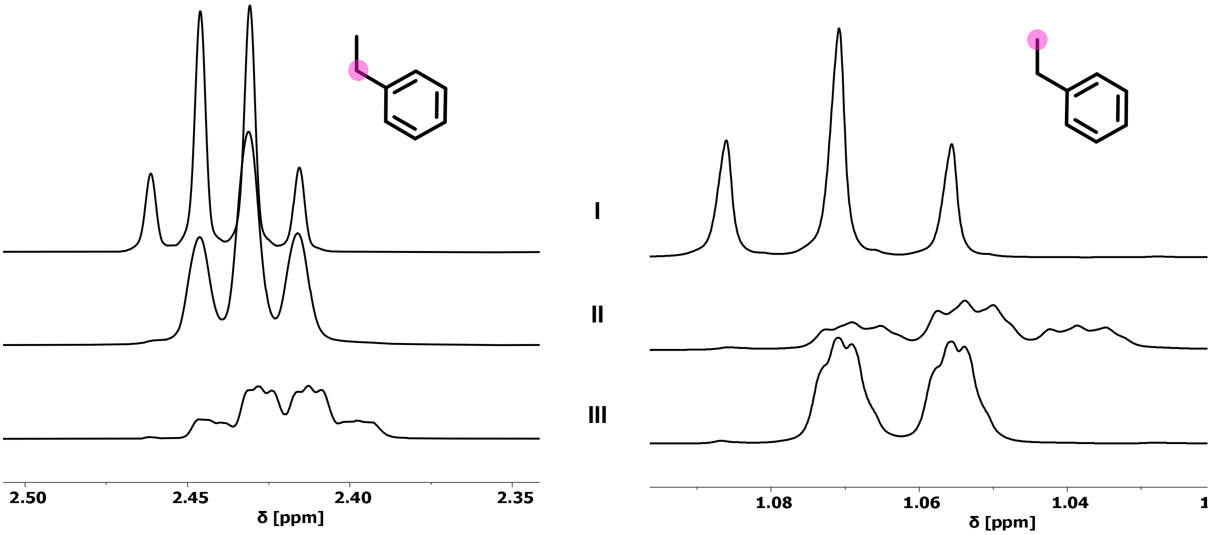

Figure S48. Comparison of final product spectra of PhEt in reactions employing reagents at natural isotopic abundance (Spectrum I, $(n\text{Bu})_3$SiH and (*S*)-**1a**, Section 3.1), deuteriated tri-*n*-butylsilane (Spectrum II, $(n\text{Bu})_3$SiD and (*S*)-**1a**, Figure S47), and deuteriated (*S*)-**1a** (Spectrum III, $(n\text{Bu})_3$SiH and (*S*)-**1a-O$_D$**, Figure S43). Isotope shifts, multiplicities, and integral ratios are consistent with selective deuteriation at the respective positions shown in the spectra.

4.9 Intermolecular Competition Reaction of (nBu)$_3$SiH vs (nBu)$_3$SiD and Kinetic Isotope Effect

A reaction mixture of the enantiopure alcohol (*S*)-1-(4-fluorophenyl)ethan-1-ol ((*S*)-**1a**), (nBu)$_3$SiH, (nBu)$_3$SiD, styrene, and 4-fluoroanisole in C_6D_6 was assembled in a J-Young NMR tube according to general procedure GP1. The reaction was monitored at 298 K. Initial conditions and the temporal concentration profile are shown in Figure S49. Concentrations of (nBu)$_3$SiD were indirectly determined via the degree of consumption of styrene and formation of (*S*)-**2a**.

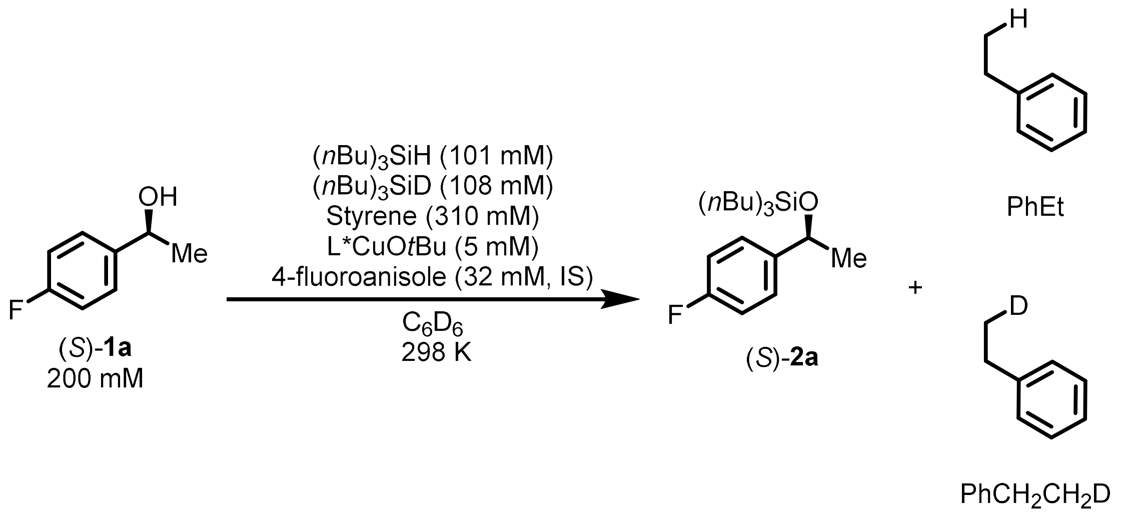

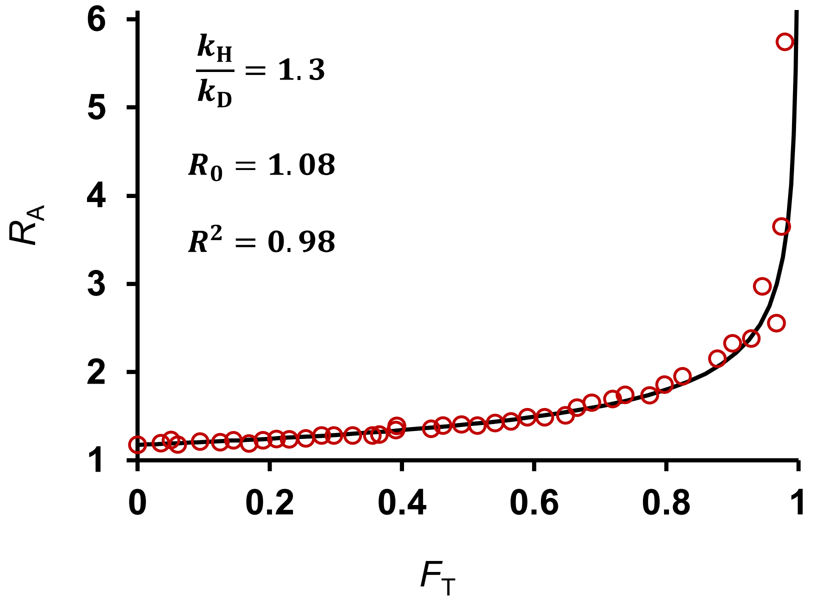

Figure S49. Initial reaction conditions and Bigeleisen-Wolfsberg[S5] plot of the competition reaction between (nBu)$_3$SiH and (nBu)$_3$SiD. The relative rate constant, initial substrate ratio R_0, and R^2 coefficient of determination from nonlinear fitting to Equation S2 are shown.

5. Catalyst System Investigations

5.1 Scalemic Mixtures of (*R*,*R*)-Ph-BPE and (*S*,*S*)-Ph-BPE

Inside a glovebox, the indicated amounts (Table S2) of (*R*,*R*)-Ph-BPE and (*S*,*S*)-Ph-BPE were dissolved in toluene (1 mL). The reaction vessel was sealed with a rubber septum, removed from the glovebox, and connected to a nitrogen-vacuum manifold. Toluene was evaporated under reduced pressure, and the colorless solid residue was further dried under oil pump vacuum overnight before being reintroduced into the glovebox. The enantiomeric excess was determined by HPLC analysis on a chiral stationary phase after oxidation with H_2O_2 following a literature procedure[S7] (Daicel Chiralpak® AD-H column, column temperature 20 °C, mobile phase: *n*-heptane:isopropanol = 90:10, flow rate: 0.8 mL/min, λ = 210 nm): t_R = 22.4 min for (*R*,*R*)-Ph-BPE, t_R = 26.7 min for (*S*,*S*)-Ph-BPE. Both ligands were determined to be phosphine-oxide free prior to oxidation.

Table S2. Weighed initial amounts of (*S*,*S*)-Ph-BPE and (*R*,*R*)-Ph-BPE and determined enantiomeric excess values for the final solid mixture of Ph-BPE.

Entry	(*R*,*R*)-Ph-BPE [mg]	(*S*,*S*)-Ph-BPE [mg]	ee [%]
1	15	15	0
2	18	12	19
3	21	9	42
4	24	6	58
5	27	3	77

The mixtures were employed in kinetic resolution reactions of *rac*-**1b** as follows (Table S3): Inside a glovebox, a 2 mL screw top vial was charged with CuCl (1.0 mg, 10 µmol, 5.0 mol%), NaO*t*Bu (1.0 mg, 10 µmol, 5.0 mol%), and (*R*,*R*)-Ph-BPE (indicated *ee*, 5.2 mg, 10 µmol, 5.0 mol%). Toluene (0.2 mL) was added, and the resulting colorless suspension was stirred for 10 min to give a pale yellow solution. Then, a stock solution containing *rac*-1-phenylethan-1-ol (*rac*-**1b**) $(n\text{Bu})_3$SiH, styrene, and tetracosane (0.25 M, 0.14 M, 0.38 M, and ~0.03 M, respectively, in toluene, 0.8 mL) was added using a 1.0 mL plastic syringe. The reaction mixture was stirred at the indicated temperature for 15 h, after which the conversion of alcohol **1b** is determined by GLC analysis. The resulting yellow solution was diluted with *tert*-butyl methyl ether, filtered over a pad of silica, and concentrated under reduced pressure. Flash column chromatography on silica gel (*n*-pentane:diethyl ether = gradient from 100:0 to 2:1) afforded silyl ether (*S*)-**2b** as a pale yellow oil and alcohol (*R*)-**1b** as a colorless oil. Silyl ether (*S*)-**2b** was deprotected according to GP5 prior to *ee* determination via HPLC analysis.

Table S3. Summary of results (negative) from experiments testing for nonlinear effects where the *ee* of the (*R*,*R*)-Ph-BPE ligand is varied.

Entry	$ee_{(R,R)\text{-Ph-BPE}}$ [%]	Conversion [%]	$ee_{(S)\text{-}\mathbf{2b}}$ [%]	$ee_{(R)\text{-}\mathbf{1b}}$ [%]
1	0	54	0	0
2	19	54	–13	15
3	42	54	–26	31
4	58	54	–37	44
5	77	54	–54	64
6	>99	54	–70	83

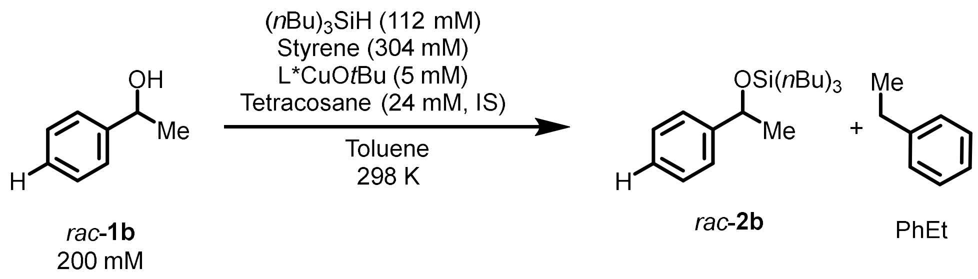

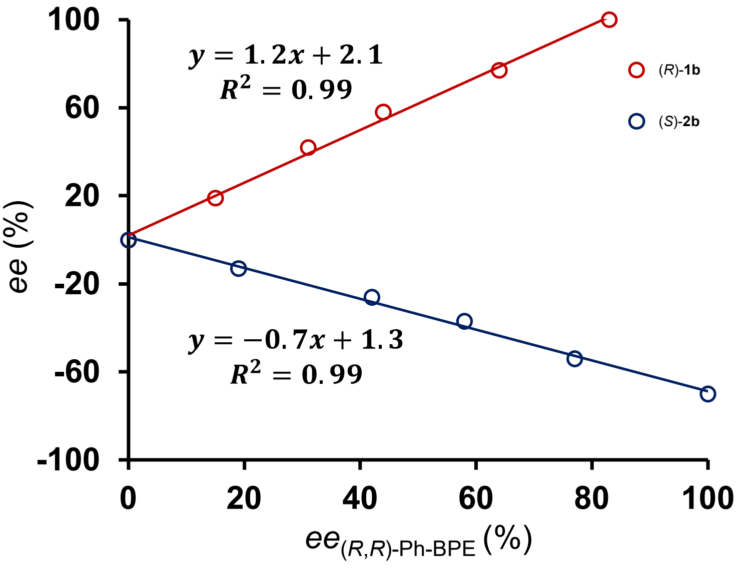

5.2 Reaction Initiation with CuO*t*Bu + (*R*,*R*)-Ph-BPE and with MesCu + (*R*,*R*)-Ph-BPE

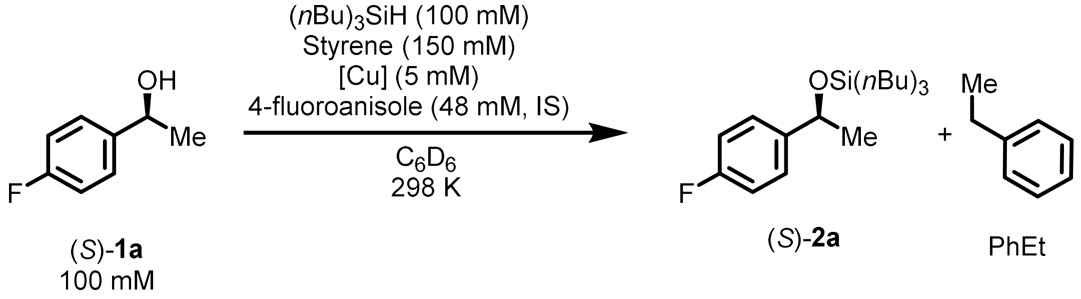

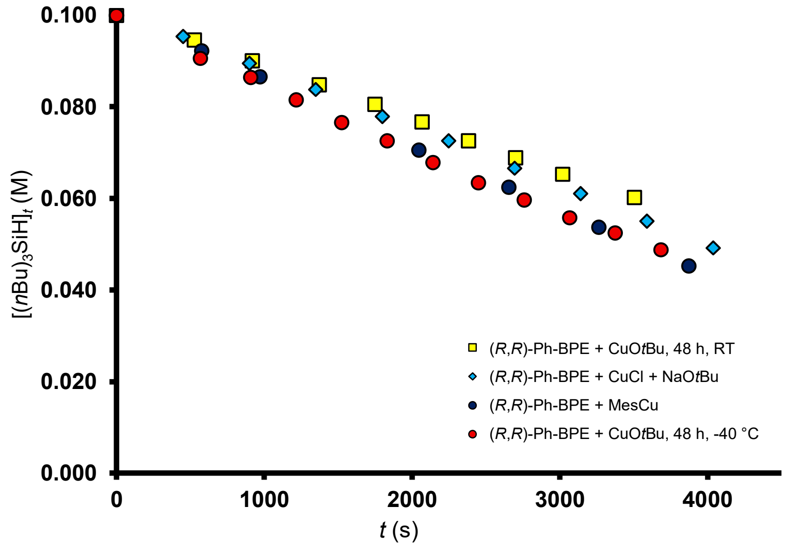

Figure S50. Comparison of temporal concentration profiles of (nBu)$_3$SiH in reactions where the Cu catalyst stock solution was prepared via GP6 (blue diamonds), by mixing stock solutions of (*R*,*R*)-Ph-BPE and of MesCu (dark blue circles), by mixing stock solutions of (*R*,*R*)-Ph-BPE and of CuO*t*Bu and stirring at RT in a glovebox for 48 h prior to use (red circles), and by mixing stock solutions of (*R*,*R*)-Ph-BPE and of CuO*t*Bu and storing in a glovebox freezer at –40°C for 48 h prior to use (red circles).

5.3 NMR Spectroscopic Investigations

In reactions with enantiopure (*S*)-**1a** (see Section 3.1 for details), a ^{1}H resonance of the (*R*,*R*)-Ph-BPE ligand is observed upfield (δ_H = –0.68 ppm) and stays constant under saturation conditions [(*S*)-**1a**]$_t$ >> [Cu]$_0$ (Figure S51, I). As [(*S*)-**1a**]$_t$ approaches [Cu]$_0$, the resonance disappears and one further upfield is observed (δ_H = –0.76 ppm). Similarly, when (*R*)-**1a** is used instead of (*S*)-**1a** (see Figure S7, Entry 16 for details), a resonance is observed at δ_H = –0.51 ppm under turnover conditions (Figure S51, II) and an identical resonance after depletion of (*R*)-**1a** observed at δ_H = –0.76 ppm, indicating the resonance at –0.76 ppm is enantiomer independent and likely does not contain **1a**. Under KR conditions (Section 3.4),

the resonance observed under turnover conditions drifts and converges to the shift obtained when only (*R*)-**1a** is present (δ_H = –0.51 ppm), indicating full consumption of (*S*)-**1a** and confirming the enantiomer concentration dependence of the observed chemical shift (Figure S51, III). In contrast, under DKR conditions (see Section 3.5 for details), a single, static resonance is observed (δ_H = –0.63 ppm) which differs slightly from the resonance observed for (*S*)-**1a** in isolation (Figure S51, IV), potentially being that of L*CuOR^S·(*R*)-**1a** (see Section 3.5 for discussion). In contrast, in the reaction run without added styrene (see Section 3.3 for discussion) the same resonance at δ_H = –0.68 ppm as in reaction with added styrene is initially present (Figure S51, V), but decays until it is under the detection limit of the experiment despite saturation conditions being operative ([(*S*)-**1a**]$_t$ >> [Cu]$_0$), while no resonance at δ_H = –0.76 ppm is observed as in all other cases (Figure S51, I–IV), potentially indicating that the signal at δ_H = –0.76 ppm is a product of hydrocupration of styrene. This is further corroborated by the earlier emergence of the signal at δ_H = –0.76 ppm when (*S*)-**1a-O$_D$** is used instead (Figure S51, IV, see Section 4.6 for details), which leads to the accumulation of L*Cu-CH(Me)Ph due to the large primary KIE in the deprotonation step (k_H/k_D = 8.0, see Section 4.7 for details). Furthermore, while the emergence of signals beyond the measured spectral window of –2.0 to 11.0 ppm cannot be excluded, the depletion of the signal at the observed range is consistent with the catalyst deactivation hypothesis discussed in Section 3.3.

Investigation of ^{31}P{^{1}H} signals present under turnover conditions [(*S*)-**1a**]$_t$ >> [Cu]$_0$ and after consumption of (*S*)-**1a** further supports the proposed identity of the resonance at δ_H = –0.76 ppm, as the hydrocuprated L*Cu-CH(Me)Ph species was investigated in detail by Buchwald and coworkers.[S8] At higher [(*S*)-**1a**]$_t$/[Cu]$_0$ ratios ([(*S*)-**1a**]$_t$/[Cu]$_0$ ≈ 10–12), only L*Cu-(*R*)-CH(Me)Ph is present in small quantities, and the majority of the catalyst speciation is attributed to L*CuOR^S·(*S*)-**1a**, as predicted by the kinetic model (Figure 9 in the main manuscript). With decreasing [(*S*)-**1a**]$_t$/[Cu]$_0$ ratios ([(*S*)-**1a**]$_t$/[Cu]$_0$ ≈ 5–7), the signal of L*Cu-(*R*)-CH(Me)Ph grows while that of L*CuOR^S·(*S*)-**1a** shifts upfield (Figure S52). After reaction completion, a further ^{31}P{^{1}H} spectrum was acquired, revealing the virtual disappearance of L*CuOR^S·(*S*)-**1a** and appearance of L*Cu-(*S*)-CH(Me)Ph. As hydrocupration of styrene via TS-II (Figure 9 in main manuscript) is proposed to strongly favor the formation of L*Cu-(*R*)-CH(Me)Ph, it is possible that L*Cu-CH(Me)Ph enantiomers interconvert under the employed conditions.[S8] Additionally, the sole observation of the L*Cu-(*R*)-CH(Me)Ph under reaction conditions could suggest that it is only present as a transient intermediate when [(*S*)-**1a**]$_t$ >> [Cu]$_0$, which is consistent with other experimental observations.

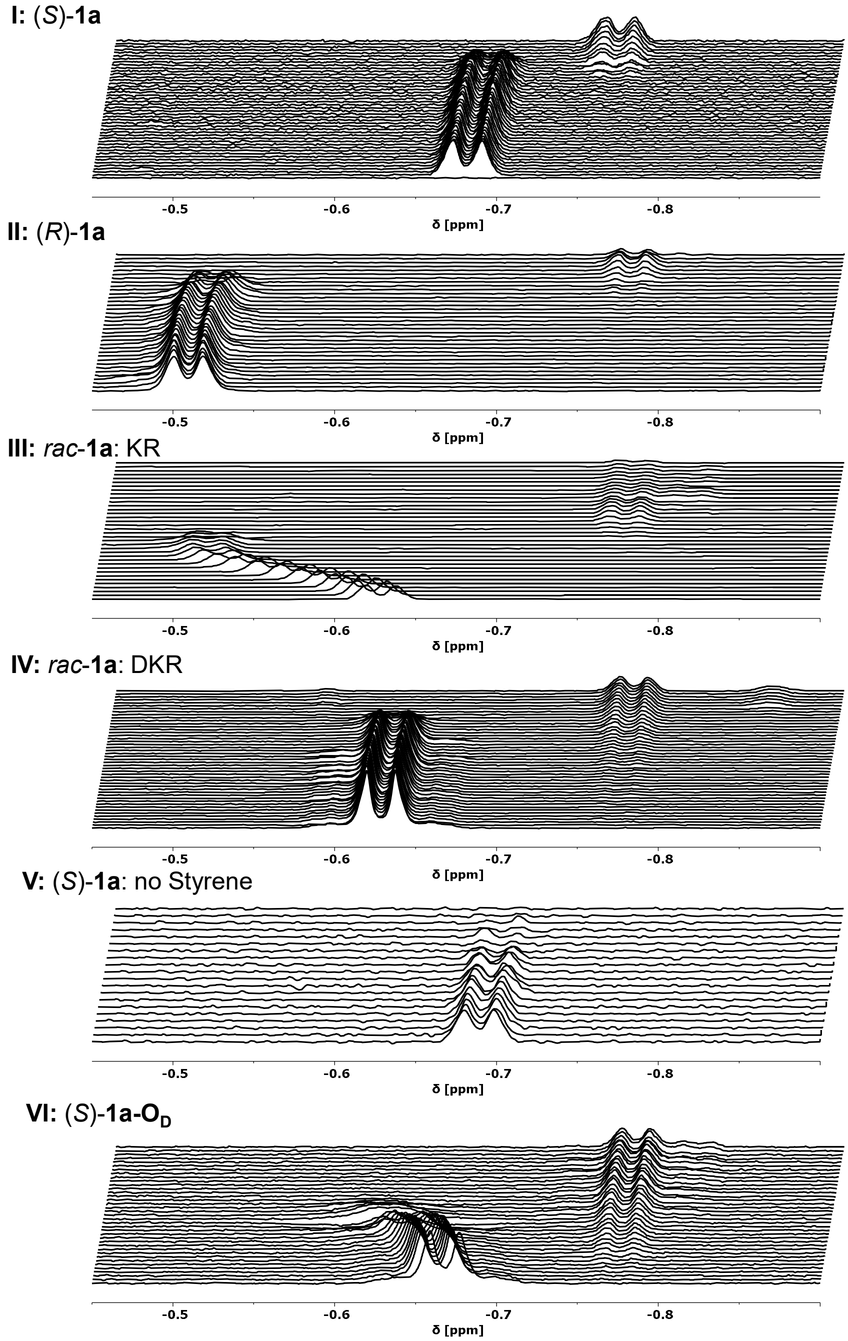

Figure S51. Stacked ^{1}H NMR spectra (δ_H = –0.45 ppm to –0.9 ppm) of various reaction mixtures (**I**: Figure S7, Entry 1, **II**: Figure S7, Entry 16, **III**: see Section 3.4, **IV**: see Section 3.5, **V**: see Section 3.3). Spectra for **I**–**IV** are shown over the course of the complete consumption of **1a** (Downfield signals present) and subsequent spectra after full consumption of **1a** (upfield signal present and disappearance of downfield signals). Spectra for **V** are truncated to t = 10000 s for clarity, as the shown signal decays but no further signals are present from δ_H = –0.0 ppm to –2.0 ppm (see Section 3.3 for discussion). Spectra for **VI** are shown over the course of the reaction of (*S*)-**1a**-**O$_D$** (see Section 4.6 for details), and the large primary KIE associated with the deprotonation of (*S*)-**1a**-**O$_D$** (k_H/k_D = 8.0, see Section 4.7 for details) likely results in the resonance at δ_H = –0.76 ppm being the dominant copper species during its reaction.

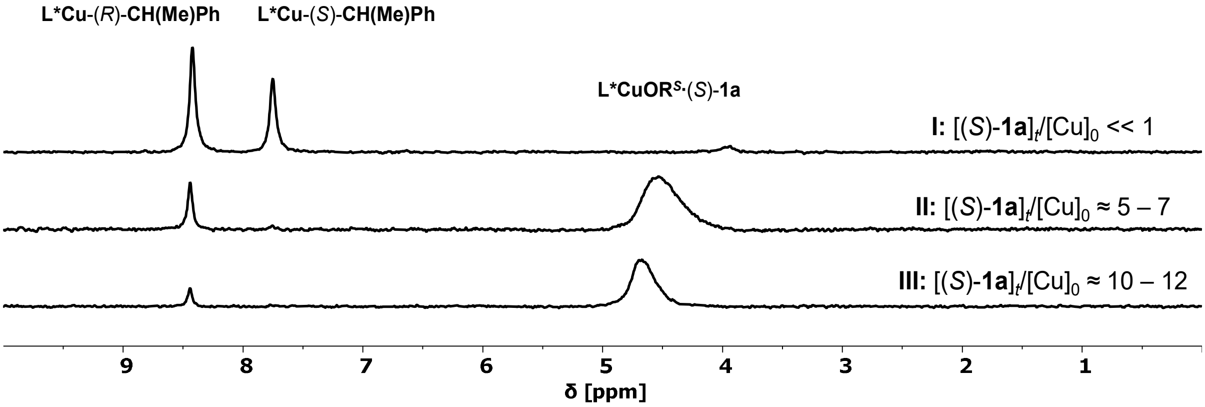

Figure S52. Stacked, time-averaged $^{31}P\{^1H\}$ NMR spectra (δ_P = 0 ppm to 10 ppm) of a reaction of (*S*)-**1a** under standard conditions (see Section 3.1 for details) at various $[(S)\text{-}\mathbf{1a}]_t/[Cu]_0$ ratios, which are given approximately due to the time-averaged nature of the spectra (acquired with multiple scans over approximately 15 minutes).

Attempts to form L^*CuOR^S in situ from L*CuO*t*Bu or L*MesCu and stoichiometric (*S*)-**1a** were generally unsuccessful, and attempted characterization via in situ NMR spectroscopy resulted in dynamic mixtures which were untractable to analysis. Addition of over 4 equivalents of (*S*)-**1a** (28 mM) to L*MesCu (7 mM) resulted in a system which was still highly dynamic, but which provided confirmation that in the absence of both styrene and $(nBu)_3SiH$, the ^1H and $^{31}P\{^1H\}$ signals observed in Figures S51 and S52 were still present and therefore likely result from interactions between (*S*)-**1a** and L^*CuOR^S (Figure S53). The apparent low stability of L^*CuOR^S in isolation is consistent with the documented tendency of Cu(I)-alkoxides of primary and secondary alcohols to spontaneously decompose.[S32] It is possible that the inhibitory equilibrium between L^*CuOR^S, (*S*)-**1a**, and L^*CuOR^S·(*S*)-**1a** is responsible for enabling the catalytic transformation, as the relative excess of (*S*)-**1a** present during reaction conditions results in low effective concentrations of L^*CuOR^S, and the relative excess of $(nBu)_3SiH$ irreversibly reacts with L^*CuOR^S faster than it decomposes. This is in contrast to CuO*t*Bu and the precatalyst L*CuO*t*Bu used throughout this work, as while L*CuO*t*Bu benzene solutions also result in spectroscopically intractable mixtures prior to reaction initiation, the benzene solutions are of considerably higher stability and result in reproducible catalytic assays (see Section 5.2).

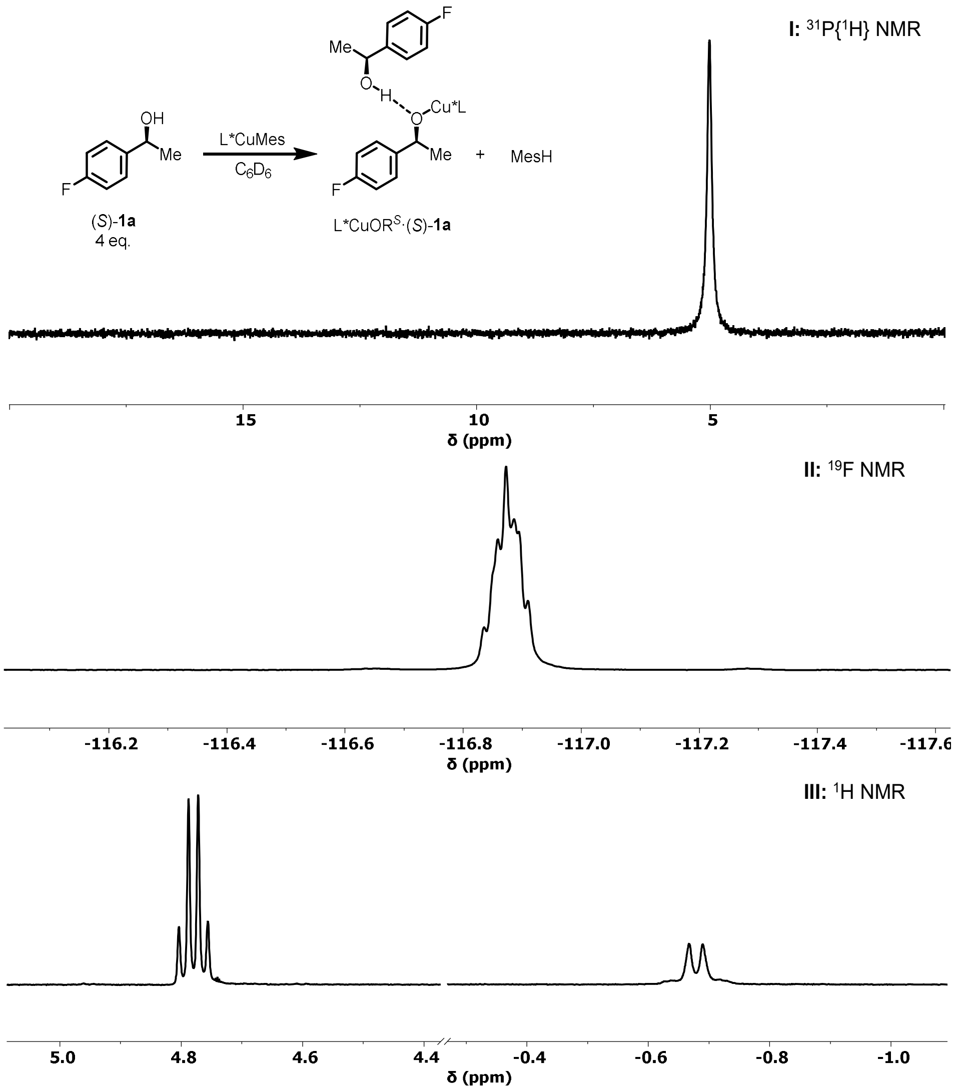

Figure S53. In situ addition of (*S*)-**1a** (4 equiv) to L*MesCu and the resulting $^{31}P\{^{1}H\}$ (I), ^{19}F (II), and ^{1}H (III) NMR spectra. The presence of the same resonances observed in Figures S51 and S52 was confirmed in the absence of both styrene and $(n\text{Bu})_3\text{SiH}$.

6. Synthetic Procedures and Characterization Data

The racemic alcohols *rac*-**1a**, *rac*-**1c**, *rac*-**1d**, and *rac*-**1e** were synthesized according to a known literature procedure[S1] (GP2) and their (*S*)- enantiomers were obtained via enzymatic acylation of the (*R*)- enantiomers[S2] (GP3). (*S*)-**1b** was obtained commercially. (*R*)-**1a** was obtained after ester hydrolysis (GP4) of the acetylated compound (*R*)-**3a**. *rac*-**1a-C$_D$** was synthesized via reduction of 4-fluoroacetophenone with $LiAlD_4$ according to a known literature procedure.[S9] CuO*t*Bu[S10] and MesCu[S11] were synthesized according to known literature procedures. $(n\text{Bu})_3SiD$ was prepared by reduction of $(n\text{Bu})_3SiCl$ with $LiAlD_4$ according to a literature procedure.[S12] Ru(CNN)(dppb)Cl was synthesized according to a literature procedure.[S4]

6.1 (*S*)-1a and (*R*)-3a

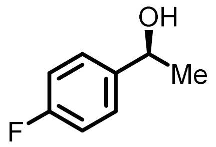

(*S*)-**1a**
C_8H_9FO
M = 140.16 g/mol

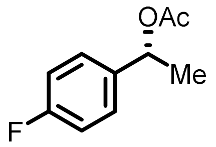

(*R*)-**3a**
$C_{10}H_{11}FO_2$
M = 182.19 g/mol

Prepared according to GP3 from rac-1-(4-fluorophenyl)ethan-1-ol (*rac*-**1a**, 13.5 g, 96.2 mmol, 1.00 equiv), vinyl acetate (4.54 g, 52.8 mmol, 0.549 equiv), and Novozym® 435 (0.58 g) in toluene (100 mL). The reaction mixture was stirred at room temperature overnight, after which full consumption of vinyl acetate and (*R*)-**1a** was verified by ^{1}H NMR analysis and HPLC analysis on a chiral stationary phase, respectively. Flash column chromatography on silica gel (MTBE:cyclohexane = gradient from 50:1 to 1:1) afforded (*R*)-1-(4-fluorophenyl)ethyl acetate, (*R*)-**3a**, 8.69 g, 47.7 mmol, 50%] and (*S*)-1-(4-fluorophenyl)ethan-1-ol [(*S*)-**1a**, 6.32 g, 45.1 mmol, 47%, >99% *ee*] as colorless oils. (*S*)-**1a** was further purified via Kugelrohr distillation prior to kinetic measurements.

Analytical data for (*S*)-**1a**:

R_f = 0.20 (cyclohexane:*tert*-butyl methyl ether = 4:1). **^{1}H NMR** (500 MHz, C_6D_6): δ/ppm = 1.18 (d, 6.4 Hz, 3H), 1.43 (br s, 1H), 4.39 (br q, 6.4 Hz, 1H), 6.80 (m, 2H), 6.97 (m, 2H). **^{13}C{^{1}H} NMR** (126 MHz, C_6D_6): δ/ppm = 25.5, 69.4, 115.2, 127.3, 142.4, 162.4. **^{19}F NMR** (471 MHz, C_6D_6): δ/ppm = –115.8 (m). **HRMS** (APCI) calculated for $C_8H_8FO^+$ [$(M–H)^+$]: 139.0554; found: 139.0552. The enantiomeric excess of (*S*)-**1a** was determined by HPLC analysis on a chiral stationary phase (Daicel Chiralcel® OJ-H column, column temperature: 20 °C, mobile phase: *n*-heptane:isopropanol = 97:3, flow rate: 0.3 mL/min, λ = 210 nm): t_R = 54.0 min for (*S*)-**1a**. Analytical data are in good agreement with that reported in literature.[S30]

Analytical data for (*R*)-**3a**:

R$_f$ = 0.17 (cyclohexane:*tert*-butyl methyl ether = 50:1). **^{1}H NMR** (500 MHz, C_6D_6): δ/ppm = 1.25 (d, 6.6 Hz, 3H), 1.64 (s, 3H), 5.81 (q, 6.6 Hz, 1H), 6.77 (m, 2H), 7.01 (m, 2H). **^{13}C{^{1}H} NMR** (126 MHz, C_6D_6): δ/ppm = 20.7, 22.1, 71.4, 115.5, 128.3, 138.1, 162.7, 169.3. **^{19}F NMR** (471 MHz, C_6D_6): δ/ppm = –114.5 (m). Analytical data are in good agreement with that reported in literature.[S2]

6.2 (*R*)-1a

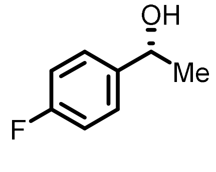

(*R*)-**1a**
C_8H_9FO
M = 140.16 g/mol

Prepared according to GP4 from (*R*)-**3a** (47.7 mmol, 1.0 equiv), using a solution of NaOH (2.2 M in water, 95 mL, 0.21 mol, 4.4 equiv) in methanol (95 mL). The reaction mixture was stirred at room temperature for 1 h, after which full consumption of (*R*)-**3a** was verified by TLC analysis. The aqueous phase was extracted with MTBE (2 × 200 mL). The combined organic layers were washed with water (3 × 100 mL) and brine (100 mL), dried over $MgSO_4$, filtered, and concentrated to afford (*R*)-1-phenylethan-1-ol [(*R*)-**1a**, 6.46 g, 46.0 mmol, 96%] as a colorless oil. To increase enantiopurity, the total amount was resubjected to enzymatic acylation (GP3) with vinyl acetate (1.58 g, 18.4 mmol, 0.399 equiv) and Novozym® 435 (0.28 g, 6.1 g/mol) in toluene (50 mL). The reaction mixture was stirred at room temperature overnight, after which full consumption of vinyl acetate was verified by ^{1}H NMR analysis. Flash column chromatography on silica gel (MTBE:cyclohexane = gradient from 40:1 to 15:1) afforded (*R*)-**3a** as a colorless oil (3.30 g, 18.1 mmol, 39%), which was subsequently hydrolyzed according to GP4, separated via column chromatography, and further purified via vacuum distillation (3.8 mbar, 130 °C) affording (*R*)-**1a** as a colorless oil (2.23 g, 15.9 mmol, 33%, 99% *ee*)

Analytical data for (*R*)-**1a**:

Spectroscopically identical to (*S*)-**1a**. The enantiomeric excess of (*R*)-**1a** was determined by HPLC analysis on a chiral stationary phase (Daicel Chiralcel® OJ-H column, column temperature: 20 °C, mobile phase: *n*-heptane:isopropanol = 97:3, flow rate: 0.3 mL/min, λ = 210 nm): t_R = 56.9 min for (*R*)-**1a**.

6.3 (*S*)-1c

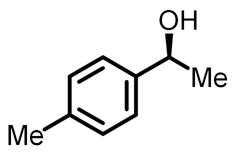

(S)-**1c**
$C_9H_{12}O$
M = 136.19 g/mol

Prepared according to GP3 from *rac*-**1c**, (6.64 g, 48.8 mmol, 1.00 equiv), vinyl acetate (2.51 g, 29.2 mmol, 0.598 equiv), and Novozym® 435 (0.29 g, 5.9 g/mol) in toluene (75 mL). The reaction mixture was stirred at room temperature overnight, after which full consumption of vinyl acetate was verified by ^{1}H NMR analysis. Flash column chromatography on silica gel (MTBE:cyclohexane = gradient from 50:1 to 4:1) afforded (S)-**1c**, 3.02 g, 22.2 mmol, 45%, >99% *ee*] as a colorless oil. (S)-**1c** was further purified via Kugelrohr distillation prior to kinetic measurements.

Analytical data for (S)-**1c**:

R_f = 0.27 (cyclohexane:*tert*-butyl methyl ether = 4:1). **^{1}H NMR** (400 MHz, C_6D_6): δ/ppm = 1.33 (d, 6.4 Hz, 3H), 2.05 (br s, 1H), 2.13 (s, 3H), 4.59 (q, 6.4 Hz, 1H), 7.00 (m, 2H), 7.17 (m, 2H). **^{13}C{^{1}H} NMR** (101 MHz, C_6D_6): δ/ppm = 21.1, 25.6, 70.1, 125.7, 129.2, 136.7, 144.0. **HRMS** (APCI) calculated for $C_9H_{11}O^+$ [$(M–H)^+$]: 135.0804; found: 135.0803. The enantiomeric excess of (S)-**1c** was determined by HPLC analysis on a chiral stationary phase (Daicel Chiralcel® OJ-H column, column temperature: 20 °C, mobile phase: *n*-heptane:isopropanol = 98:2, flow rate: 0.8 mL/min, λ = 210 nm): t_R = 25.6 min for (S)-**1c**. Analytical data are in good agreement with that reported in literature.[S30]

6.4 (*S*)-1d

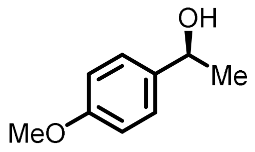

(S)-**1d**
$C_9H_{12}O_2$
M = 152.19 g/mol

Prepared according to GP3 from *rac*-**1d**, (6.03 g, 39.6 mmol, 1.00 equiv), vinyl acetate (2.05 g, 23.8 mmol, 0.598 equiv), and Novozym® 435 (0.24 g, 6.1 g/mol) in toluene (60 mL). The reaction mixture was stirred at room temperature overnight, after which full consumption of vinyl acetate was verified by ^{1}H NMR analysis. Flash column chromatography on silica gel (MTBE:cyclohexane = gradient from 50:1 to 2:1) afforded (S)-**1d**, 2.55 g, 16.8 mmol, 42%,

>99% *ee*] as a colorless oil. (*S*)-**1d** was further purified via Kugelrohr distillation prior to kinetic measurements.

Analytical data for (*S*)-**1d**:

R$_f$ = 0.16 (cyclohexane:*tert*-butyl methyl ether = 4:1). **^{1}H NMR** (400 MHz, C_6D_6): δ/ppm = 1.34 (d, 6.4 Hz, 3H), 1.83 (br s, 1H), 3.32 (s, 3H), 4.59 (q, 6.4 Hz, 1H), 6.79 (m, 2H), 7.17 (m, 2H). **^{13}C{^{1}H} NMR** (101 MHz, C_6D_6): δ/ppm = 25.6, 54.8, 69.8, 114.0, 126.9, 139.0, 159.4. **HRMS** (APCI) calculated for $C_9H_{11}O_2^+$ [(M–H)$^+$]: 151.0754; found: 151.0753. The enantiomeric excess of (*S*)-**1d** was determined by HPLC analysis on a chiral stationary phase (Daicel Chiralcel® OJ-H column, column temperature: 20 °C, mobile phase: *n*-heptane:isopropanol = 98:2, flow rate: 0.8 mL/min, λ = 230 nm): t_R = 61.8 min for (*S*)-**1d**. Analytical data are in good agreement with that reported in literature.[S30]

6.5 (*S*)-1e

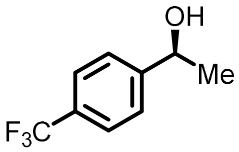

(*S*)-**1e**
$C_9H_9F_3O$
M = 190.17 g/mol

Prepared according to GP3 from *rac*-**1e**, (7.21 g, 37.9 mmol, 1.00 equiv), vinyl acetate (1.96 g, 22.8 mmol, 0.6 equiv), and Novozym® 435 (0.23 g, 6.1 g/mol) in toluene (60 mL). The reaction mixture was stirred at room temperature overnight, after which full consumption of vinyl acetate was verified by ^{1}H NMR analysis. Flash column chromatography on silica gel (MTBE:cyclohexane = gradient from 20:1 to 2:1) afforded (*S*)-**1e**, 3.12 g, 16.4 mmol, 43%, >99% *ee*] as a colorless oil. (*S*)-**1e** was further purified via Kugelrohr distillation prior to kinetic measurements

Analytical data for (*S*)-**1e**:

R$_f$ = 0.19 (cyclohexane:*tert*-butyl methyl ether = 4:1). **^{1}H NMR** (500 MHz, C_6D_6): δ/ppm = 1.12 (d, 6.5 Hz, 3H), 1.49 (br s, 1H), 4.34 (q, 6.5 Hz, 1H), 7.01 (m, 2H), 7.35 (m, 2H). **^{13}C{^{1}H} NMR** (126 MHz, C_6D_6): δ/ppm = 25.4, 69.4, 125.1, 125.5, 125.9, 129.5, 150.5. **^{19}F NMR** (471 MHz, C_6D_6): δ/ppm = –62.1 (m). **HRMS** (APCI) calculated for $C_9H_8F_3O_2^+$ [(M–H)$^+$]: 189.0522; found: 189.0521. The enantiomeric excess of (*S*)-**1e** was determined by HPLC analysis on a chiral stationary phase (Daicel Chiralcel® AD-H column, column temperature: 20 °C, mobile phase: *n*-heptane:isopropanol = 99.7:0.3, flow rate: 0.8 mL/min, λ = 210 nm): t_R = 71.6 min for (*S*)-**1e**. Analytical data are in good agreement with that reported in literature.[S31]

6.6 (*S*)-1a-C_D + (*R*)-3a-C_D

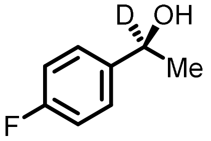

(*S*)-**1a-C_D**
C_8H_8DFO
M = 141.16 g/mol

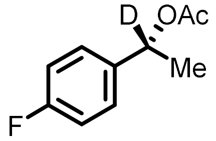

(*R*)-**3a-C_D**
$C_{10}H_{10}DFO_2$
M = 183.20 g/mol

Prepared according to GP3 from *rac*-**1a-C_D**, (1.18 g, 8.36 mmol, 1.00 equiv), vinyl acetate (0.18 g, 2.1 mmol, 0.25 equiv), and Novozym® 435 (0.05 g, 6.1 g/mol) in toluene (12 mL). The reaction mixture was stirred at room temperature overnight, after which full consumption of vinyl acetate was verified by ^{1}H NMR analysis. Flash column chromatography on silica gel (MTBE:cyclohexane = gradient from 50:1 to 3:1) afforded a mixture of (*R*)- and (*S*)-**1a-C_D**, (0.9 g, 6.4 mmol, 76%) and (*R*)-**1a-C_D**, (0.325 g, 1.8 mmol, 21%) as colorless oils. The obtained mixture of (*R*)- and (*S*)-**1a-C_D** was resubjected to enzymatic kinetic resolution conditions using vinyl acetate (0.22 g, 2.6 mmol, 0.4 equiv), and Novozym® 435 (0.05 g, 7.8 g/mol) in toluene (12 mL) and stirred at room temperature overnight, after which full consumption of vinyl acetate was verified by ^{1}H NMR analysis. Flash column chromatography on silica gel (MTBE:cyclohexane = gradient from 20:1 to 2:1) afforded (*S*)-**1a-C_D**, (0.48 g, 3.4 mmol, 40%, >99% *ee*) as a colorless oil. (*S*)-**1a-C_D** was further purified via Kugelrohr distillation prior to kinetic measurements.

Analytical data for (*S*)-**1a-C_D**:

^{1}H NMR (400 MHz, C_6D_6): δ/ppm = 1.17 (br s, 3H), 1.48 (br s, 1H), 6.80 (m, 2H), 6.97 (m, 2H). **^{2}H NMR** (77 MHz, C_6D_6): δ/ppm = 4.34 (s). **^{13}C{^{1}H} NMR** (101 MHz, C_6D_6): δ/ppm = 25.4, 69.0, 115.2, 127.3, 142.4, 162.4. **^{19}F NMR** (471 MHz, C_6D_6): δ/ppm = –115.8 (br m). **IR** (ATR): $\tilde{\nu}$/cm^{-1} = 3327 (m, br), 2972 (w), 1602 (m), 1508 (s), 1368 (m), 1220 (s), 1132 (s), 1088 (s), 931 (m), 815 (s). **HRMS** (APCI) calculated for $C_8H_7DF^+$ [(M–OH)$^+$]: 124.0667; found: 124.0665. Analytical data are otherwise identical to those of the isotopologue (*S*)-**1a** and is in good agreement with that reported in literature[S9]

Analytical data for (*R*)-**3a-C_D**:

^{1}H NMR (400 MHz, C_6D_6): δ/ppm = 1.51 (br s, 3H), 2.06 (s, 3H), 7.03 (m, 2H), 7.33 (m, 2H). **^{13}C{^{1}H} NMR** (101 MHz, C_6D_6): δ/ppm = 21.4, 22.2, 71.4, 115.5, 128.1, 137.6, 162.5, 170.4. Analytical data are in good agreement to those of the isotopologue (*R*)-**3a**.

6.7 (*R*)-1a-C_D

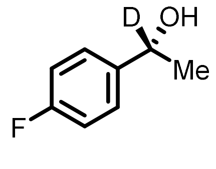

(*R*)-**1a-C_D**
C_8H_8DFO
M = 141.16 g/mol

Prepared according to GP4 from (*R*)-**3a-C_D** (300 mg, 1.64 mmol, 1.0 equiv), using a solution of NaOH (2.2 M in water, 3.3 mL, 7.3 mmol, 4.4 equiv) in methanol (3 mL). The reaction mixture was stirred at room temperature for 1 h, after which full consumption of (*R*)-**3a-C_D** was verified by TLC analysis. Water (10 mL) was added, and the aqueous phase was extracted with MTBE (4 × 25 mL). The combined organic layers were washed with water (10 mL) and brine (10 mL), dried over $MgSO_4$, filtered, and concentrated to afford crude (*R*)-1-phenylethan-1-ol ((*R*)-**1a**), which was purified via vacuum distillation (1.0 mbar, 90 °C) affording (*R*)-**1a-C_D** as a colorless oil (0.22 g, 1.54 mmol, 94%, >99% *ee*)
Analytical data for (*R*)-**1a-C_D**:
Spectroscopically identical to (*S*)-**1a-C_D**. The enantiomeric excess of (*R*)-**1a** was determined by HPLC analysis on a chiral stationary phase (Daicel Chiralcel® OJ-H column, column temperature: 20 °C, mobile phase: *n*-heptane:isopropanol = 97:3, flow rate: 0.3 mL/min, λ = 210 nm): t_R = 56.7 min for (*R*)-**1a-C_D**.

6.8 (*S*)-1a-O_D

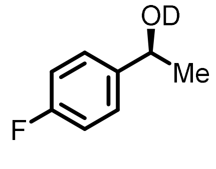

(*S*)-**1a-O_D**
C_8H_8DFO
M = 141.16 g/mol

An oven-dried two-necked flask equipped with a short-path distillation apparatus was charged with (*S*)-1-(4-fluorophenyl)ethan-1-ol ((*S*)-**1a**, 2.80 g, 20.0 mmol, 1.00 equiv), D_2O (1.5 mL, 1.7 g, 83 mmol, 4.2 equiv) was added, and the biphasic mixture was stirred vigorously at 60 °C for 1.5 h. After cooling to room temperature, all volatiles were removed under oil pump vacuum. The process was repeated three times with D_2O (1.5 mL, 1.7 g, 83 mmol, 4.2 equiv) and three times with methanol-d_4 (2.0 mL, 1.8 g, 49 mmol, 2.5 equiv). Purification of the residue by fractional vacuum distillation afforded (*S*)-1-(4-fluorophenyl)ethan-1-ol-*d*

((*S*)-**1a**-**O$_D$**, 2.35 g, 16.7 mmol, 83%, >99% *ee*). Quantitative ^{1}H NMR analysis of the product indicated >98% D incorporation at the hydroxy group.

Analytical data for (*S*)-**1a-O$_D$**:

^{1}H NMR (500 MHz, C_6D_6): δ/ppm = 1.18 (d, 6.5 Hz, 3H), 4.39 (q, 6.5 Hz, 1H), 6.79 (m, 2H), 6.97 (m, 2H). **^{2}H NMR** (77 MHz, C_6D_6): δ/ppm = 1.36 (br s). Analytical data agrees with that of isotopologue (*S*)-**1a** and is in good agreement with that reported in literature.[S9]

7. Computational Investigations

7.1 General Considerations

All DFT calculations were carried out at the High-Performance Computing Center (HPCC) of Nanjing University unless otherwise stated. Theoretical KIE calculations and conformational searches were calculated at the University of Edinburgh Compute and Data Facility (ECDF). All calculations have been performed using Gaussian 16 revision C.01,[S13] ORCA 5.0.2,[S14] and xtb version 6.7.0.[S15] Unless otherwise stated, parameters for the respective calculations are the respective program's default parameters. Geometry optimization and frequency calculations were run in Gaussian 16 without implicit solvation employing the B3LYP[S16] density functional including the D3(BJ)[S17] empirical dispersion correction as implemented in Gaussian 16, and the 6-31G* basis set[S18] for all atoms except Cu, and the SDD basis set[S19] for Cu. Frequency calculations were performed to characterize stationary points as either minima (no imaginary frequencies) or transition states (one imaginary frequency). For both minima and transition states, if any additional imaginary frequencies of ≥ −50 cm^{-1} were present they were disregarded. The final geometries of transition states were verified via intrinsic reaction coordinate (IRC)[S20] calculations. All stationary point geometries are provided in a separate .xyz file. Standard state thermochemical corrections (T = 298.15 K, p = 1 atm, c = 1 M, frequency cutoff of 50 cm^{-1}) were obtained with the GoodVibes[S21] program (keywords: --qs grimme --qh -t 298 -c 1 -f 50 -v 1.0). Where relevant, conformational sampling of minima and transition state structures was carried out using crest 3.0.2[S22] with the GFN2-xTB method. Only the lowest energy conformers were reported.

Single point calculations were run in ORCA 5.0.2 with the ωB97X-V[S23] density functional, the def2-TZVPP[S24] basis set for all atoms except Cu, the def2-QZVPP[S24] basis set for Cu, and the def2/J[S25] auxiliary basis for all atoms. Implicit solvation (benzene) was modeled employing the SMD[S26] continuum solvation model. The RIJCOSX[S27] and RI-J[S28] approximations were applied to all single point calculations. Theoretical kinetic isotope effects (KIEs) were computed using the Bigeleisen-Mayer equation (T = 298.15 K) in conjunction with the Wigner quantum tunnelling correction, as implemented in the PyQuiver program.[S29] The chosen computational method used for calculations within this work is abbreviated as: ωB97X-V-SMD(C_6H_6)/def2-QZVPP(Cu),def2-TZVPP(C,H,F,O,Si,P)//B3LYP-D3(BJ)/6-31G*(C,H,F,O,Si,P),SDD(Cu).

7.2 Aggregation of 1a

Direct, off-cycle aggregation of **1a** was found to be unfavorable within the computational model, and formation of an (*S*)-**1a***(*S*)-**1a** dimer (ΔG = 3.1 kcal·mol^{-1}), an (*S*)-**1a***(*R*)-**1a** dimer (ΔG = 3.4 kcal·mol^{-1}), or an (*R*)-**1a***(*R*)-**1a** dimer (ΔG = 3.0 kcal·mol^{-1}) was in all cases endergonic (See Section 8.1 for discussions about off-cycle aggregation of **1a** within the model). Investigations of catalyst speciation in the presence of **1a** predict H-bonding to be in all cases more favorable than direct coordination of **1a** oxygen to L*CuOR by at least 3.2 kcal·mol^{-1} in cases where a single **1a** molecule acts as an inhibitor (Figure S54, A), which is consistent with experimental evidence concerning the dominant mode of inhibition by alcohols in the reaction (see Figure 7 in main manuscript). Additionally, other possible higher modes of aggregation of **1a** and L*CuOR were investigated, and it was found that when forming a higher aggregate with two (*S*)-**1a** molecules, L*CuOR^{S} is predicted to favor H-bonding in a linear (*S*)-**1a** +(*S*)-**1a** + L*CuOR^{S} fashion arrangement, as in L*CuOR^{S}·(*S*)-**1a**·(*S*)-**1a** (see Figure 9 in main manuscript). A cyclic H-bond network with two oxygens coordinated to copper (denoted as CuCoord, 2.0 kcal·mol^{-1} higher in energy than linear L*CuOR^{S}·(*S*)-**1a**·(*S*)-**1a**) or two (*S*)-**1a** molecules directly H-bonded to L*CuOR^{S} (denoted as OCoord, 1.3 kcal·mol^{-1} higher in energy than linear L*CuOR^{S}·(*S*)-**1a**·(*S*)-**1a**) are both shown to be disfavored (Figure S54, B). For a system only containing (*R*)-**1a**, the most favorable isomer of L*CuOR^{R}·(*R*)-**1a**·(*R*)-**1a** is one where two (*R*)-**1a** molecules are directly H-bonded to the alkoxide oxygen in L*CuOR^{R}. In cases where mixed alcohol aggregates are present, linear adducts were found to be most favorable for two combinations (L*CuOR^{S}·(*S*)-**1a**·(*R*)-**1a**, L*CuOR^{S}·(*R*)-**1a**·(*S*)-**1a**), whereas cyclic H-bond networks with two oxygens coordinated to copper are favored for three combinations (L*CuOR^{S}·(*R*)-**1a**·(*R*)-**1a**, L*CuOR^{R}·(*S*)-**1a**·(*S*)-**1a**, L*CuOR^{R}·(*R*)-**1a**·(*S*)-**1a**), and an adduct where two **1a** molecules are directly H-bonded to the alkoxide oxygen is only the most favorable for the L*CuOR^{S}·(*R*)-**1a**·(*S*)-**1a** combination. We note that these were mainly calculated to demonstrate feasibility, and that the inclusion of such adducts in the model is a compromise to account for the dynamic nature of **1a** aggregates while maintaining a robust kinetic model, and their prevalence in a given system is proportional not only to their standard-state free energy differences, but also to the square of **1a** concentrations.

All free energies below are referenced to the following system

LCuOR^{S} + (*S*)-**1a** + (*S*)-**1a** + (*R*)-**1a** + (*R*)-**1a** + (*R*)-**1a**

A: Single-Alcohol Inhibition Adducts of L*CuOR, (*S*)-**1a, and** (*R*)-**1a**

Cu Coordination Adduct	H-Bonding Adduct
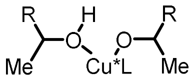	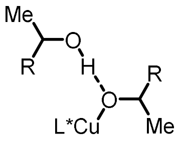
L*CuOR^{R}·(*R*)-**1a**: 2.1	L*CuOR^{R}·(*R*)-**1a**: −1.1
L*CuOR^{R}·(*S*)-**1a**: 1.8	L*CuOR^{R}·(*S*)-**1a**: −2.5
L*CuOR^{S}·(*R*)-**1a**: 2.1	L*CuOR^{S}·(*R*)-**1a**: −3.6
L*CuOR^{S}·(*S*)-**1a**: 7.1	L*CuOR^{S}·(*S*)-**1a**: −3.5

B: Higher-Order Inhibition Adducts of L*CuOR, (*S*)-**1a, and** (*R*)-**1a**

Cu Coordinated, Cyclic Adducts (CuCoord)	H-Bond Bridged Adducts, Same Acceptor (OCoord)	H-Bond Bridged Adducts (Linear)
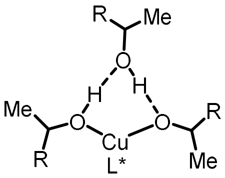	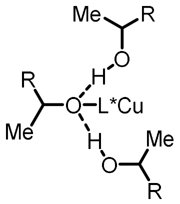	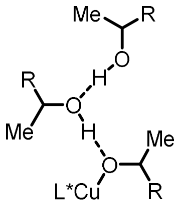
L*CuOR^{R}·(*R*)-**1a**·(*R*)-**1a**: 2.6	L*CuOR^{R}·(*R*)-**1a**·(*R*)-**1a**: 0.0	L*CuOR^{R}·(*R*)-**1a**·(*R*)-**1a**: 0.3
L*CuOR^{R}·(*R*)-**1a**·(*S*)-**1a**: −3.3	L*CuOR^{R}·(*R*)-**1a**·(*S*)-**1a**: 2.0	L*CuOR^{R}·(*R*)-**1a**·(*S*)-**1a**: 0.9
L*CuOR^{R}·(*S*)-**1a**·(*R*)-**1a**: −2.5	L*CuOR^{R}·(*S*)-**1a**·(*R*)-**1a**: −3.4	L*CuOR^{R}·(*S*)-**1a**·(*R*)-**1a**: −1.5
L*CuOR^{R}·(*S*)-**1a**·(*S*)-**1a**: −4.7	L*CuOR^{R}·(*S*)-**1a**·(*S*)-**1a**: −1.2	L*CuOR^{R}·(*S*)-**1a**·(*S*)-**1a**: −1.2
L*CuOR^{S}·(*R*)-**1a**·(*R*)-**1a**: −4.9	L*CuOR^{S}·(*R*)-**1a**·(*R*)-**1a**: −3.1	L*CuOR^{S}·(*R*)-**1a**·(*R*)-**1a**: 0.7
L*CuOR^{S}·(*R*)-**1a**·(*S*)-**1a**: −1.5	L*CuOR^{S}·(*R*)-**1a**·(*S*)-**1a**: −0.8	L*CuOR^{S}·(*R*)-**1a**·(*S*)-**1a**: −1.6
L*CuOR^{S}·(*S*)-**1a**·(*R*)-**1a**: 0.8	L*CuOR^{S}·(*S*)-**1a**·(*R*)-**1a**: −4.7	L*CuOR^{S}·(*S*)-**1a**·(*R*)-**1a**: −4.8
L*CuOR^{S}·(*S*)-**1a**·(*S*)-**1a**: −2.8	L*CuOR^{S}·(*S*)-**1a**·(*S*)-**1a**: −2.1	L*CuOR^{S}·(*S*)-**1a**·(*S*)-**1a**: −4.1

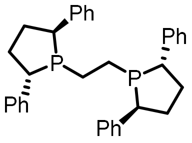

L* = (*R*,*R*)-Ph-BPE

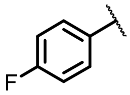

R = 4-fluorophenyl

All shown free energies are given in kcal·mol^{-1}
ωB97X-V/SMD(C_6H_6)/def2-QZVPP(Cu), def2-TZVPP(C,H,F,O,Si,P)//B3LYP-D3(BJ)/6-31G*(C,H,F,O,Si,P), SDD(Cu)
T = 298.15 K, p = 1.0 atm, qRRHO = 50 cm^{-1}, c = 1.0 M

Figure S54. Relative energies of isomers of copper alkoxides and higher aggregates of **1a**. A: Copper alkoxides interacting with one additional molecule of **1a** are predicted to always favor H-bonding to copper alkoxide oxygen over direct coordination of a **1a** oxygen to the copper center. B: Higher aggregation states of L*CuOR and two **1a** molecules. The most favorable aggregation mode for a given combination of enantiomers has its energy highlighted in green. All free energies are referenced to L*CuOR^{S} for direct comparability with the full mechanism shown in Figure 9 of the main manuscript.

7.3 Summary of Energies

Table S4. Summary of all computed species. All species are neutral, closed-shell singlets. All Energies are reported in Hartree units. Frequency units are reported in wavenumbers. Where applicable, only the lowest conformer found is reported. Values for H_{corr} and G_{corr} were obtained from GoodVibes (see Section 7.1).

Entry	Species	TS Freq.	EEl Opt	ZPE	H_{corr}	G_{corr}	EEl SP	EEl SP + G_{corr}
1	(*S*)-1a		-485.3450	0.1536	0.1633	0.1221	-485.3906	-485.2685
2	(*R*)-1a		-485.3450	0.1536	0.1633	0.1221	-485.3906	-485.2684
3	(*S*)-2a		-1247.9664	0.5162	0.5430	0.4600	-1247.9523	-1247.4924
4	(*R*)-2a		-1247.9664	0.5162	0.5430	0.4599	-1247.9523	-1247.4924
5	(*n*Bu)$_3$SiH		-763.7314	0.3791	0.3975	0.3327	-763.7019	-763.3691
6	styrene		-309.6694	0.1339	0.1412	0.1055	-309.6760	-309.5704
7	H_2		-1.1756	0.0101	0.0134	0.0017	-1.1724	-1.1707
8	PhEt		-310.9036	0.1576	0.1655	0.1286	-310.9110	-310.7825
9	(*S*)-**1a***(*S*)-**1a** Dimer		-970.7333	0.3095	0.3285	0.2634	-970.7954	-970.5321
10	(*S*)-**1a***(*R*)-**1a** Dimer		-970.7335	0.3095	0.3285	0.2638	-970.7953	-970.5315
11	(*R*)-**1a***(*R*)-**1a** Dimer		-970.7333	0.3095	0.3285	0.2634	-970.7954	-970.5321
12	L*Cu-(*R*)-Phen		-2507.9145	0.7607	0.7994	0.6901	-3950.8944	-3950.2043
13	L*Cu-(*S*)-Phen		-2507.9141	0.7611	0.8000	0.6911	-3950.8911	-3950.2000
14	L*CuH		-2198.1856	0.6197	0.6520	0.5579	-3641.1678	-3640.6099
15	L*CuH_Dimer		-4396.4532	1.2437	1.3067	1.1447	-7282.4111	-7281.2665
16	L*CuOR^{S}		-2682.3975	0.7575	0.7975	0.6844	-4125.4099	-4124.7255
17	L*CuOR^{R}		-2682.3957	0.7576	0.7979	0.6850	-4125.4062	-4124.7212
18	L*CuOR^{S}·(*S*)-**1a**		-3167.7937	0.9139	0.9630	0.8298	-4610.8293	-4609.9995
19	L*CuOR^{S}·(*R*)-**1a**		-3167.7976	0.9147	0.9640	0.8304	-4610.8301	-4609.9996
20	L*CuOR^{R}·(*S*)-**1a**		-3167.7980	0.9148	0.9641	0.8309	-4610.8288	-4609.9979
21	L*CuOR^{R}·(*R*)-**1a**		-3167.7902	0.9143	0.9634	0.8297	-4610.8253	-4609.9956
22	L*CuOR^{S}·(*S*)-**1a**_CuCoord		-3167.7811	0.9142	0.9641	0.8303	-4610.8129	-4609.9827
23	L*CuOR^{S}·(*R*)-**1a**_CuCoord		-3167.7873	0.9143	0.9638	0.8306	-4610.8211	-4609.9906
24	L*CuOR^{R}·(*S*)-**1a**_CuCoord		-3167.7889	0.9145	0.9642	0.8309	-4610.8219	-4609.9911
25	L*CuOR^{R}·(*R*)-**1a**_CuCoord		-3167.7905	0.9147	0.9645	0.8317	-4610.8223	-4609.9906
26	L*CuOR^{R}·(*R*)-**1a**·(*R*)-**1a**_Linear		-3653.1789	1.0714	1.1295	0.9761	-5096.2380	-5095.2619
27	L*CuOR^{R}·(*R*)-**1a**·(*S*)-**1a**_Linear		-3653.1760	1.0714	1.1295	0.9765	-5096.2373	-5095.2609
28	L*CuOR^{R}·(*S*)-**1a**·(*R*)-**1a**_Linear		-3653.1771	1.0703	1.1283	0.9739	-5096.2386	-5095.2647
29	L*CuOR^{R}·(*S*)-**1a**·(*S*)-**1a**_Linear		-3653.1752	1.0703	1.1286	0.9742	-5096.2385	-5095.2643
30	L*CuOR^{S}·(*R*)-**1a**·(*R*)-**1a**_Linear		-3653.1798	1.0719	1.1303	0.9772	-5096.2384	-5095.2613
31	L*CuOR^{S}·(*R*)-**1a**·(*S*)-**1a**_Linear		-3653.1819	1.0715	1.1299	0.9760	-5096.2409	-5095.2649
32	L*CuOR^{S}·(*S*)-**1a**·(*R*)-**1a**_Linear		-3653.1795	1.0702	1.1279	0.9727	-5096.2427	-5095.2700
33	L*CuOR^{S}·(*S*)-**1a**·(*S*)-**1a**_Linear		-3653.1783	1.0707	1.1286	0.9748	-5096.2437	-5095.2689
34	L*CuOR^{R}·(*R*)-**1a**·(*R*)-**1a**_CuCoord		-3653.1799	1.0711	1.1294	0.9766	-5096.2389	-5095.2623
35	L*CuOR^{R}·(*R*)-**1a**·(*S*)-**1a**_CuCoord		-3653.1746	1.0712	1.1293	0.9759	-5096.2350	-5095.2591
36	L*CuOR^{R}·(*S*)-**1a**·(*R*)-**1a**_CuCoord		-3653.1842	1.0706	1.1289	0.9759	-5096.2436	-5095.2678
37	L*CuOR^{R}·(*S*)-**1a**·(*S*)-**1a**_CuCoord		-3653.1783	1.0706	1.1287	0.9750	-5096.2393	-5095.2643
38	L*CuOR^{S}·(*R*)-**1a**·(*R*)-**1a**_CuCoord		-3653.1810	1.0709	1.1290	0.9752	-5096.2425	-5095.2672
39	L*CuOR^{S}·(*R*)-**1a**·(*S*)-**1a**_CuCoord		-3653.1818	1.0713	1.1299	0.9774	-5096.2411	-5095.2637
40	L*CuOR^{S}·(*S*)-**1a**·(*R*)-**1a**_CuCoord		-3653.1799	1.0701	1.1280	0.9735	-5096.2435	-5095.2700
41	L*CuOR^{S}·(*S*)-**1a**·(*S*)-**1a**_CuCoord		-3653.1787	1.0704	1.1280	0.9762	-5096.2420	-5095.2658
42	L*CuOR^{R}·(*R*)-**1a**·(*R*)-**1a**_OCoord		-3653.1795	1.0726	1.1310	0.9783	-5096.2365	-5095.2582
43	L*CuOR^{R}·(*R*)-**1a**·(*S*)-**1a**_OCoord		-3653.1827	1.0712	1.1295	0.9753	-5096.2429	-5095.2676
44	L*CuOR^{R}·(*S*)-**1a**·(*R*)-**1a**_OCoord		-3653.1845	1.0711	1.1296	0.9758	-5096.2420	-5095.2663
45	L*CuOR^{R}·(*S*)-**1a**·(*S*)-**1a**_OCoord		-3653.1842	1.0707	1.1288	0.9740	-5096.2439	-5095.2699
46	L*CuOR^{S}·(*R*)-**1a**·(*R*)-**1a**_OCoord		-3653.1863	1.0720	1.1301	0.9760	-5096.2461	-5095.2701
47	L*CuOR^{S}·(*R*)-**1a**·(*S*)-**1a**_OCoord		-3653.1766	1.0711	1.1289	0.9742	-5096.2389	-5095.2647
48	L*CuOR^{S}·(*S*)-**1a**·(*R*)-**1a**_OCoord		-3653.1732	1.0707	1.1286	0.9740	-5096.2351	-5095.2611
49	L*CuOR^{S}·(*S*)-**1a**·(*S*)-**1a**_OCoord		-3653.1811	1.0714	1.1296	0.9756	-5096.2425	-5095.2669
50	TS-I-(*S*)	-24.9	-3446.1461	1.1398	1.1970	1.0460	-4889.1140	-4888.0679
51	TS-I-(*R*)	-40.6	-3446.1446	1.1406	1.1977	1.0475	-4889.1097	-4888.0622
52	TS-II	-773.8	-2507.8684	0.7556	0.7938	0.6859	-3950.8528	-3950.1669
53	TS-II_Disfavored	-809.6	-2507.8564	0.7553	0.7939	0.6860	-3950.8394	-3950.1534
54	TS-III-(*S*)	-1329.4	-2993.2715	0.9126	0.9601	0.8318	-4436.2765	-4435.4447
55	TS-III-(*R*)	-1386.3	-2993.2687	0.9121	0.9598	0.8306	-4436.2721	-4435.4415
56	TS-IV-(*S*)	-1040.2	-2683.5503	0.7710	0.8116	0.6976	-4126.5613	-4125.8637
57	TS-IV-(*R*)	-1106.1	-2683.5488	0.7709	0.8118	0.6981	-4126.5581	-4125.8600
58	TS-L*CuOR^{R}·(*S*)-**1a**_ROHExchange	-542.6	-3167.7855	0.9100	0.9586	0.8257	-4610.8199	-4609.9942

8. Kinetic Modeling

8.1 General Considerations

Kinetic models were fitted to experimental data using standard numerical approaches. Competition models[S5] were fitted by minimizing the sum of square residues using Excel Solver. All absolute and relative rate constant values are given with errors rounded to the nearest tenth, reflecting an overestimation of uncertainty. All experimental fitted model parameters were obtained from simultaneous least-squares fitting of differential equations to temporal concentration data of (nBu)$_3$SiH, styrene, and (*S*)-**2a** across all employed datasets (Figures S56 and S57). This parameter combination represents a local solution to the overall reaction network. Computational model parameters were converted from the obtained standard state free energies (see Section 7.1 for details).

Dimerization of L*CuH is computationally predicted to be thermodynamically favorable ($\Delta G = -29.4$ kcal·mol^{-1}), but the transiency and low concentrations of L*CuH in the presence of styrene causes dimerization to be kinetically unfavorable. Under reaction conditions with styrene, kinetic simulations employing computationally derived parameters predict dimerization to be a negligible process, even when a theoretical rate constant for an irreversible dimerization is set to a value of $k_{\text{dim}} = 10^{11}$ M^{-1}·s^{-1}, at which point the reaction would be diffusion limited. Under reaction conditions without styrene, observed reaction stalling was modeled as a dimerization reaction where $k_{\text{dim}} = 0.3$ M^{-1}·s^{-1}, see Supporting Information Section 3.3.

Most datasets of **1a** were obtained with (*S*)-**1a**, but we note that the model is fully applicable to the (*R*)-**1a** enantiomer, as a relative value of k_1 is known for (*S*)-**1a** and (*R*)-**1a** ($k_S/k_R = 7.4$, see Section 4.5), and $K_{i,S}/K_{i,R} = 1.7$ is known from the relative $v_S/v_R = 4.3$ value and the relationship established in Equation S23 (see Section 3.1, Table S1). Furthermore, reliable estimates for K'_i and k_3 were obtained from the existing (*R*)-**1a** dataset (Figure S58).

In order to keep the model as simple as possible, and therefore at a minimum number of elementary reactions, off-cycle aggregation of (*S*)-**1a** was intentionally excluded from consideration.[S33] While there is some evidence for self-interaction of racemic **1b** as a pure liquid,[S33a] it is unlikely to be directly comparable to our system, which consists of benzene solutions of **1a** as well as other **1** derivatives. Consideration would require either accounting for oligomer reactivity, which would in effect multiply the number of elementary reactions involving (*S*)-**1a**, or result in inactive (*S*)-**1a** oligomers if no further elementary reactions are added, which would be inconsistent with experimental observations. A minimum number of elementary reactions is crucial in order to avoid overfitting due to a model that is too flexible. Therefore, a single elementary reaction to account for higher-order inhibition effects was

included in the model, denoted with the equilibrium constant K'_i. This in effect assumes that all rate and equilibrium constants involving (*S*)-**1a** represent a weighted average of reactivity across the speciation space of (*S*)-**1a** under experimental conditions. This compromise accounts for higher-order inhibition while only adding a single elementary reaction to the model. We note that together, the equilibria expressed by K_i and K'_i are equivalent either to the direct inhibition of L*CuOR^S by a hypothetical (*S*)-**1a** dimer, or by two single (*S*)-**1a** molecules.

Under conditions where the dominant catalyst speciation is L*CuOR^S·(*S*)-**1a**, the rate of (*S*)-**2a** generation is approximately governed by the k_1/K_i ratio. In this case, $[Cu]_0$ is approximated to consist mainly of L*CuOR^S·(*S*)-**1a** and L*CuOR^S. (Equation S15). The approximate macrokinetic reactivity as shown in Equation 2 of the main text results from the following derivation (Equations S13 to S22). Equations S22 and S23 are identical to Equations 1 and 2 in the main text.

$$K_i = \frac{[L * CuOR^S * (S) - \mathbf{1a}]}{[L * CuOR^S] \cdot [(S) - \mathbf{1a}]} \tag{S13}$$

$$[L * CuOR^S] = \frac{[L * CuOR^S * (S) - \mathbf{1a}]}{K_i \cdot [(S) - \mathbf{1a}]} \tag{S14}$$

$$[Cu]_0 \approx [L * CuOR^S * (S) - \mathbf{1a}] + [L * CuOR^S] = [L * CuOR^S * (S) - \mathbf{1a}] + \frac{[L * CuOR^S * (S) - \mathbf{1a}]}{K_i \cdot [(S) - \mathbf{1a}]} \tag{S15}$$

$$[Cu]_0 \approx [L * CuOR^S * (S) - \mathbf{1a}] \cdot \left(1 + \frac{1}{K_i \cdot [(S) - \mathbf{1a}]}\right) \tag{S16}$$

$$[Cu]_0 \approx [L * CuOR^S] \cdot K_i \cdot [(S) - \mathbf{1a}] \cdot \left(1 + \frac{1}{K_i \cdot [(S) - \mathbf{1a}]}\right) \tag{S17}$$

$$[Cu]_0 \approx [L * CuOR^S] \cdot K_i \cdot [(S) - \mathbf{1a}] + \frac{[L * CuOR^S] \cdot K_i \cdot [(S) - \mathbf{1a}]}{K_i \cdot [(S) - \mathbf{1a}]} \tag{S18}$$

$$[Cu]_0 \approx [L * CuOR^S] \cdot K_i \cdot [(S) - \mathbf{1a}] + [L * CuOR^S] \tag{S19}$$

$$[Cu]_0 \approx (K_i \cdot [(S) - \mathbf{1a}] + 1) \cdot [L * CuOR^S] \tag{S20}$$

$$[L * CuOR^S] \approx \frac{[Cu]_0}{1 + K_i \cdot [(S) - \mathbf{1a}]} \tag{S21}$$

$$\frac{d[(S) - \mathbf{2a}]}{dt} = v_S = k_1 \cdot [(nBu)_3SiH] \cdot [L * CuOR^S] \approx \frac{k_1 \cdot [(nBu)_3SiH] \cdot [Cu]_0}{1 + K_i \cdot [(S) - \mathbf{1a}]} \tag{S22}$$

(Equation S22 is identical to Equation 1 in the main manuscript)

$$v_S \approx \frac{k_1 \cdot [(nBu)_3SiH] \cdot [Cu]_0}{K_i \cdot [(S) - \mathbf{1a}]} = \frac{k_{obs} \cdot [(nBu)_3SiH]}{[(S) - \mathbf{1a}]} \text{; when } K_i \cdot [(S) - \mathbf{1a}] \gg 1 \tag{S23}$$

(Equation S23 is identical to Equation 2 in the main manuscript)

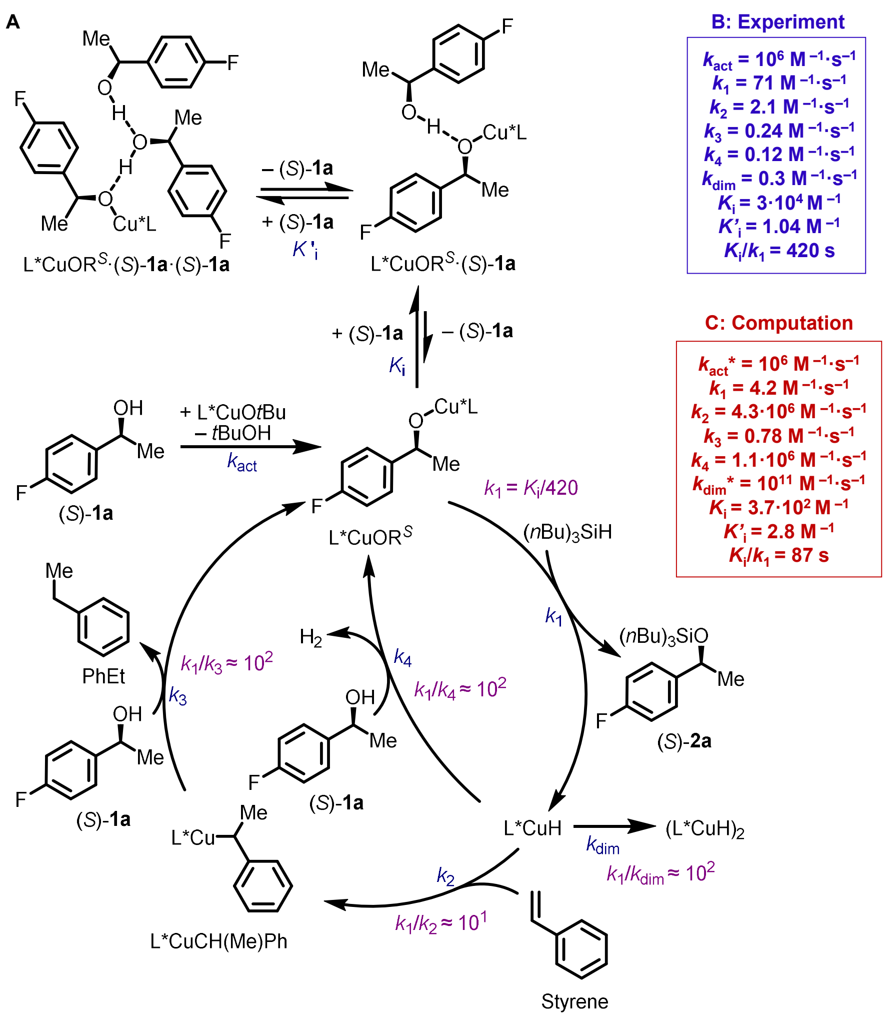

Figure S55. Minimal kinetic model for reactions of (*S*)-**1a**, the local solution to the overall reaction network used in this work in conjunction with experimental data (B), and the kinetic parameters resulting from computed standard-state free energies (C). Computational values marked with an asterisk (*) were arbitrarily defined and not directly obtained from calculations.

8.2 Experimental Data Fits

All entry numbers refer to the respective reactions in Table S1. Kinetic parameters used are those shown in Figure S55, B. The used parameter combination represents a local, not definitive solution to the overall reaction network.

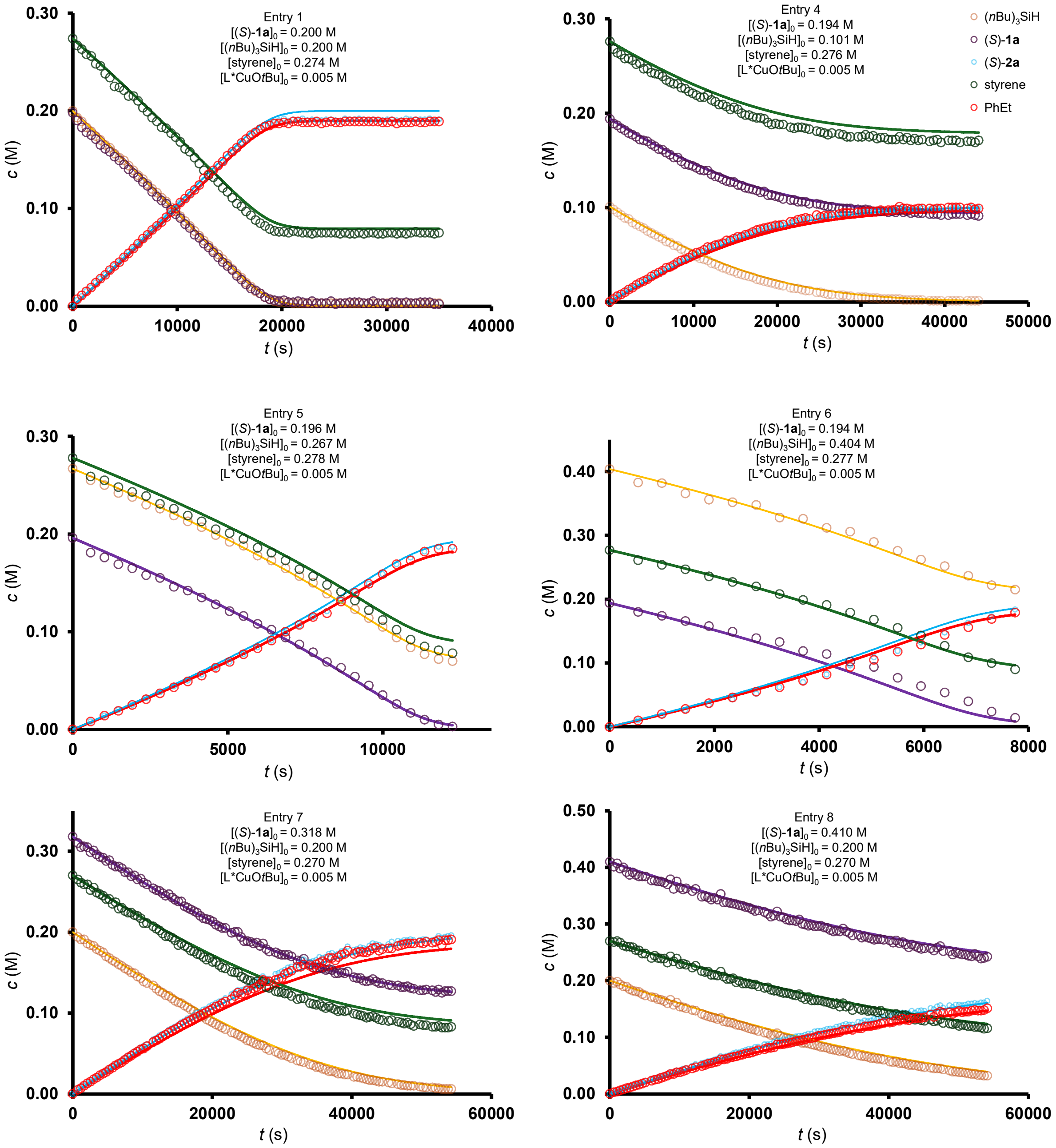

Figure S56. Comparison of experimental data (open circles) with kinetic simulation (lines) using the discussed kinetic model and experimental parameters (Figure S55, A and B). All entry numbers refer to the respective reactions in Table S1.

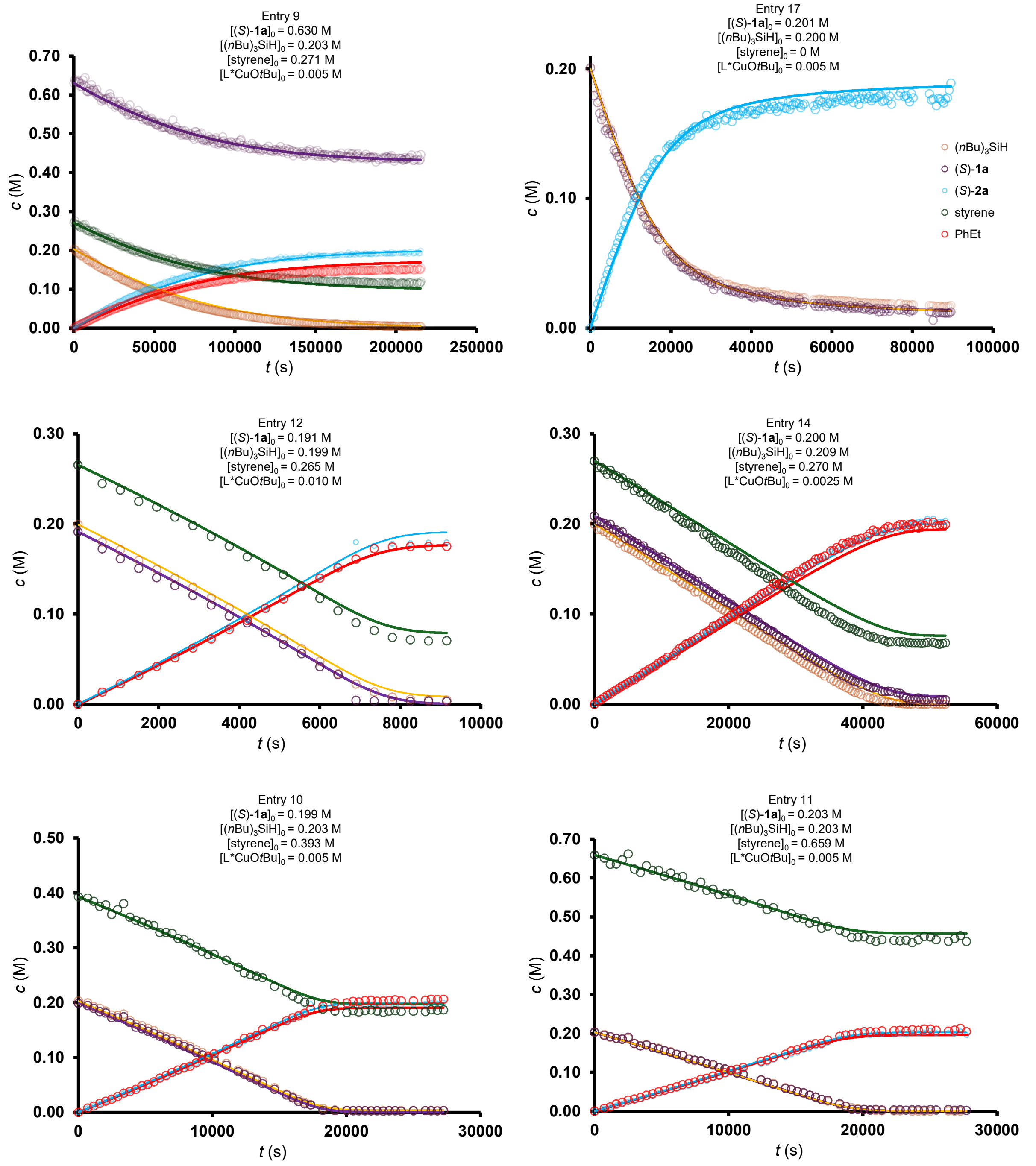

Figure S57. Comparison of experimental data (open circles) with kinetic simulation (lines) using the discussed kinetic model and experimental parameters (Figure S55, A and B). All entry numbers refer to the respective reactions in Table S1.

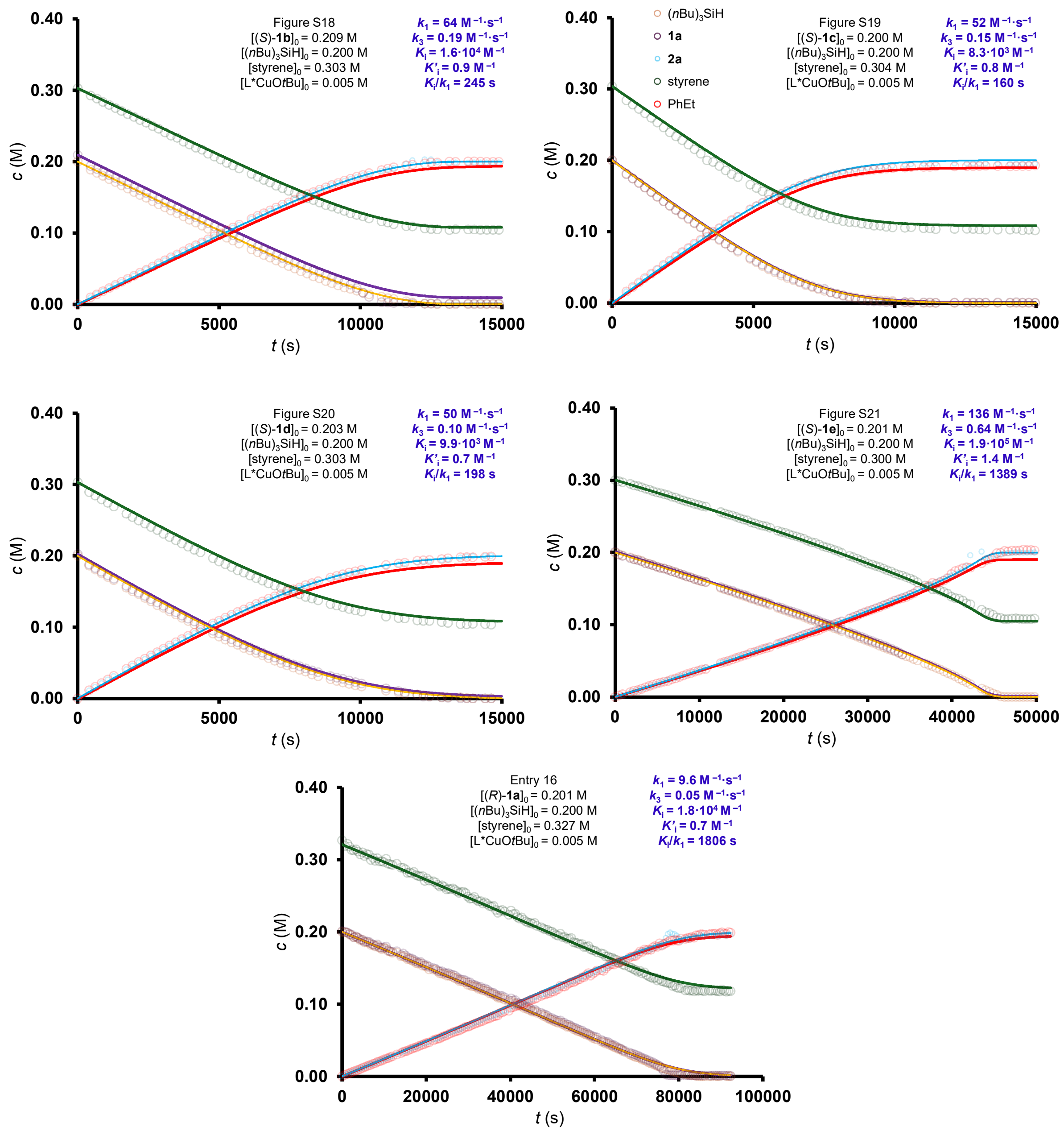

Figure S58. Comparison of experimental data (open circles) with kinetic simulation (lines) using the discussed kinetic model and experimental parameters (Figure S55, A and B). Entry number 16 refers to the respective reaction in Table S1. Experimental parameters which deviate from those given in Figure S55, B are depicted in blue in the respective plots. In all cases, k_1 and K_i were known (See Section 4.4), whereas K'_i and k_3 were fitted to the data.

8.3 Additional Boundary Conditions

Addition of incremental amounts of a solution containing L*CuO*t*Bu (0.1 M) and (*S*)-**1a** (0.05 M) to a solution of (*S*)-**1a** (0.05 M) enabled the relative estimation of the activation equilibrium constant associated with the displacement of *tert*-butanol by (*S*)-**1a**, K_{act}, in reference to the inhibition equilibrium constant K_i (see Section 8.1 for details).[S34] The observed ^{1}H and ^{19}F chemical shifts, respectively δ_{obs}, are timed-averaged functions of the chemical shifts of individual species and their mole fractions (Equation S24), and were jointly treated as a function of [L*CuO*t*Bu] via concurrent numerical fitting to a minimal equilibrium model as depicted in Figure S55.

$$\delta_{\mathrm{obs}} = \delta_{\mathrm{A}} \cdot \chi_{\mathrm{A}} + \delta_{\mathrm{B}} \cdot \chi_{\mathrm{B}} + 2 \cdot \delta_{\mathrm{C}} \cdot \chi_{\mathrm{C}} \qquad \text{(S24)}$$

K_i was determined to be larger than K_{act} by a factor of 3.4. As $K_i = 3{\cdot}10^4$ M^{-1}, $K_{act} = 9{\cdot}10^3$ M^{-1}. The large value of K_{act} and [(*S*)-**1a**]$_t$ >> [Cu]$_t$, which is fulfilled under most reaction conditions, together validate the choice of simplifying the kinetic model discussed in Section 8.1 to treat activation of L*CuO*t*Bu by (*S*)-**1a** as essentially irreversible.

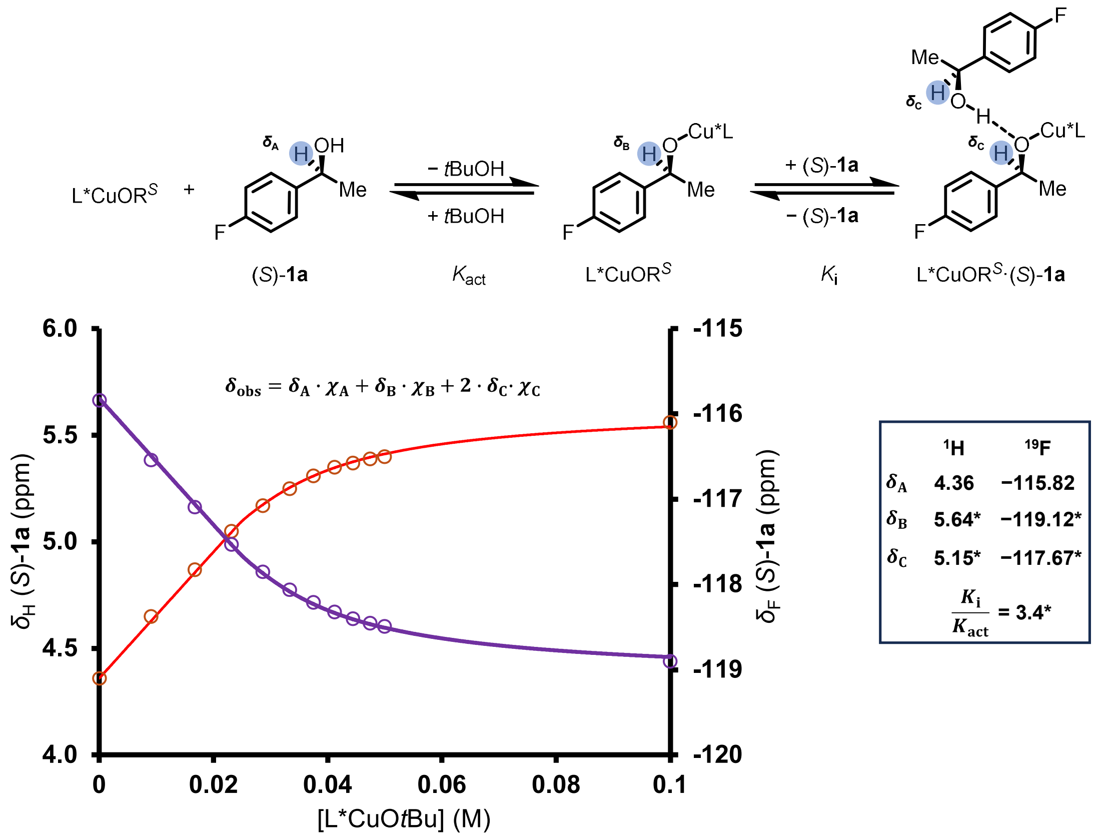

Figure S59. ^{1}H (red) and ^{19}F (purple) NMR chemical shift titration of the (*S*)-**1a** + L*CuO*t*Bu model system. Experimental data depicted as open circles and simulated δ_{obs} values depicted as continuous lines. Values next to an asterisk (*) are values fitted by the model. The relevant ^{1}H nuclei for the analysis are highlighted with blue circles.

Additionally, an order of magnitude estimate for the effective racemization rate constant, k_{rac}, under reaction conditions was modelled with the addition of the racemization equilibrium to a simulation employing parameters from Figure S55 for (*S*)-**1a**, from Figure S58, Entry 16, for (*R*)-**1a**, and addition of a fast, degenerate exchange equilibrium (*S*)-**1a** + L*CuOR^R <-> (*R*)-**1a** + L*CuOR^S. While this alkoxide exchange is unlikely to be degenerate, as L is a chiral ligand, the racemization model was found to be agnostic to the value of its equilibrium constant. The variation of **2a** *ee* with k_{rac} is shown in Figure S60, where it is shown that as k_{rac} approaches the boundary condition $k_{rac} \geq 400 \cdot k_1 \cdot K_i^{-1}$, the final **2a** *ee* is within 0.5% of the maximum theoretical *ee* value of 76.2% for $k_S/k_R = 7.4$. Furthermore, *ee* is shown to be virtually invariant to fractional conversion when $k_{rac} \geq 400 \cdot k_1 \cdot K_i^{-1}$, indicative of efficient DKR conditions.

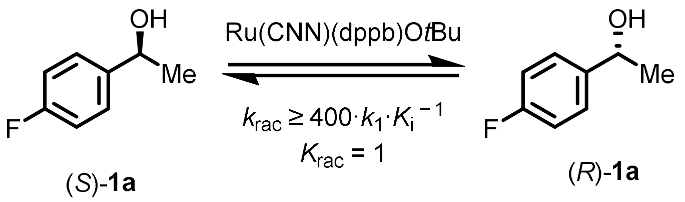

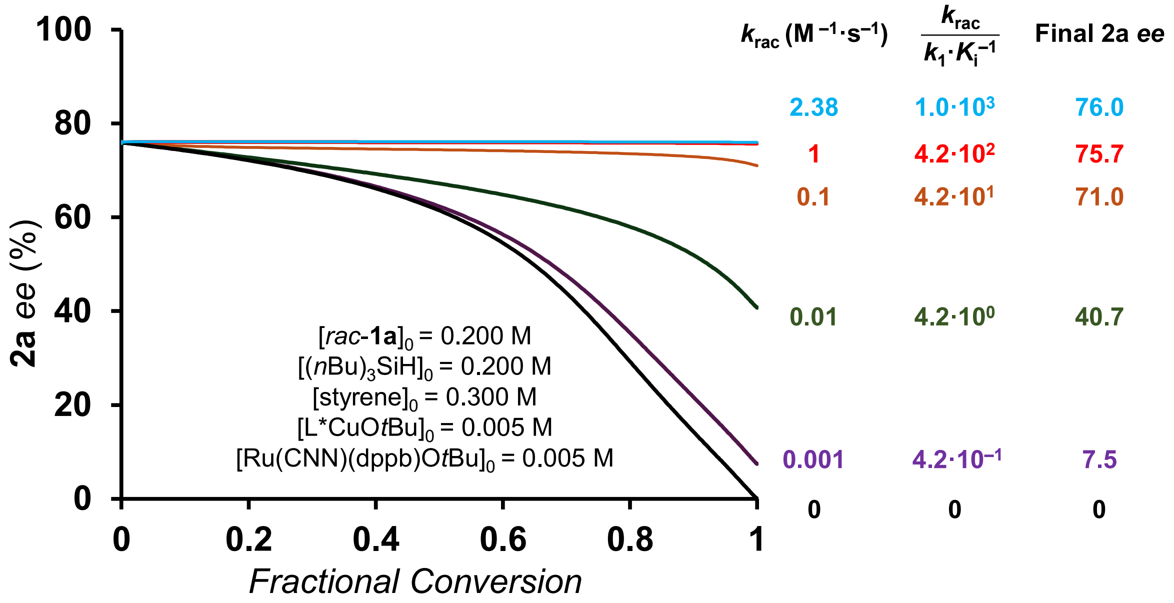

Figure S60. Simulations for the estimation of k_{rac} relative to $k_1 \cdot K_i^{-1}$ employing the kinetic model from Section 8.1 Experimental data depicted as open circles and simulated δ_{obs} values depicted as continuous lines. Values next to an asterisk (*) are values fitted by the model.

9. References

[S1] X. Dong, A. Weickgenannt, M. Oestreich, *Nat. Commun.* **2017**, *8*, 15547.

[S2] S. Kohrt, N. Santschi, J. Cvengroš, *Chem. Eur. J.* **2016**, *22*, 390–403.

[S3] J. F. Connolly, *J. Chem. Phys.* **1962**, *36*, 2897–2904.

[S4] M. Solinas, B. Sechi, S. Baldino, W. Baratta, G. Chelucci, *ChemistrySelect* **2016**, *1*, 2492–2497.

[S5] For details on the origin, derivations and uses of the Bigeleisen-Wolfsberg equations, see the Supporting Information in the following publication: H. J. A. Dale, A. G. Leach, G. C. Lloyd-Jones, *J. Am. Chem. Soc.* **2021**, *143*, 21079–21099.

[S6] C. Hansch, A. Leo and R. W. Taft, *Chem. Rev.* **1991**, *91*, 165–195.

[S7] C. J. Pilkington, A. Zanotti-Gerosa, *Org. Lett.* **2003**, *5*, 1273–1275.

[S8] M. W. Gribble Jr, R. Y. Liu, S. L. Buchwald, *J. Am. Chem. Soc.* **2020**, *142*, 11252–11269.

[S9] J. B. Johnson, J.-E. Bäckvall, *J. Org. Chem*. **2003**, *68*, 7681–7684.

[S10] Y. M. Badiei, T. H. Warren, *Inorg. Synth.* **2010**, *35*, 51–52.

[S11] F. Pape, N. O. Thiel, J. F. Teichert, *Chem. Eur. J.* **2015**, *21*, 15934–15938.

[S12] S. Rej, H. F. T. Klare, M. Oestreich, *Org. Lett*. **2023**, *25*, 2, 426–431.

[S13] Gaussian 16, Revision C.01, M. J. Frisch, G. W. Trucks, H. B. Schlegel, G. E. Scuseria, M. A. Robb, J. R. Cheeseman, G. Scalmani, V. Barone, G. A. Petersson, H. Nakatsuji, X. Li, M. Caricato, A. V. Marenich, J. Bloino, B. G. Janesko, R. Gomperts, B. Mennucci, H. P. Hratchian, J. V. Ortiz, A. F. Izmaylov, J. L. Sonnenberg, D. Williams-Young, F. Ding, F. Lipparini, F. Egidi, J. Goings, B. Peng, A. Petrone, T. Henderson, D. Ranasinghe, V. G. Zakrzewski, J. Gao, N. Rega, G. Zheng, W. Liang, M. Hada, M. Ehara, K. Toyota, R. Fukuda, J. Hasegawa, M. Ishida, T. Nakajima, Y. Honda, O. Kitao, H. Nakai, T. Vreven, K. Throssell, J. A. Montgomery, Jr., J. E. Peralta, F. Ogliaro, M. J. Bearpark, J. J. Heyd, E. N. Brothers, K. N. Kudin, V. N. Staroverov, T. A. Keith, R. Kobayashi, J. Normand, K. Raghavachari, A. P. Rendell, J. C. Burant, S. S. Iyengar, J. Tomasi, M. Cossi, J. M. Millam, M. Klene, C. Adamo, R. Cammi, J. W. Ochterski, R. L. Martin, K. Morokuma, O. Farkas, J. B. Foresman, and D. J. Fox, Gaussian, Inc., Wallingford CT, 2016.

[S14] Software update: the ORCA program system — Version 5.0 *Wiley Interdiscip. Rev.: Comput. Mol. Sci.,* **2022**, *12*, e1606.

[S15] C. Bannwarth, E. Caldeweyher, S. Ehlert, A. Hansen, P. Pracht, J. Seibert, S. Spicher, S. Grimme, *WIREs Comput. Mol. Sci.*, **2020**, *11*, e01493.

[S16] P. J. Stephens, F. J. Devlin, C. F. Chabalowski and M. J. Frisch, *J. Phys. Chem*. **1994**, *98*, 11623–11627.

[S17] S. Grimme, S. Ehrlich and L. Goerigk, *J. Comput. Chem*. **2011**, *32*, 1456–1465.

[S18] a) W. J. Hehre, R. Ditchfield and J. A. Pople, *J. Chem. Phys.* **1972**, *56*, 2257–2261. b) M. M. Francl, W. J. Pietro, W. J. Hehre, J. S. Binkley, M. S. Gordon, D. J. DeFrees, J. A. Pople, *J. Chem. Phys*. **1982**, *77*, 3654–3665. c) V. A. Rassolov, J. A. Pople, M. A. Ratner, and T. L. Windus, *J. Chem. Phys*. **1998**, *109*, 1223–1229.

[S19] Andrae, D.; Haeussermann, U.; Dolg, M.; Stoll, H.; Preuss, H. *Theor. Chem. Acc.* **1990**, *77*, 123–141.

[S20] K. Ishida, K. Morokuma and A. Komornicki, *J. Chem. Phys*. **1977**, *66*, 2153–2156.

[S21] Luchini, G.; Alegre-Requena, J. V.; Funes-Ardoiz, I.; Paton, R. S. *F1000Research*, **2020**, *9*, 291.

[S22] P. Pracht, S. Grimme, C. Bannwarth, F. Bohle, S. Ehlert, G. Feldmann, J. Gorges, M. Müller, T. Neudecker, C. Plett, S. Spicher, P. Steinbach, P. Wesołowski, F. Zeller*, J. Chem. Phys.* **2024**, *160*, 114110.

[S23] N. Mardirossian, M. Head-Gordon, *Phys. Chem. Chem. Phys.* **2014**, *16*, 9904–9924.

[S24] F. Weigend and R. Ahlrichs, *Phys. Chem. Chem. Phys*. **2005**, *7*, 3297–3305.

[S25] A. V. Marenich, C. J. Cramer and D. G. Truhlar, *J. Phys. Chem. B* **2009**, *113*, 6378–6396.

[S26] F. Weigend, *Phys. Chem. Chem. Phys*. **2006**, *8*, 1057–1065.

[S27] B. Helmich-Paris, B. De Souza, F. Neese, R. Izsák, *J. Chem. Phys.* **2021**, *155*, 104109.

[S28] F. Neese, *J. Comp. Chem.,* **2003**, *24*, 1740–1747.

[S29] Anderson, T. L., Kwan, E. E. *PyQuiver* **2020**, www.github.com/ekwan/PyQuiver.

[S30] L. Zhang, Y. Tang, Z. Han, K. Ding, *Angew. Chem. Int. Ed.* **2019**, *58*, 4973–4977.

[S31] R. Buhaibeh, O. A. Filippov, A. Bruneau-Voisine, J. Willot, C. Duhayon, D. A. Valyaev, N. Lugan, Y. Canac, J.-B. Sortais*, Angew. Chem. Int. Ed.* **2019**, *58*, 6727–6731.

[S32] For examples of the synthesis and stability of Cu(I)-alkoxides where no phosphine ligand is present, see: a) G. M. Whitesides, J. S. Sadowski, J. Lilburn, *J. Am. Chem. Soc.* **1974**, *96*, 2829–2835, b) M. Bochmann, G. Wilkinson, G. B. Young, M. B. Hursthouse, K. M. A. Malik, *J. Chem. Soc., Dalton Trans.* **1980**, 1863–1871.

[S33] For examples of alcohol aggregation as pure liquids or in organic solvents, see: a) A. Nowok, K. Jurkiewicz, M. Dulski, H. Hellwig, J. G. Małecki, K. Grzybowska, J. Grelska, S. Pawlus, *Journal of Molecular Liquids* **2021**, *326*, 115349. b) P. Sassi, F. Palombo, R. S. Cataliotti, M. Paolantoni, A. Morresi, *J. Phys. Chem. A* **2007**, *111*, 6020–6027.

[S34] For details on NMR chemical shift titrations, see: Ben-Tal, Y.; Boaler, P. J.; Dale, H. J. A.; Dooley, R. E.; Fohn, N. A.; Gao, Y.; García-Domínguez, A.; Grant, K. M.; Hall, A. M. R.; Hayes, H. L. D.; Kucharski, M. M.; Wei, R.; Lloyd-Jones, G. C. *Progress in Nuclear Magnetic Resonance Spectroscopy* **2022**, *129*, 28–106.
